# Gut microbiota and its diet‐related activity in children with intestinal failure receiving long‐term parenteral nutrition

**DOI:** 10.1002/jpen.2188

**Published:** 2021-08-25

**Authors:** Esther G. Neelis, Barbara A. E. de Koning, Jessie M. Hulst, Rodanthi Papadopoulou, Caroline Kerbiriou, Edmond H. H. M. Rings, René M. H. Wijnen, Ben Nichols, Konstantinos Gerasimidis

**Affiliations:** ^1^ Department of Pediatric Gastroenterology Erasmus Medical Center–Sophia Children's Hospital Rotterdam The Netherlands; ^2^ Department of Pediatrics, Division of Gastroenterology, Hepatology and Nutrition The Hospital for Sick Children Toronto Ontario Canada; ^3^ Human Nutrition, School of Medicine, College of Medicine, Veterinary and Life Sciences University of Glasgow Glasgow United Kingdom; ^4^ Department of Pediatric Gastroenterology Leiden University Medical Center–Willem Alexander Children's Hospital Leiden The Netherlands; ^5^ Department of Pediatric Surgery Erasmus Medical Center–Sophia Children's Hospital Rotterdam The Netherlands

**Keywords:** enteral nutrition, gut microbiota, intestinal failure, microbiome, parenteral nutrition, short bowel syndrome

## Abstract

**Background:**

This study characterized gut microbiota and its diet‐related activity in children with intestinal failure (IF) receiving parenteral nutrition (PN) compared with those of healthy controls (HC) and in relation to disease characteristics.

**Methods:**

The fecal microbiota and short‐chain fatty acids (SCFAs) were measured in 15 IF patients (n = 68) and 25 HC (n = 25).

**Results:**

Patients with IF had a lower bacterial load (*P* = .003), diversity (*P* < .001), evenness (*P* < .001) and richness (*P* = 0.006) than HC. Patients with surgical IF had lower diversity (*P* < .039) than those with functional IF. Propionic acid and butyric acid (*p* < .001) were lower and d‐lactate and l‐lactate were higher (*p* < 0.001) in IF patients than in HC. The energy supplied by PN (%PN) was negatively associated with microbiota diversity and SCFA profile. IF patients had more *Escherichia*‐*Shigella* (*P* = .006), *Cronobacter* (*P* = .001), and *Staphylococcus* (Operational Taxonomic Unit 14, *P* < .001) and less *Faecalibacterium* (*P* < 0.001) and *Ruminococcus* 1 and 2 (*P* < .001). Duration of PN (*P* = .005), %PN (*P* = .005), and fiber intake (*P* = .011) were predictive of microbiota structure. Higher intake of enteral nutrition was associated with microbiota structure and function closer to those of HC.

**Conclusions:**

Microbiota composition and its diet‐related function are altered in IF, with depletion of beneficial SCFAs and species and supraphysiological increase of potentially harmful pathobionts. The influence of this compositional and functional microbial dysbiosis on patients’ outcomes and management warrants further exploration.

## CLINICAL RELEVANCY STATEMENT

The gut microbiota of children with IF is compositionally and functionally dysbiotic, with major shifts toward potentially harmful bacteria and depletion of beneficial species. Short‐chain fatty acids (SCFAs), which are of critical importance to gut health, were depleted, whereas the concentration of their precursor molecule, lactate, which has been associated with risk of d‐lactic acidosis, was excessively high. The influence and therapeutic modulation of such microbial alterations are likely to have implications for disease management and prognosis during the process of gut adaptation.

## INTRODUCTION

Patients with intestinal failure (IF) cannot absorb enough nutrients and fluids to sustain life. ^1^, ^2^ This is either because their intestine is too short, as a consequence of major surgical resection, or due to loss of intestinal function. Patients with IF are therefore dependent on parenteral nutrition (PN) to survive.

Gut microbiota plays a key role in human health that extends beyond the gut to whole‐body homeostasis.^3^, ^4^ Previous studies have reported an altered gut microbiota composition in patients with IF, including a decrease in bacterial diversity^5^, ^6^, ^7^ and an increase in the abundance of pathogens.^5^, ^6^, ^7^, ^8^, ^9^, ^10^ Absence of luminal substrate essential for bacterial growth and sequential “gut starvation” in patients receiving PN are likely to alter the production of SCFAs, which are important products of fiber fermentation that indirectly contribute energy to the host and stimulate gut vascular flow and motility, cell proliferation, and cell differentiation.^7^, ^11^, ^12^, ^13^ However, data on SCFA production in IF patients are scarce. In IF, gut microbiota has been associated with adverse clinical outcomes such as bacterial translocation, d‐lactic acidosis, central line–associated bloodstream infection, poor growth, and liver disease.^6^, ^7^, ^10^ Most of the previous literature relied on single samples, with a lack of studies reporting serial sample collection and changes during IF management. Additionally, previous research focused on children with short bowel syndrome (SBS), and information about children with functional IF is lacking.^6^ It is of interest to compare the microbiota characteristics of patients with differences in gastrointestinal anatomy and function, because changes in the gut microbiota characteristics may offer opportunities to use these as a prognostic or therapeutic target. The aim of this study was to characterize the microbiota composition and its diet‐related function in samples of children with surgical and functional IF, over time, and relate these with disease characteristics and compare these with healthy controls.

## MATERIALS AND METHODS

### Study population

Children who were stable while receiving home PN (>3 months) and were attending the IF team of the Erasmus Medical Center–Sophia Children's Hospital participated in this prospective observational study. Healthy Dutch children were recruited from the local area through advertisement and acted as healthy controls. None of the healthy controls had undergone gastrointestinal surgery, and none of them had received antibiotics for ≥2 months prior to sample collection. The study was approved by the local research ethical committees (MEC 2015‐002, Dutch Trial Register NTR6080, https://www.trialregister.nl/), and informed consent was obtained from the patients, healthy controls, and/or their parents.

### Clinical data

Patients were divided into surgical IF (including patients with SBS and patients who had a minor resection of the small bowel but did not fulfill the criteria for SBS) and functional IF (including motility disorders and enteropathies). SBS was defined as a resection of >70% of the small intestine and/or a remaining length of the small intestine (measured from the ligament of Treitz onward) of <50 cm in preterm infants or <75 cm in term infants.^14^

Demographic and clinical data (eg, underlying disease, duration of PN) were obtained from medical records. Height standard deviation scores (SDS), body mass index (BMI) SDS, target height, and target height range (±1.6 SDS) were calculated as described previously, using the latest Dutch reference standards.^15^, ^16^, ^17^ Percentage energy intake from PN was used as a measure of PN dependency. In addition, we calculated the energy from PN provided as a ratio of predicted resting energy expenditure.^18^ Oral nutrition was defined as table food or breast milk/formula taken orally.

### Fecal sample collection

Fecal samples were collected from the diaper or the enterostomy or by using a “feces hat.” We collected samples longitudinally over 2 years, aiming at collecting samples every 3 months if patients were visiting the outpatient clinic. A single fecal sample was collected from healthy controls. Samples were stored at −80 °C, and DNA was extracted within a maximum of 2 months of collection. For SCFA analysis, samples were stored in NaOH 1 M wt/vol at −20 °C until analysis. Fecal water content was calculated following lyophilization.

### Fecal lactate

d and l isomers of lactic acid were measured in freeze‐dried fecal samples using a commercial assay (Boehringer Mannheim Roche) scaled down for use with a 96‐well plate.

### Short‐chain and branched‐chain fatty acids

SCFAs (C2–C8) were measured by gas chromatography.^19^ Results were presented per gram of dry mass of fecal material (µmol/g) and as proportion (%) to total SCFA.

### Microbiota

The composition of the gut microbiota was characterized with amplicon sequencing of the V4 region of the 16S ribosomal RNA (rRNA) gene.^20^ Genomic DNA was isolated using the bead‐beating method coupled with the chaotropic method.^19^, ^21^ Quantification of total bacterial load (total 16S rRNA gene copy number per gram of feces) was carried out with quantitative polymerase chain reaction (qPCR).^19^

### Bioinformatics

Microbiota composition was analyzed using operational taxonomic units (OTUs) obtained from the raw 16S rRNA sequencing data and clustered at a level of 97% similarity by using a modified version of the VSEARCH pipeline (https://github.com/torognes/vsearch/wiki/VSEARCH‐pipeline).^22^ OTUs were taxonomically classified to genus level using the assignTaxonomy function in the dada2 R package.^23^

### Data analysis and statistics

Descriptive statistics were expressed as median and interquartile range (IQR) or range or as counts with percentages. Data are presented separately for the first sample collected as well as for all samples together, correcting for repeated measurements per participant. For group comparisons, Mann‐Whitney *U*, chi‐squared, and Fisher exact tests were used. Statistical analyses on microbiota structure were performed using the phyloseq^27^ and vegan^28^ packages in R. Significantly different bacterial taxa were identified using *t*‐tests on the log‐proportional abundances of each OTU/genus, with paired *t*‐tests used when comparing repeated samples from the same participant. Generalized linear mixed models were used to identify relationships between disease characteristics and microbial diversity measures using the lme4 package in R.^24^ Benjamini‐Hochberg corrections were applied for all cases of multiple testing. *P*‐values <.05 and adjusted *P*‐values <.1 were considered statistically significant. Adjusted *P*‐values are mentioned in the manuscript. Statistical analysis was performed using SPSS version 21 (SPSS, IBM) and R version 3.4.3.

## RESULTS

### Participants’ characteristics

Fifteen patients (median age, 4.3 years; range, 0.7–16.6) were enrolled between June 2015 and September 2017. Twenty‐five healthy controls were recruited whose age, BMI SDS, and gender characteristics were comparable to those of IF patients. Participants’ characteristics are shown in Table 1. Eight patients had surgical and seven patients had functional IF. Underlying diseases in surgical IF included intestinal atresia (n = 3), gastroschisis (n = 2), necrotizing enterocolitis (n = 2), and herniation and strangulation of the small bowel (n = 1). In functional IF, patients experienced chronic intestinal pseudo‐obstruction syndrome (n = 1), microvillus inclusion disease (n = 1), protein‐losing enteropathy based on primary intestinal lymphangiectasia (n = 1), tricho‐hepato‐enteric syndrome (n = 1), filamin A mutation with pseudo‐obstruction (n = 1), and esophageal atresia with motility problems (n = 1), in addition to one unknown cause.

**Table 1:** Participants’ characteristics at first sample collection for all IF patients, divided into surgical and functional IF, and healthy controls

**Participants’ characteristics**	**All IF, n = 15**	**Surgical IF, n = 8**	**Functional IF, n = 7**	**Healthy controls, n = 25**
Sex, boys:girls	8:7 (53:47)	5:3 (63:38)	3:4 (43:57)	13:12 (52:48)
Age at first sample, years	4.3 (0.7–16.6)	6.1 (0.7–9.9)	3.7 (0.7–16.6)	6.6 (1.1–15.4)
Whole small bowel in situ	5 (33)	0 (0)	5 (71)	
Remaining small ‐bowel length, cm	65 (30–180)	63 (46–103)	180 (NA)^a^	
Ileocecal valve in situ	9 (60)	2 (25)	7 (100)	
Enterostomy	1 (7)	0 (0)	1 (14)	
Partial or total colectomy	5 (33)	4 (50)	1 (14)	
Duration of PN until first sample, years	3.6 (2.0–5.0)	4.4 (1.1–7.3)	3.2 (2.0–4.3)	
PN dependency (% of total energy intake)	76 (40–100)	62 (38–87)	82 (67–100)	
Type of nutrition^b^				
PN only	4 (27)	1 (13)	3 (43)	
PN and tube feeding	7 (47)	4 (50)	3 (43)	
PN and oral nutrition	1 (7)	0 (0)	1 (14)	
PN and tube feeding/oral nutrition	3 (20)	3 (38)	0 (0)	
Mode of tube feeding^b^				
Continuous	6 (40)	3 (38)	3 (43)	
Bolus	3 (20)	3 (38)	0 (0)	
Combination of continuous and bolus	1 (7)	1 (13)	0 (0)	
Type of tube feeding				
Polymeric	2 (13)	2 (25)	0 (0)	
Semielemental	7 (47)	5 (63)	2 (29)	
Elemental	1 (7)	0 (0)	1 (14)	
Antibiotic use 2 months before first sample	12 (80)	8 (100)	4 (57)	0 (0)
Proton pomp inhibitor use	11 (73)	5 (63)	6 (86)	0 (0)
BMI SDS	0.34 (−0.11–1.29)	0.03 (−0.63–0.53)	1.14 (0.40–1.48)	0.07 (−0.67–0.77)

*Note*: Values shown as median (interquartile range) or n (%) unless stated otherwise.

Abbreviations: BMI SDS, body mass index standard deviation score; IF, intestinal failure; NA, not applicable; PN, parenteral nutrition.

^a^For one patient, the small‐bowel length was not known.

^b^Minimal enteral feeding not included.

Four patients underwent surgical lengthening procedures (none in the year prior to sample collection). All patients were PN dependent at the first sample collection, with a median PN duration of 3.6 years. Twelve patients had received antibiotics in the 2 months prior to the first sample collection; of these, 8 (of 8) had surgical IF and 4 (of 7) had functional IF (*P* = .04). Two patients received enteral/oral antibiotic treatment because of suspected bacterial overgrowth, and three patients received enteral/oral antibiotic treatment as a prokinetic agent. None of the patients received probiotics, nor did patients develop d‐lactic acidosis or IF‐associated liver disease. The median follow‐up period of each patient was 14 months (IQR, 10–21; range, 4–23).

### Gut microbiota in IF patients and healthy controls

For one patient, samples (n = 2) were inadequate for sequencing, but bacterial metabolites are reported. Thus, we extracted genomic DNA from 66 fecal samples from 14 patients and 25 healthy controls. Four IF samples could not be amplified. All samples had >5000 reads per sample, and following OTU clustering, 1129 unique OTUs were assigned across all 87 samples.

**Figure 1:**
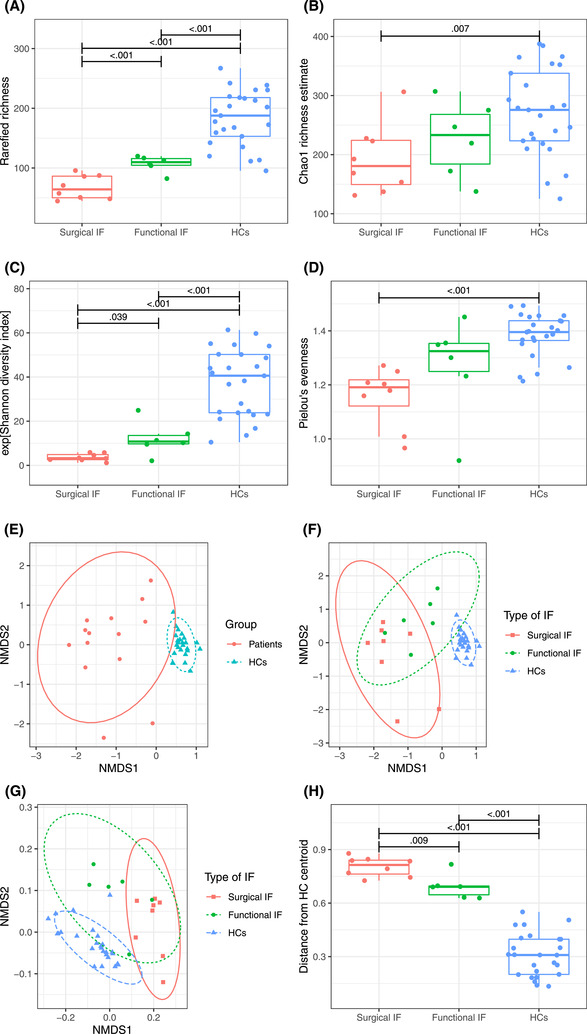
(A–D) Alpha diversity metrics in patients with surgical (n = 8) and functional (n = 7) intestinal failure (IF) and healthy controls (HCs, n = 25). (E–F) Nonmetric multidimensional scaling (NMDS) of operational taxonomic unit (OTU) community structures for (E) IF patients (n = 15) and HCs (n = 25) and (F) surgical (n = 8) and functional (n = 7) IF patients and HCs at first sample collection. Samples that are clustered closely together are more similar in terms of bacterial taxon composition than samples that are more separated. (G) Weighted UniFrac NMDS of OTU community structures for surgical and functional IF patients and HCs at first sample collection. (H) Bray‐Curtis distances from the group centroid of HCs for surgical and functional IF patients at the first sample and HCs

Total bacterial load (*P* = .003), Shannon diversity (*P* < .001), and taxon richness (Chao richness, *P* = .006) and evenness (Pielou's evenness, *P* < .001) were lower in patients with IF than in healthy controls (Figure 1A–D). By using the Bray‐Curtis dissimilarity index, the microbiota community structure (beta diversity) of IF patients was found to be distinct from that of healthy controls, presenting a higher extent of interindividual variation (*P* = .002) (Figure 1E,F). Similar findings were observed by using weighted UniFrac distances (Figure 1G).

**Figure 2:**
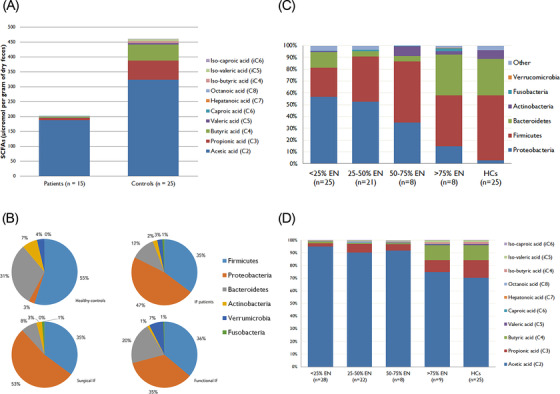
(A) Stacked bar chart displaying the median levels of short‐chain fatty acids (SCFAs, µmol per gram of dry feces) for intestinal failure (IF) patients and healthy controls. (B) Pie charts representing the major bacterial phyla for surgical, functional, and all IF patients and healthy controls (all IF patient samples included). (C) Composition of the gut microbiota at phylum level and (D) proportional abundance of SCFAs and branched‐chain fatty acids according to the proportion of energy delivered from enteral nutrition (EN) at the time of sample collection

**Figure 3:**
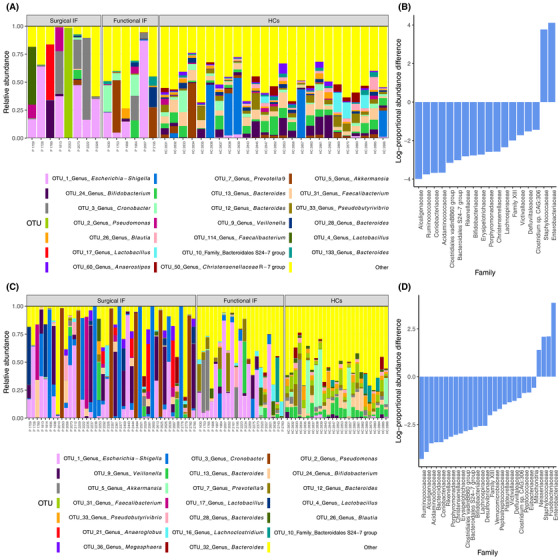
(A) Taxonomic composition of microbiota of pediatric IF patients (P, n = 15) and healthy controls (HCs, n = 25) at the operational taxonomic unit (OTU) level for the 20 most abundant OTUs at first sample collection. (B) Relative abundance of differential bacterial families of patients with IF and HCs. (C) Taxonomic composition of microbiota of pediatric IF patients and HCs at the OTU level for the 20 most abundant OTUs for all IF patient samples. (D) Microbial communities in patients with IF showing relative abundance of the most common taxonomic families for all IF patient samples

Bacteria in the fecal samples from patients included those from the six dominant phyla of the human gut microbiota. However, the relative abundance of several phyla was remarkably different when compared with healthy controls. IF patients had increased abundance of Proteobacteria and a decreased abundance of Bacteroidetes and Verrucomicrobia (Figure 2). At the family level, patients had more Enterobacteriaceae (*P* = .001), Staphylococcaceae (*P* = .001), Bacteroidaceae (*P* = .013), and Bifidobacteriaceae (*P* = .004) (Figure S1). At the genus level, the microbiota of IF patients was characterized by a higher abundance of *Escherichia*‐*Shigella* (*P* = .006), *Cronobacter* (*P* = .001), and *Staphylococcus* (OTU 14, *P* < .001) than healthy controls had (Figure 3). In contrast, IF patients had a lower abundance of species belonging to *Faecalibacterium* (OTU 114 and 31, *P* < .001) and *Ruminococcus* 1 and 2 (OTU 83, 167, 262, 42, 119, and 64; *P* < .001). Repeating the analysis while including all IF patient samples produced similar results to those with the first sample collected (Tables S1 and S2).

### SCFA and lactate in IF patients and healthy controls

Sixty‐eight samples were collected (median of three samples per patient; IQR, 2–6; range, 1–10). Because patients with IF had significant higher water content, we chose to express data per gram of dry feces. At the first sample collected, IF patients had lower concentrations of total SCFAs (210 vs 472 µmol/g, *P* = .008), propionic acid (7.7 vs 64 µmol/g, *P* < .001), and butyric acid (2.0 vs 54.3 µmol/g, *P* < .001) than healthy controls had (Table 2, Figure 1). Acetic acid concentration tended to differ between IF patients and controls, and its proportional abundance was higher in patients than in healthy controls (*P* < .001). Patients had a 10‐fold higher concentration of d‐lactate and l‐lactate than healthy controls (*P* = .004 and *P* = .041, respectively). When data were expressed per wet mass, results remained similar (Table S3). Likewise, similar results were obtained when comparing the last sample collected from each participant with those of the healthy controls (Table S3).

**Table 2:** Fecal water content, concentration of SCFAs, lactate, and total bacterial load (16S rRNA gene copies/g) for IF patients and healthy controls at first sample collection

	**n**	**Patients with IF, n = 15**	**n**	**Healthy controls, n = 25**	***P*‐value**
Fecal water content, %	14	83 (66–87)	25	65 (62–74)	.011
SCFA per g^a^	15		25		
Acetic acid (C2), µmol/g		188 (86.8–515)		323 (266–370)	.074
Acetic acid (C2), %		91.8 (83.4–94.4)		67.6 (64.7–61.3)	<.001
Propionic acid (C3), µmol/g		7.73 (1.03–18.6)		64.0 (47.5–85.1)	<.001
Propionic acid (C3), %		3.64 (1.19–8.15)		13.7 (10.6–18.8)	<.001
Butyric acid (C4), µmol/g		2.04 (1.08–18.4)		54.3 (36.6–71.0)	<.001
Butyric acid (C4),%		0.96 (0.73–4.10)		11.6 (5.60–14.9)	<.001
Valeric acid (C5), µmol/g		0.19 (0.11–5.66)		4.94 (2.04–9.56)	.001
Valeric acid (C5), %		0.18 (0.07–0.65)		1.39 (0.36–2.26)	.002
Caproic acid (C6), µmol/g		0.45 (0.33–0.62)		0.51 (0.26–3.72)	.046
Caproic acid (C6), %		0.21 (0.07–0.39)		0.12 (0.08–0.78)	.912
Heptanoic acid (C7), µmol/g		0.71 (0.44–0.87)		0.07 (0.04–0.17)	.001
Heptanoic acid (C7), %		0.39 (0.11–0.54)		0.01 (0.00–0.05)	<.001
Octanoic acid (C8), µmol/g		0.09 (0.00–0.68)		0.17 (0.04–0.34)	.659
Octanoic acid (C8), %		0.05 (0.00–0.37)		0.01 (0.01–0.08)	.761
Total, µmol/g		210 (103–618)		472 (397–592)	.008
Iso‐butyric acid (iC4), µmol/g		0.82 (0.18–3.67)		6.40 (3.73–10.2)	<.001
Iso‐butyric acid (iC4), %		0.21 (0.13–1.00)		1.42 (0.92–2.17)	.003
Iso‐valeric acid (iC5), µmol/g		1.05 (0.11–5.66)		6.27 (3.48–10.2)	<.001
Iso‐valeric acid (iC5), %		0.44 (0.13–1.05)		1.18 (0.82–14.9)	.006
Iso‐caproic acid (iC6), µmol/g		0.46 (0.18–1.05)		0.35 (0.26–0.48)	.201
Iso‐caproic acid (iC6), %		0.18 (0.09–0.31)		0.07 (0.05–0.09)	.002
d‐lactate, mcg/g^a^	8	1815 (485–7107)	24	79 (58–156)	<.001
l‐lactate, mcg/g^a^	8	1923 (464–3675)	24	211 (102–257)	<.001
Total lactate, mcg/g^a^	8	3739 (898–11157)	24	256 (193–376)	<.001
% d‐lactate per g^a^	8	48 (42–57)	24	33 (19–50)	.023
Log of 16S rRNA gene copy number per g^a^ (IQR, range)	14	10.7 (9.92–10.9, 0.53–11.5)	25	11.1 (10.9–11.3, 10.7–11.7)	.003
Log of 16S rRNA gene copy per g^b^ (IQR, range)	14	1.96 (1.60–3.87, 1.28–9.93)	25	3.82 (3.33–4.37, 2.6–18.4)	.015

*Note*: Values shown as median (IQR) or n (%) unless stated otherwise.

Abbreviations: IF, intestinal failure; IQR, interquartile range; rRNA, ribosomal RNA; SCFA, short‐chain fatty acid.

^a^Gram of dry feces.

^b^Gram of wet feces.

### Differences between surgical and functional IF patients

Between the two types of IF, there was no significant difference in total bacterial load. However, patients with surgical IF had a lower Shannon diversity (*P* = .039) and rarefied richness (*P* < .001) than did patients with functional IF. Within the IF group, patients with surgical IF clustered separately from patients with functional IF (*P* = .009), whose community structure was more similar to that of healthy controls (Figure 2H).

Surgical IF patients had an increased abundance of Proteobacteria and a decreased abundance of Verrucomicrobia compared with functional IF patients. There were no significant differences in taxon abundance at the family or OTU level between patients with surgical and patients with functional IF, at first sample collection. When comparing surgical with functional IF for all patient samples, the former group had a higher abundance of taxa belonging to *Lactobacillus* (OTU 18, 166, and 201, *P* = .003; OTU 38, *P* = .019; OTU 17, *P* = .020; OTU 4, *P* = .037) and *Cronobacter* (*P* = .020), whereas functional IF patients had a higher abundance of taxa belonging to *Lachnoclostridium* (OTU 16, *P* = .035; OTU 45, *P* = .009; OTU 84, *P* = .004; OTU 22, *P* = .003), Ruminococcaceae (OTU 69, *P* = .012), and *Blautia* (OTU 93, *P* = .033; OTU 26 and 71, *P* = .031) (Tables S5 and S6). For SCFA levels from the first sample, patients with surgical IF had a higher proportional abundance of acetic acid and lower proportional abundances of propionic acid and valeric acid. The concentrations of total lactate, d‐lactate, and l‐lactate did not differ significantly between patients with surgical and functional IF (Table S7).

### Correlations between SCFA, lactate, and the gut microbiota

Positive correlations were observed between the proportional ratios (%) of propionic, butyric, and valeric acids and the two branched‐chain fatty acids with the relative abundance of Firmicutes, Bacteroidetes, and Actinobacteria (Figure S2). In contrast, the concentration of acetic acid was negatively correlated with the relative abundance of these phyla. Correlations of opposite direction to those reported above were observed between the SCFA and iso‐valeric acid and iso‐butyric acid and the relative abundance of Proteobacteria (Figure S2).

### Microbiota composition and its diet‐related metabolic activity in relation with disease characteristics

#### Nutrition characteristics

Using a mixed model to account for the repeated‐measure design, we associated microbiota measures with disease characteristics. In all sample analyses, a higher energy intake from PN was associated with a lower Shannon diversity (Table 3). Duration of PN was not associated with metrics of alpha diversity, concentrations of SCFA, lactate, total bacterial load, or OTU relative abundances (Table S8, S9, S10, and S11). With regard to microbiota community structure, the duration of PN and percentage of energy intake from PN explained 5.5% and 6.3% of the variation in microbiota community structure (*P* = .005) (Table S12). Interestingly, the amount of fiber (g/kg) intake was positively associated with the concentration of acetic, propionic, and iso‐valeric acid (Table S8). Fiber intake accounted for 4.8% (*P* = .011) of the variance in microbiota community structure.

**Table 3:** Relationships between intestinal failure patients’ disease characteristics and metrics of alpha diversity

**Characteristics**	**Shannon diversity**		**Chao richness**		**Pielou's evenness**		**Rarefied richness**	
	**Beta coefficient**	***P*‐value unadjusted/adjusted**	**Beta coefficient**	***P*‐value unadjusted/adjusted**	**Beta coefficient**	***P*‐value unadjusted/adjusted**	**Beta coefficient**	***P*‐value unadjusted/adjusted**
**Nutrition**								
Duration of PN, years	−0.83	.230/.688	−4.12	.306/.729	0.01	.729/.807	−3.99	.119/.618
Type of nutrition								
PN only	NA	<.001/.001	NA	.021/.079	NA	.213/.300	NA	.001/.016
PN + tube feeding	0.03		−45.4		0.10		−28.2
PN + oral nutrition ± tube feeding	1.86		−4.39		0.06		−2.26	
Tube feeding/oral nutrition	17.6		40.44		0.17		44.1	
PN dependency, % of total energy intake	−0.13	.001/.026	−0.33	.310/.418	−0.001	.087/.180	−0.26	.164/.268
Energy from PN by REE, %	−8.13	.002/.047	−24.7	.192/.298	−0.10	.073/.227	−18.6	.122/.265
Oral nutrition, no/yes	6.10	.021/.318	37.6	.046/.427	0.03	.595/.768	27.7	.018/.318
Fiber intake, g/kg	20.1	.017/.135	−85.3	.083/.251	0.35	.024/.768	−18.3	.616/.318
Tube feeding, no/yes	1.49	.614/.732	−39.4	.045/.349	0.09	.133/.474	−18.1	.153/.474
Tube feeding type								
Polymeric	NA	.009/.103	NA	.015/.103	NA	.160/.552	NA	.017/.103
Semielemental	−8.89		10.1		−0.22		−1.36	
Elemental	8.07		96.3		−0.01		61.1	
Mode of tube feeding								
Continuous	NA	.806/.957	NA	.290/.786	NA	.919/.957	NA	.242/.786
Bolus	−1.97		−29.7		−0.03		−24.0	
Both	−3.16		−35.1		0.01		−21.7	
**Gastrointestinal characteristics**								
* *Whole bowel in situ, no/yes	7.47	.052/.146	70.0	<.001/.002	0.09	.282/.514	43.6	<.001/.003
* *Remnant small‐bowel length, cm	−0.01	.970/.989	−0.01	.956/.989	0.01	.516/.842	−0.28	.041/.605
* *Ileocecal valve in situ, no/yes	8.86	.016/.098	55.7	.013/.098	0.05	.565/.802	40.5	.005/.051
* *Partial or total colectomy, no/yes	−4.76	.194/.460	−45.0	.014/.224	−0.05	.537/.716	−31.3	.008/.224
**Growth**								
* *BMI SDS	−0.49	.731/.872	−17.2	.157/.852	0.02	.463/.852	−5.20	.507/.852
* *Height SDS <−2, no/yes	19.2	.004/.044	111	.008/.059	0.13	.370/.704	76.9	.003/.044
* *Growing outside target height range, no/yes	14.6	.018/.251	67.7	.084/.432	0.12	.409/.797	54.8	.024/.251
**Medication use, no/yes**								
* *Proton pump inhibitor	−8.81	.003/.024	−0.21	.991/.991	−0.13	.029/.089	−14.4	.261/.427
* *Motility agents	−7.40	.042/.087	−14.5	.501/.648	−0.23	.001/.012	−19.7	.172/.267
* *Cholestyramine	16.2	<.001/<.001	15.3	.542/.647	0.02	.772/.825	39.8	.064/.241
* *Ursochol	−21.7	<.001/.001	−105	.011/.080	−0.06	.543/.836	−76.5	.014/.080
* *Antibiotics at sample^a^	−1.51	.566/.784	1.89	.913/.967	−0.10	.046/.095	2.04	.860/.967
* *Antibiotics between samples	−4.53	.040/.089	5.62	.749/.844	−0.10	.031/.073	−4.61	.688/.844
**Line sepsis, no/yes^b^**	−6.98	.028/.173	−27.2	.260/.537	−0.05	.435/.562	−12.8	.413/.562

*Note*: A positive beta coefficient means that the two variables are positively associated; a negative beta coefficient means that they are negatively associated. For categorical variables, the beta coefficients of all categories relative to the first category are mentioned; positive coefficients mean that they are more positively associated with higher values of the response variable than the first category.

Abbreviations: BMI, body mass index; NA, not applicable; PN, parenteral nutrition; REE, resting energy expenditure; SDS, standard deviation score.

^a^Due to bacterial overgrowth, line sepsis, or another cause.

^b^With a range of 2 months before and 2 months after sample collection.

Disease characteristics that are associated with OTU relative abundances are presented in Table S10. OTUs belonging to Bacteroides were positively related to the presence of oral nutrition (OTU 13, *P* = .015; and OTU 28, *P* = .032) as well as the amount of fiber intake (OTU 99, *P* < .001; OTU 145 and 32, *P* = .001; OTU 13, *P* = .008).

#### Gastrointestinal anatomy

Having the entire small bowel in situ was positively associated with the Chao1 and rarefied OTU richness. Likewise, an ileocecal valve in situ was positively associated with the Shannon diversity, Chao1, and rarefied OTU richness but not with lactate levels or total bacterial load. Gastrointestinal anatomy did not significantly explain the variance in microbiota community structure.

#### Medication

Use of antibiotics at or between sample collection was negatively associated with the absolute concentration of propionic, iso‐butyric, butyric, and valeric acid levels and positively associated with the concentration of acetic acid (Table S7).

#### Changes in the gut microbiota and SCFA during weaning from PN

**Figure 4:**
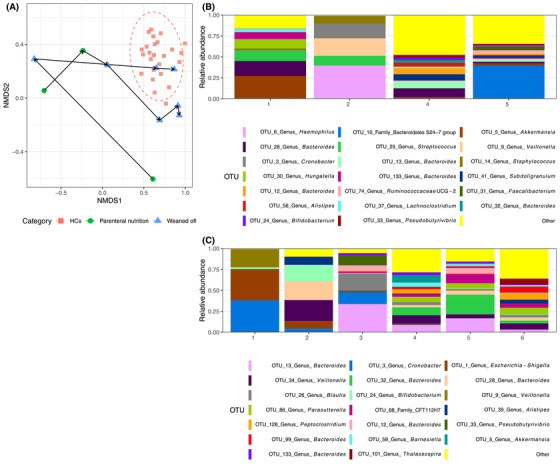
(A) Nonmetric multidimensional scaling (NMDS) of bacterial operational taxonomic unit (OTU) community structures for the two patients who weaned from parenteral nutrition during the study period (each line represents samples of one patient over time during weaning). (B–C) Microbiota taxonomic composition of two patients who were able to wean from parenteral nutrition. (B) Patient with functional intestinal failure (IF), receiving parenteral nutrition at the first sample and weaned from parenteral nutrition afterwards. (C) Patient with surgical IF, receiving parenteral nutrition at the first two samples and weaned from parenteral nutrition afterwards.

The microbiota composition of patients with IF presented a significant degree of interindividual and intraindividual variation during the observational period (Figure S3). When looking at the two patients (13%) who weaned from PN during the study period after a total PN duration of 1.2 and 2.0 years, respectively, their microbiota community structure moved closer to that of healthy controls (Figure 4) and became more diverse, with blooming of OTUs belonging to Bacteroidetes and Bifidobacteria. Although we lacked statistical power to formally confirm that, the Chao1 richness estimate of these two patients appeared to be lower at first sample collection than that of the rest of the patients who had not weaned yet during the observational period of the study. The amount of energy provided from enteral nutrition (EN) was a strong modifier of microbiota composition as well as the profile of SCFAs produced. Patients with an EN consumption >75% of daily energy intake presented a microbiota composition and diet‐related metabolic activity fairly similar to those with a healthy status (Figure 1).

## DISCUSSION

This study characterized the fecal microbiota of pediatric IF over time and related it with disease characteristics. IF patients presented distinct features of microbial dysbiosis, in terms of both compositional shifts and diet‐related functionality.^5^, ^6^, ^7^, ^25^, ^26^ Bacterial diversity and richness, presumptive markers of optimal gut health, were markedly reduced in IF, and the microbial structure was distinct from that of healthy controls. We also observed different microbial signatures between patients with SBS and functional IF and that microbial dysbiosis appeared to resolve during gut adaptation.

IF patients had a major increase in the abundance of Proteobacteria, although this phylum normally represents a very small fraction of the healthy human gut microbiota.^5^, ^6^, ^7^, ^9^ Several species belonging to Proteobacteria are opportunistic pathogens, including certain strains of *Escherichia coli*, *Klebsiella*, and *Cronobacter*, but the clinical significance of these alterations in IF disease outcomes is still uncertain. It is possible that increase of these taxa and their metabolites, in conjunction with a compromised gut barrier function and suppression of beneficial species, in patients with IF may increase translocation of inflammatory bacterial components such as lipopolysaccharides. This, in turn, may provoke an immune response and subclinical inflammation, potentially affecting disease outcomes and prognosis.^27^

In this study, we observed marked differences in the microbiota community structure between patients with surgical IF and those who have IF caused by loss of gut function but have their whole gut in situ. Functional IF patients had a microbial community structure closer to that of healthy controls than to that of surgical IF patients. Moreover, patients with functional IF had a lower abundance of taxa belonging to *Lactobacillus* and *Cronobacter* and lower concentrations of lactate. Changes in normal gastrointestinal anatomy and physiology are determining factors of microbial composition. Extensive small‐bowel resection alters intestinal environment, including lowering luminal pH, increasing oxygen concentration, and disrupting the enterohepatic circulation of bile acids.^28^, ^29^, ^30^ Other factors that might play a role are rapid transit time and the large amount of undigested nutrients presented to the colon for bacterial use.^31^ All of this may lead to the proliferation of aerobes at the expense of anaerobic bacteria. However, in our study, we found that the duration of PN, percentage of energy intake from PN, and fiber intake explained most of the variance in microbiota community structure, whereas gastrointestinal anatomy did not. The lack of fermentable substrate necessary for anaerobic bacterial growth, such as fiber and resistance starch, most likely also explains the profound decline in fiber‐fermenting species belonging to Firmicutes, Actinobacteria, and Bacteroidetes and the parallel effects on their metabolic products. This finding is in contrast with a previous study that included infants with SBS and showed only differences in fecal acetic acid concentration.^7^

In accordance with previous studies, we showed that the higher amount of EN patients received, the lower the abundance of Proteobacteria was.^5^, ^6^, ^25^, ^32^ Interestingly, when we looked at the microbiota of patients whose gut adapted, diversity increased and their overall microbial community structure moved closer to that of the healthy controls, strongly supporting the extensive modifying effect and dependency of gut microbiota on the host's diet.

The dominance of lactic acid–producing bacteria and the decreased abundance of lactic acid utilizers^33^, ^34^, ^35^ resulted in accumulation of both d‐lactate and l‐lactate, as we observed here. Although not significant, we did observe higher levels of lactate in surgical IF patients, in agreement with the fact that they also had a higher relative abundance of *Lactobacillus* than functional IF patients had. In contrast to previous studies,^6^, ^7^ we were not able to relate this to d‐lactic acidosis, as none of the included patients in the current study developed this condition.

In the two patients whose gut adapted and were weaned from PN, their microbiota structure clustered within or was close to that of the healthy status. Future research should explore whether these microbial changes precede or follow gut adaptation. It is possible that early shifts in microbial signals during gut adaptation may offer opportunities to develop new biomarkers to direct clinical practice on the optimal time of transition from PN to EN. Potential candidates comprise SCFAs, particularly butyric acid, which presented the largest suppression during PN but also the largest recovery during gut adaptation. The influence of dysbiosis on clinical outcomes—including small‐bowel bacterial overgrowth, d‐lactic acidosis, and PN‐associated liver disease—should also be explored in future prospective research. Currently, there are no clear guidelines to state whether children with IF should receiver fiber supplementation or how much fiber should be used, but the findings of this study support this practice.^36^ Future studies should therefore evaluate responses to fiber therapy and also focus on type and dose of these fiber substrates.^35^

The strengths of this study are the serial sample collection for each patient and the correlation analysis we performed with prospectively collected clinical metadata. However, one of the limitations is the modest sample size, including the fact that only two patients were weaned during this study, and we were therefore unable to perform formal statistical analysis. Likewise, some of the interindividual variation in the microbiome structure of patients with IF might be attributed to the heterogeneity of their primary pathology leading to IF. This study included a single patient with enterostomy, but it is interesting that this patient's microbiome diversity and structure did not differ substantially from that of the rest of their group. Although we aimed to collect samples every 3 months from study enrollment, for some patients it was not possible, owing to logistic reasons. Future multicenter studies are next required to corroborate and build upon the findings of the current research. Another limitation was the fact that most patients received antibiotics within 2 months prior to sample collection, as well as antibiotics’ influence on gut microbiota.^37^, ^38^, ^39^ However, this reflects “real‐life” clinical practice and the population typically treated by IF teams. In addition, information about fiber intake from habitual diet was not recorded, although the amount of table food consumed was negligible in most patients. Lastly, results were based on next‐generation sequencing, and as such, data are presented as relative abundances. Future studies should validate whether changes in the abundance of important bacteria correspond to changes in their absolute concentrations using targeted qPCR.

In summary, we have observed pronounced alterations in the composition and diet‐related metabolic activity of the fecal microbiota, not only between pediatric IF patients and healthy controls but also within different subtypes of IF. These findings may prompt further research to evaluate the use of microbial signatures as prognostic markers of disease outcomes or a therapeutic target during the process of gut adaptation.

## CONFLICT OF INTEREST

Konstantinos Gerasimidis received research grants, honoraria, and consultancy fees from Nestlé Health Sciences and Nutricia‐Danone. All other authors declare no conflict of interest.

## AUTHOR CONTRIBUTIONS

Esther G. Neelis, Barbara A. E. de Koning, Jessie M. Hulst, Edmond H. H. M. Rings, René M. H. Wijnen, Ben Nichols, and Konstantinos Gerasimidis contributed to the conception and design of the study. Esther G. Neelis, Barbara A. E. de Koning, Rodanthi Papadopoulou, Caroline Kerbiriou, Ben Nichols, and Konstantinos Gerasimidis contributed to the acquisition of data. Esther G. Neelis, Barbara A. E. de Koning, Jessie M. Hulst, Rodanthi Papadopoulou, Caroline Kerbiriou, Ben Nichols, and Konstantinos Gerasimidis contributed to the analysis and interpretation of the data. Esther G. Neelis, Barbara A. E. de Koning, Ben Nichols, and Konstantinos Gerasimidis drafted the article. Rodanthi Papadopoulou, Caroline Kerbiriou, Jessie M. Hulst, Edmond H. H. M. Rings, and René M. H. Wijnen revised the manuscript critically. All authors read and approved the final version of the manuscript.

## Supporting information

Supplementary Table 1. Differences in relative abundance between IF patients and healthy controls for all samples at the OTU level
Supplementary Table 2. Differences in relative abundance between IF patients and healthy controls for all samples at the family level
Supplementary Table 3. Concentration of SCFA and lactate for IF patients and healthy controls at first sample collection per gram of wet feces
Supplementary Table 4. Fecal water content, concentration of SCFA, lactate, and number of 16S rRNA gene copies for IF patients and healthy controls at last sample
Supplementary Table 5. Differences in relative abundance between surgical IF and functional IF patients for all samples at the OTU level
Supplementary Table 6. Differences in relative abundance between functional and surgical IF patients for all samples at the family level
Supplementary Table 7. Concentration of SCFA and lactate for surgical IF and functional IF patients at first sample collection per gram of dry feces
Supplementary Table 8. General linear mixed model for clinical variables and percentage of short‐chain fatty acids
Supplementary Table 9. General linear mixed model for clinical variables and absolute values of short‐chain fatty acids
Supplementary Table 10. General linear mixed model for clinical variables and lactate and number of 16S rRNA gene copies
Supplementary Table 11. Clinical variables associated with OTUs
Supplementary Table 12. Permutation ANOVA for the relation between disease characteristics and microbiota community structure (beta diversity), taking into account multiple samples per patient
Supplementary Figure 1. Taxonomic composition of microbiota of healthy controls (left) and pediatric IF patients (right) at the family level at first sample (above) and for all samples (below)
Supplementary Figure 2. Heat map of correlations between the main six phyla of the gut microbiota and short‐chain fatty acids and d‐lactate (both per gram of dry feces)
