# Supplementary material for: Gut microbiota and its diet‐related activity in children with intestinal failure receiving long‐term parenteral nutrition
Source: JPEN J Parenter Enteral Nutr. 2021 Aug 25;46(3):693–708. doi: 10.1002/jpen.2188 (PMC9255855; doi:10.1002/jpen.2188)
Supplement: Supplementary file 1 — Supplementary Table 1. Differences in relative abundance between IF patients and healthy controls for all samples at the OTU level Supplementary Table 2. Differences in relative abundance between IF patients and healthy controls for all samples at the family level Supplementary Table 3. Concentration of SCFA and lactate for IF patients and healthy controls at first sample collection per gram of wet feces Supplementary Table 4. Fecal water content, concentration of SCFA, lactate, and number of 16S rRNA gene copies for IF patients and healthy controls at last sample Supplementary Table 5. Differences in relative abundance between surgical IF and functional IF patients for all samples at the OTU level Supplementary Table 6. Differences in relative abundance between functional and surgical IF patients for all samples at the family level Supplementary Table 7. Concentration of SCFA and lactate for surgical IF and functional IF patients at first sample collection per gram of dry feces Supplementary Table 8. General linear mixed model for clinical variables and percentage of short‐chain fatty acids Supplementary Table 9. General linear mixed model for clinical variables and absolute values of short‐chain fatty acids Supplementary Table 10. General linear mixed model for clinical variables and lactate and number of 16S rRNA gene copies Supplementary Table 11. Clinical variables associated with OTUs Supplementary Table 12. Permutation ANOVA for the relation between disease characteristics and microbiota community structure (beta diversity), taking into account multiple samples per patient Supplementary Figure 1. Taxonomic composition of microbiota of healthy controls (left) and pediatric IF patients (right) at the family level at first sample (above) and for all samples (below) Supplementary Figure 2. Heat map of correlations between the main six phyla of the gut microbiota and short‐chain fatty acids and d‐lactate (both per gram of dry feces) [file JPEN-46-693-s001.pdf]

## Supplementary methods

### Lactate

Fifty mg of freeze dried feces was suspended in 800 µl distilled water, and the suspension was incubated at 65° C during 20 minutes. Impurities were removed and fecal lactate was determined in extracts according to the manufacturer's instruction.

### Short-chain fatty acids/branch-chain fatty acids

SCFA/BCFA were measured in diethyl ether extracts by gas chromatography using a TRACE™ 7890A flame ionization detector (FID) gas chromatograph and a Zerbon ZB-Was capillary column were used. Nitrogen was used as the carrier gas. Internal standard and orthophosphoric acid (16M) were added to 75 mg of freeze-dried samples. The internal standard was used to correct the losses that occur during handling, extraction and quantification of the samples while the orthophosphoric acid was used to reduce the dissociation constant of the acids, so that it separates from the rest of the sample into the ether phase. The mixture was extracted three times with 1.5 ml of diethyl ether, centrifuged and the supernatant collected. External standards were used for calibration (185.8 µmol/l acetic, 144.4 µmol/l propionic, 97.3 µmol/l isobutyric, 114.1 µmol/l butyric, 87 µmol/l isovaleric, 83.4 µmol/l valeric, 52.6 µmol/l isocaproic, 76.5 µmol/l caproic, 65.7 µmol/l heptanoic, 53.1 µmol/l octanoic, Sigma-Aldrich, UK). All samples coming from the same patient were analyzed at the same run to minimize intra-assay error. An external quality control sample of a mixture of feces samples was run at the beginning of the run and at the end to assure the repeatability of the assay and the intra-assay between the runs.

**DNA extraction**

Fifty mg of freeze dried feces was suspended in 250 µl of 4M guanidine thiocyanate (Sigma-Aldrich, UK), 0.1M Tris-Cl (pH 7.5) and 40 µl of N-lauroyl sarcosine 10% (Sigma-Aldrich, UK) buffer. After that, 500 µl 5% N-lauroylsarcosine in 0.1 M phosphate buffer (pH 8) was added, and the sample was incubated at 70˚C for 1 hour. Sterile zirconium beads (0.1 mm, BioSpec, USA) were added and the bacterial cells were lysed three times for 60 seconds with a bead beater. Polyvinylpyrrolidone (Sigma-Aldrich, UK) (15 mg) was added to the sample, which was vortexed and centrifuged for 3 min at 15,000 g at 4˚C. The supernatant was collected and the pellet was washed three times with 400 µl TENP (50 mM Tris (pH 8), 20 mM EDTA (pH 8), 100 mM NaCl, 1% polyvinylpolypyrrolidone) and centrifuged for 3 min at 15,000 g and 4˚C each time. Nucleic acids were precipitated by the addition of isopropanol v/v for 10 m room temperature, and centrifuged 5 min at 15,000 g. The pellet was suspended in 225 µl 0.1M phosphate buffer (pH 8) and 25 µl 5M potassium acetate (Sigma-Aldrich, UK) and the sample was left in a fridge overnight. The next day, samples were centrifuged at 15,000 g and 4˚C for 30 minutes, and 5 µl RNAse (10 mg/ml) was added to the supernatant (Sigma-Aldrich, UK). The mixture was incubated at 37˚C for 45 min. SDS 10% (25 µl) and proteinase K (12.5 µl) were added and this mixture was incubated at 45˚C for 2 hours. Afterwards, the DNA was precipitated by the addition of 54 µl sodium acetate (Sigma-Aldrich, UK) and 1 ml of 100% ethanol stored at -20˚C. DNA was recovered after centrifugation at 15,000 g for 15 minutes, washing with 70% ethanol (stored at -20˚C), dried, and suspended in Tris-EDTA buffer.

## Bioinformatics

Microbiota composition was analyzed using operational taxonomic units (OTUs) obtained from the 16S rRNA sequencing data and clustered at a level of 97% similarity. OTUs were generated from the raw data using a modified version of the VSEARCH pipeline (https://github.com/torognes/vsearch/wiki/VSEARCH-pipeline) (1). The paired fastq files were merged together and quality filtering was performed with a fastq_maxee (maximum expected error value for merged sequences) parameter of 0.5. Sequences longer than 275bp and shorter than 225bp were filtered out. The files were then combined, dereplicated, and all singleton sequences were removed. Sequences were preclustered at 98%, and chimeras were identified and removed from the dataset using the VSEARCH implementation of the UCHIME de-novo algorithm (2). A secondary chimera detection and removal step was carried out, this time using the UCHIME reference-based method and the 'Gold' ChimeraSlayer reference dataset (3). OTUs were then generated by clustering the remaining sequences at 97%. OTUs were taxonomically classified to genus level using the assignTaxonomy function in the dada2 R package (4).

## Data analysis and statistics

For microbiota analysis, non-metric multidimensional scaling (NMDS) ordination was carried out using the phyloseq package in R (5) and permutation ANOVA was performed using the adonis function in the R vegan package (6). These methods used Bray-Curtis distance matrices as input.

Generalized linear mixed models were used to identify relationships between disease characteristics and microbial diversity measures; each model was generated using one variable of interest and the subject's age as explanatory variables with the subject ID

included as a random effect. The general linear mixed model analysis was carried out using the lme4 package in R (7). Correlation tests were performed using the cor.test function in R for unpaired data and using the rmcorr function when analyzing repeated measures. Statistics were performed using SPSS version 21 (SPSS, IBM, Armonk) and R version 3.4.3.

**References**


1. Rognes T, Flouri T, Nichols B, Quince C, Mahe F. VSEARCH: a versatile open source tool for metagenomics. *PeerJ.* 2016;4:e2584.
2. Edgar RC, Haas BJ, Clemente JC, Quince C, Knight R. UCHIME improves sensitivity and speed of chimera detection. *Bioinformatics.* Aug 15 2011;27(16):2194-200.
3. Haas BJ, Gevers D, Earl AM, Feldgarden M, Ward DV, Giannoukos G, Ciulla D, Tabbaa D, Highlander SK, Sodergren E, et al. Chimeric 16S rRNA sequence formation and detection in Sanger and 454-pyrosequenced PCR amplicons. *Genome research.* Mar 2011;21(3):494-504.
4. Callahan BJ, McMurdie PJ, Rosen MJ, Han AW, Johnson AJ, Holmes SP. DADA2: High-resolution sample inference from Illumina amplicon data. *Nature methods.* Jul 2016;13(7):581-3.
5. McMurdie PJ, Holmes S. phyloseq: an R package for reproducible interactive analysis and graphics of microbiome census data. *PLoS One.* 2013;8(4):e61217.
6. Oksanen J, Guillaume Blanchet F, Friendly M, Legendre P, McGlinn D, Minchin PR, O'Hara RB, Simpson G, P. S, Stevens M, et al. vegan: Community Ecology Package. R package version 2.5-3. 2018; https://CRAN.R-project.org/package=vegan.
7. Bates D, Mächler M, Bolker B, Walker S. Fitting Linear Mixed-Effects Models Using lme4. *Journal of Statistical Software.* 2015;67(1):1-48.

**Supplementary Table 1.** Differences in relative abundance between IF patients and healthy controls for all samples at OTU level

| OTU | p-value | adjusted p-value | Differences in mean log relative abundance |
|---|---|---|---|
| OTU_114_Genus_Faecalibacterium | 1.24E-21 | 2.48E-19 | -5.376 |
| OTU_60_Genus_Anaerostipes | 1.61E-15 | 1.61E-13 | -4.834 |
| OTU_183_Genus_[Eubacterium] ventriosum group | 1.62E-13 | 1.08E-11 | -3.921 |
| OTU_122_Genus_Lachnospiraceae ND3007 group | 1.28E-12 | 6.38E-11 | -4.533 |
| OTU_123_Genus_Erysipelotrichaceae UCG-003 | 2.47E-12 | 9.88E-11 | -3.932 |
| OTU_106_Genus_[Eubacterium] hallii group | 9.19E-12 | 3.06E-10 | -3.839 |
| OTU_31_Genus_Faecalibacterium | 7.80E-11 | 2.23E-09 | -5.758 |
| OTU_83_Genus_Ruminococcus 1 | 2.96E-10 | 7.41E-09 | -4.240 |
| OTU_108_Genus_Fusicatenibacter | 5.15E-10 | 1.14E-08 | -3.521 |
| OTU_67_Genus_Collinsella | 6.07E-10 | 1.21E-08 | -4.688 |
| OTU_48_Genus_Blautia | 1.21E-09 | 1.88E-08 | -3.414 |
| OTU_154_Genus_Incertae Sedis | 1.26E-09 | 1.88E-08 | -3.659 |
| OTU_57_Genus_Subdoligranulum | 1.28E-09 | 1.88E-08 | -4.403 |
| OTU_129_Genus_Lachnospiraceae NC2004 group | 1.32E-09 | 1.88E-08 | -3.642 |
| OTU_98_Genus_Lachnospiraceae NK4A136 group | 1.56E-09 | 2.06E-08 | -4.216 |
| OTU_204_Genus_Ruminiclostridium | 1.65E-09 | 2.06E-08 | -3.983 |
| OTU_63_Genus_Lachnoclostridium | 2.58E-09 | 3.03E-08 | -3.677 |
| OTU_55_Genus_Blautia | 6.57E-09 | 7.02E-08 | -4.166 |
| OTU_347_Genus_Lachnoclostridium | 6.66E-09 | 7.02E-08 | -2.972 |
| OTU_179_Genus_Coprococcus 1 | 9.14E-09 | 9.14E-08 | -3.053 |
| OTU_191_Genus_Lachnospiraceae UCG-008 | 1.05E-08 | 9.95E-08 | -3.536 |
| OTU_220_Family_Lachnospiraceae | 1.21E-08 | 1.10E-07 | -4.571 |
| OTU_167_Genus_Ruminococcus 1 | 3.31E-08 | 2.81E-07 | -3.704 |
| OTU_140_Genus_Ruminococcaceae NK4A214 group | 3.37E-08 | 2.81E-07 | -3.843 |
| OTU_251_Genus_Ruminiclostridium 9 | 3.62E-08 | 2.89E-07 | -2.591 |
| OTU_97_Genus_Dorea | 5.01E-08 | 3.86E-07 | -3.643 |
| OTU_86_Genus_Parasutterella | 7.56E-08 | 5.60E-07 | -3.617 |
| OTU_200_Genus_Roseburia | 1.09E-07 | 7.78E-07 | -2.959 |
| OTU_33_Genus_Pseudobutyrivibrio | 1.31E-07 | 9.05E-07 | -3.859 |
| OTU_89_Genus_Lachnospira | 1.46E-07 | 9.75E-07 | -3.909 |

| | | | |
|---|---|---|---|
| OTU_124_Genus_[Ruminococcus] gauvreauii group | 2.35E-07 | 1.52E-06 | -3.283 |
| OTU_197_Genus_Enterorhabdus | 2.48E-07 | 1.55E-06 | -2.429 |
| OTU_228_Genus_Lachnoclostridium | 2.63E-07 | 1.59E-06 | -3.166 |
| OTU_121_Genus_Dorea | 3.94E-07 | 2.32E-06 | -3.683 |
| OTU_113_Genus_Lachnoclostridium | 4.23E-07 | 2.42E-06 | -4.277 |
| OTU_670_Genus_Alistipes | 4.40E-07 | 2.44E-06 | -2.529 |
| OTU_290_Genus_Collinsella | 4.78E-07 | 2.58E-06 | -2.608 |
| OTU_41_Genus_Subdoligranulum | 5.91E-07 | 3.11E-06 | -3.274 |
| OTU_162_Genus_[Eubacterium] hallii group | 9.25E-07 | 4.74E-06 | -3.759 |
| OTU_85_Genus_Lachnospiraceae NK4A136 group | 1.07E-06 | 5.35E-06 | -4.381 |
| OTU_65_Genus_Ruminococcaceae UCG-002 | 1.32E-06 | 6.43E-06 | -3.808 |
| OTU_297_Genus_Lachnospiraceae UCG-001 | 1.68E-06 | 8.02E-06 | -2.685 |
| OTU_59_Genus_Barnesiella | 1.74E-06 | 8.07E-06 | -2.904 |
| OTU_153_Genus_Lachnospiraceae FCS020 group | 2.25E-06 | 1.02E-05 | -2.686 |
| OTU_73_Genus_Coprococcus 3 | 3.22E-06 | 1.43E-05 | -3.335 |
| OTU_943_Class_Bacteroidetes VC2.1 Bac22 | 3.84E-06 | 1.67E-05 | -2.422 |
| OTU_42_Genus_Ruminococcus 2 | 4.13E-06 | 1.76E-05 | -4.088 |
| OTU_286_Family_Clostridiales vadinBB60 group | 5.17E-06 | 2.15E-05 | -2.397 |
| OTU_272_Genus_Ruminococcaceae UCG-005 | 5.32E-06 | 2.17E-05 | -2.276 |
| OTU_102_Genus_Odoribacter | 5.83E-06 | 2.33E-05 | -3.728 |
| OTU_82_Genus_Bacteroides | 7.78E-06 | 3.05E-05 | -3.502 |
| OTU_78_Genus_Blautia | 8.43E-06 | 3.24E-05 | -2.958 |
| OTU_77_Genus_Ruminococcaceae UCG-002 | 9.43E-06 | 3.56E-05 | -2.811 |
| OTU_74_Genus_Ruminococcaceae UCG-002 | 1.00E-05 | 3.68E-05 | -3.636 |
| OTU_159_Class_Bacteroidetes VC2.1 Bac22 | 1.01E-05 | 3.68E-05 | -2.458 |
| OTU_40_Genus_Phascolarctobacterium | 1.07E-05 | 3.81E-05 | -3.787 |
| OTU_262_Genus_Ruminococcus 1 | 1.15E-05 | 4.02E-05 | -2.474 |
| OTU_120_Genus_Ruminiclostridium 6 | 1.32E-05 | 4.54E-05 | -2.262 |
| OTU_116_Genus_Ruminococcaceae UCG-005 | 1.53E-05 | 5.20E-05 | -2.697 |
| OTU_13_Genus_Bacteroides | 1.62E-05 | 5.41E-05 | -3.985 |
| OTU_252_Genus_Gordonibacter | 2.46E-05 | 8.07E-05 | -2.392 |
| OTU_119_Genus_Ruminococcus 1 | 2.53E-05 | 8.07E-05 | -2.973 |
| OTU_146_Genus_Intestinibacter | 2.54E-05 | 8.07E-05 | -2.337 |

| | | | |
|---|---|---|---|
| OTU_239_Genus_Terrisporobacter | 2.96E-05 | 9.25E-05 | -2.204 |
| OTU_185_Genus_Senegalimassilia | 3.28E-05 | 1.01E-04 | -2.386 |
| OTU_110_Genus_Alistipes | 3.38E-05 | 1.02E-04 | -2.868 |
| OTU_194_Genus_Butyricimonas | 3.72E-05 | 1.11E-04 | -2.619 |
| OTU_201_Family_Lachnospiraceae | 4.07E-05 | 1.20E-04 | -2.742 |
| OTU_142_Genus_[Eubacterium] coprostanoligenes group | 4.34E-05 | 1.26E-04 | -2.874 |
| OTU_1049_Genus_Lachnospiraceae NC2004 group | 4.57E-05 | 1.30E-04 | -2.221 |
| OTU_75_Family_Ruminococcaceae | 4.62E-05 | 1.30E-04 | -2.726 |
| OTU_26_Genus_Blautia | 5.64E-05 | 1.57E-04 | -3.538 |
| OTU_327_Genus_Lachnospiraceae NC2004 group | 5.88E-05 | 1.59E-04 | -2.168 |
| OTU_100_Genus_Coprococcus 2 | 5.94E-05 | 1.59E-04 | -2.666 |
| OTU_189_Genus_Ruminococcaceae UCG-013 | 5.94E-05 | 1.59E-04 | -1.963 |
| OTU_221_Genus_Alistipes | 6.08E-05 | 1.60E-04 | -1.906 |
| OTU_71_Genus_Blautia | 6.46E-05 | 1.68E-04 | -3.997 |
| OTU_324_Genus_Ruminiclostridium 9 | 6.83E-05 | 1.75E-04 | -2.231 |
| OTU_14_Genus_Staphylococcus | 8.67E-05 | 2.19E-04 | 4.025 |
| OTU_145_Genus_Bacteroides | 9.58E-05 | 2.39E-04 | -2.573 |
| OTU_68_Genus_Ruminococcaceae UCG-005 | 1.24E-04 | 3.06E-04 | -3.264 |
| OTU_203_Genus_Ruminiclostridium 6 | 1.63E-04 | 3.98E-04 | -2.068 |
| OTU_173_Genus_Lachnospiraceae NK4A136 group | 1.70E-04 | 4.10E-04 | -1.718 |
| OTU_205_Genus_Alistipes | 1.80E-04 | 4.28E-04 | -2.029 |
| OTU_125_Genus_Ruminococcaceae UCG-003 | 2.15E-04 | 5.06E-04 | -2.768 |
| OTU_64_Genus_Ruminococcus 1 | 2.90E-04 | 6.74E-04 | -3.283 |
| OTU_49_Genus_Roseburia | 2.95E-04 | 6.77E-04 | -3.480 |
| OTU_105_Genus_Lachnospiraceae UCG-003 | 3.91E-04 | 8.83E-04 | -2.160 |
| OTU_152_Genus_Christensenellaceae R-7 group | 3.93E-04 | 8.83E-04 | -2.732 |
| OTU_32_Genus_Bacteroides | 4.22E-04 | 9.38E-04 | -2.767 |
| OTU_180_Family_Coriobacteriaceae | 4.64E-04 | 1.02E-03 | -1.553 |
| OTU_24_Genus_Bifidobacterium | 5.24E-04 | 1.14E-03 | -2.802 |
| OTU_56_Genus_Alistipes | 6.25E-04 | 1.33E-03 | -3.169 |
| OTU_259_Genus_Ruminococcus 1 | 6.26E-04 | 1.33E-03 | -1.943 |
| OTU_235_Genus_Lachnospiraceae NK4A136 group | 6.47E-04 | 1.36E-03 | -2.154 |
| OTU_3_Genus_Cronobacter | 6.96E-04 | 1.45E-03 | 3.795 |

| | | | |
|---|---|---|---|
| OTU_109_Genus_Anaerostipes | 7.27E-04 | 1.50E-03 | -1.813 |
| OTU_70_Genus_Bacteroides | 8.72E-04 | 1.78E-03 | -2.301 |
| OTU_93_Genus_Blautia | 9.98E-04 | 2.02E-03 | -2.403 |
| OTU_186_Genus_Ruminococcaceae UCG-010 | 1.05E-03 | 2.10E-03 | -1.729 |
| OTU_229_Genus_Clostridium sensu stricto 1 | 1.32E-03 | 2.61E-03 | -1.674 |
| OTU_50_Genus_Christensenellaceae R-7 group | 1.34E-03 | 2.63E-03 | -2.491 |
| OTU_23_Genus_Alistipes | 1.67E-03 | 3.24E-03 | -3.356 |
| OTU_215_Genus_Erysipelatoclostridium | 1.68E-03 | 3.24E-03 | -1.661 |
| OTU_198_Genus_Anaerotruncus | 1.77E-03 | 3.37E-03 | -1.530 |
| OTU_275_Family_Lachnospiraceae | 1.81E-03 | 3.42E-03 | -1.297 |
| OTU_169_Genus_Ruminococcus 1 | 1.87E-03 | 3.50E-03 | -1.233 |
| OTU_250_Genus_Oscillibacter | 2.43E-03 | 4.50E-03 | -1.806 |
| OTU_134_Genus_Ruminococcaceae UCG-005 | 2.49E-03 | 4.57E-03 | -1.818 |
| OTU_187_Genus_[Eubacterium] ruminantium group | 2.60E-03 | 4.72E-03 | -1.633 |
| OTU_39_Genus_Alistipes | 3.11E-03 | 5.60E-03 | -2.431 |
| OTU_28_Genus_Bacteroides | 3.20E-03 | 5.72E-03 | -2.897 |
| OTU_170_Genus_Ruminococcaceae UCG-014 | 3.58E-03 | 6.31E-03 | -1.586 |
| OTU_1_Genus_Escherichia-Shigella | 3.59E-03 | 6.31E-03 | 3.354 |
| OTU_10_Family_Bacteroidales S24-7 group | 4.34E-03 | 7.55E-03 | -2.184 |
| OTU_99_Genus_Bacteroides | 4.41E-03 | 7.56E-03 | -1.356 |
| OTU_112_Genus_Ruminiclostridium 5 | 4.43E-03 | 7.56E-03 | -2.129 |
| OTU_66_Genus_Paraprevotella | 4.64E-03 | 7.86E-03 | -1.504 |
| OTU_90_Genus_Thalassospira | 5.66E-03 | 9.51E-03 | -1.398 |
| OTU_148_Family_Ruminococcaceae | 5.89E-03 | 9.79E-03 | -1.704 |
| OTU_6_Genus_Haemophilus | 5.92E-03 | 9.79E-03 | -1.610 |
| OTU_133_Genus_Bacteroides | 6.21E-03 | 1.02E-02 | -2.676 |
| OTU_104_Genus_Ruminiclostridium 5 | 6.47E-03 | 1.05E-02 | -1.977 |
| OTU_132_Genus_Desulfovibrio | 8.69E-03 | 1.39E-02 | -1.789 |
| OTU_144_Genus_Ruminococcaceae UCG-004 | 8.70E-03 | 1.39E-02 | -2.046 |
| OTU_12_Genus_Bacteroides | 1.06E-02 | 1.68E-02 | -2.674 |
| OTU_25_Genus_Streptococcus | 1.09E-02 | 1.71E-02 | -2.331 |
| OTU_230_Genus_Coprobacter | 1.12E-02 | 1.75E-02 | -1.378 |
| OTU_47_Genus_Dialister | 1.16E-02 | 1.80E-02 | -1.992 |

| | | | |
|---|---|---|---|
| OTU_160_Genus_Paraprevotella | 1.33E-02 | 2.04E-02 | -1.693 |
| OTU_44_Genus_[Eubacterium] coprostanoligenes group | 1.35E-02 | 2.05E-02 | -1.540 |
| OTU_80_Family_Lachnospiraceae | 1.38E-02 | 2.10E-02 | -1.952 |
| OTU_1064_Genus_Sutterella | 1.40E-02 | 2.11E-02 | -1.577 |
| OTU_208_Genus_Ruminiclostridium | 1.52E-02 | 2.27E-02 | -1.556 |
| OTU_118_Genus_Butyrivibrio | 1.72E-02 | 2.54E-02 | -1.063 |
| OTU_168_Genus_Bacteroides | 1.86E-02 | 2.73E-02 | -0.995 |
| OTU_111_Genus_[Eubacterium] coprostanoligenes group | 1.87E-02 | 2.73E-02 | -1.624 |
| OTU_91_Genus_Sutterella | 1.89E-02 | 2.74E-02 | -2.317 |
| OTU_188_Family_Ruminococcaceae | 2.05E-02 | 2.95E-02 | -1.264 |
| OTU_255_Genus_Ruminiclostridium 5 | 2.11E-02 | 3.02E-02 | -1.419 |
| OTU_69_Family_Ruminococcaceae | 2.24E-02 | 3.17E-02 | -1.931 |
| OTU_177_Genus_Bacteroides | 2.30E-02 | 3.23E-02 | -1.207 |
| OTU_92_Genus_Bilophila | 2.42E-02 | 3.38E-02 | -1.929 |
| OTU_135_Genus_Lachnoclostridium | 2.47E-02 | 3.43E-02 | -1.478 |
| OTU_190_Family_Christensenellaceae | 2.59E-02 | 3.57E-02 | -1.033 |
| OTU_234_Genus_Coprococcus 2 | 2.82E-02 | 3.87E-02 | -1.241 |
| OTU_151_Family_Corynebacteriaceae | 2.89E-02 | 3.94E-02 | -0.773 |
| OTU_127_Genus_Prevotellaceae NK3B31 group | 3.00E-02 | 4.06E-02 | -1.016 |
| OTU_62_Genus_Parabacteroides | 3.49E-02 | 4.69E-02 | -1.754 |
| OTU_95_Genus_[Eubacterium] oxidoreducens group | 3.89E-02 | 5.19E-02 | -1.626 |
| OTU_58_Genus_Parabacteroides | 4.98E-02 | 6.60E-02 | -1.871 |
| OTU_126_Genus_Eggerthella | 5.71E-02 | 7.52E-02 | -1.398 |
| OTU_695_Genus_Bacteroides | 6.07E-02 | 7.94E-02 | -1.274 |
| OTU_81_Genus_Paraprevotella | 7.01E-02 | 9.11E-02 | -1.226 |
| OTU_163_Genus_Blautia | 7.38E-02 | 9.52E-02 | -0.726 |
| OTU_94_Genus_Clostridium sensu stricto 1 | 9.18E-02 | 1.18E-01 | -1.195 |
| OTU_87_Genus_[Eubacterium] coprostanoligenes group | 9.73E-02 | 1.24E-01 | -1.257 |
| OTU_37_Genus_Lachnoclostridium | 9.92E-02 | 1.26E-01 | -1.279 |
| OTU_107_Genus_Lachnoclostridium | 1.05E-01 | 1.33E-01 | -1.056 |
| OTU_5_Genus_Akkermansia | 1.18E-01 | 1.47E-01 | -1.416 |
| OTU_137_Genus_Lachnospiraceae UCG-008 | 1.40E-01 | 1.74E-01 | -0.863 |
| OTU_72_Genus_Phascolarctobacterium | 1.42E-01 | 1.75E-01 | -0.951 |

| | | | |
|---|---|---|---|
| OTU_27_Genus_Neisseria | 1.43E-01 | 1.76E-01 | 0.825 |
| OTU_61_Genus_Clostridium sensu stricto 1 | 1.45E-01 | 1.77E-01 | 1.093 |
| OTU_52_Genus_Peptoclostridium | 1.51E-01 | 1.83E-01 | -1.132 |
| OTU_143_Genus_Thalassospira | 1.68E-01 | 2.03E-01 | -0.697 |
| OTU_156_Genus_Ruminiclostridium 9 | 2.09E-01 | 2.51E-01 | -0.740 |
| OTU_2_Genus_Pseudomonas | 2.15E-01 | 2.56E-01 | 1.032 |
| OTU_7_Genus_Prevotella 9 | 2.33E-01 | 2.76E-01 | -0.944 |
| OTU_96_Genus_Streptococcus | 2.83E-01 | 3.33E-01 | 0.584 |
| OTU_51_Genus_Flavonifractor | 3.17E-01 | 3.70E-01 | -0.909 |
| OTU_18_Genus_Lactobacillus | 3.19E-01 | 3.70E-01 | 0.959 |
| OTU_29_Family_Lachnospiraceae | 3.52E-01 | 4.07E-01 | 0.479 |
| OTU_171_Genus_Staphylococcus | 4.27E-01 | 4.90E-01 | 0.452 |
| OTU_19_Genus_Tyzzerella 4 | 4.33E-01 | 4.95E-01 | -0.693 |
| OTU_606_Genus_Akkermansia | 4.49E-01 | 5.11E-01 | -0.514 |
| OTU_8_Genus_Morganella | 4.73E-01 | 5.34E-01 | 0.555 |
| OTU_150_Family_Veillonellaceae | 4.93E-01 | 5.54E-01 | 0.428 |
| OTU_22_Genus_Lachnoclostridium | 5.00E-01 | 5.59E-01 | 0.649 |
| OTU_34_Genus_Veillonella | 5.28E-01 | 5.86E-01 | 0.433 |
| OTU_225_Genus_Actinomyces | 5.40E-01 | 5.96E-01 | -0.307 |
| OTU_43_Family_Lachnospiraceae | 6.16E-01 | 6.77E-01 | -0.413 |
| OTU_9_Genus_Veillonella | 6.20E-01 | 6.78E-01 | -0.409 |
| OTU_4_Genus_Lactobacillus | 6.24E-01 | 6.78E-01 | 0.392 |
| OTU_136_Genus_Ruminiclostridium 5 | 6.67E-01 | 7.18E-01 | -0.273 |
| OTU_244_Genus_Blautia | 6.68E-01 | 7.18E-01 | -0.254 |
| OTU_20_Genus_Aeromonas | 6.74E-01 | 7.18E-01 | 0.336 |
| OTU_158_Genus_Peptoniphilus | 6.78E-01 | 7.18E-01 | -0.231 |
| OTU_30_Genus_Hungatella | 6.79E-01 | 7.18E-01 | -0.441 |
| OTU_17_Genus_Lactobacillus | 6.95E-01 | 7.31E-01 | 0.317 |
| OTU_84_Genus_Lachnoclostridium | 7.59E-01 | 7.95E-01 | -0.248 |
| OTU_212_Genus_Hafnia | 8.13E-01 | 8.46E-01 | -0.133 |
| OTU_16_Genus_Lachnoclostridium | 8.27E-01 | 8.57E-01 | 0.212 |
| OTU_139_Genus_Stenotrophomonas | 8.36E-01 | 8.62E-01 | 0.112 |
| OTU_117_Genus_Epulopiscium | 9.05E-01 | 9.28E-01 | 0.068 |

| | | | |
|---|---|---|---|
| OTU_54_Genus_Proteus | 9.34E-01 | 9.53E-01 | 0.056 |
| OTU_103_Genus_Clostridium sensu stricto 1 | 9.43E-01 | 9.57E-01 | 0.046 |
| OTU_45_Genus_Lachnoclostridium | 9.68E-01 | 9.78E-01 | 0.035 |
| OTU_182_Genus_Incertae Sedis | 9.80E-01 | 9.85E-01 | -0.015 |
| OTU_79_Genus_Alkaliphilus | 9.93E-01 | 9.93E-01 | -0.006 |

**Legend:** A positive mean log relative abundance means that the abundance is higher in IF patients, a negative mean log relative abundance means that the abundance is higher in the healthy controls

**Abbreviations:** IF, intestinal failure.

**Supplementary Table 2.** Differences in relative abundance between IF patients and healthy controls for all samples at family level

| Family | p-value | adjusted p-value | Differences in mean log relative abundance |
|---|---|---|---|
| Clostridiales vadinBB60 group | 4.10E-08 | 5.08E-06 | -3.155 |
| Coriobacteriaceae | 4.74E-07 | 2.94E-05 | -3.676 |
| Ruminococcaceae | 6.34E-06 | 2.62E-04 | -3.754 |
| Acidaminococcaceae | 8.82E-06 | 2.73E-04 | -3.667 |
| Family XIII | 1.54E-05 | 3.82E-04 | -1.995 |
| Alcaligenaceae | 2.90E-05 | 5.99E-04 | -3.991 |
| Enterobacteriaceae | 6.03E-05 | 1.07E-03 | 4.116 |
| Staphylococcaceae | 7.09E-05 | 1.07E-03 | 3.771 |
| Christensenellaceae | 7.73E-05 | 1.07E-03 | -2.571 |
| Victivallaceae | 9.51E-05 | 1.18E-03 | -1.722 |
| Clostridium sp. CAG:306 | 1.41E-04 | 1.59E-03 | -1.439 |
| Defluviitaleaceae | 1.54E-04 | 1.59E-03 | -1.523 |
| Erysipelotrichaceae | 1.77E-04 | 1.59E-03 | -2.727 |
| Bacteroidales S24-7 group | 1.79E-04 | 1.59E-03 | -3.019 |
| Bifidobacteriaceae | 5.10E-04 | 4.22E-03 | -2.771 |
| Rikenellaceae | 1.44E-03 | 1.11E-02 | -2.816 |
| Porphyromonadaceae | 1.58E-03 | 1.15E-02 | -2.584 |
| Mitochondria | 1.71E-03 | 1.18E-02 | -0.889 |
| Lachnospiraceae | 1.91E-03 | 1.25E-02 | -2.346 |
| Bacteroidaceae | 2.12E-03 | 1.32E-02 | -2.913 |
| Rhodospirillaceae | 2.63E-03 | 1.55E-02 | -2.088 |
| Thermoanaerobacteraceae | 8.75E-03 | 4.93E-02 | -0.774 |
| Pasteurellaceae | 1.01E-02 | 5.29E-02 | -1.480 |
| Desulfovibrionaceae | 1.02E-02 | 5.29E-02 | -2.108 |
| env.OPS 17 | 1.79E-02 | 6.39E-02 | -0.646 |
| Synergistaceae | 2.18E-02 | 6.39E-02 | -0.682 |
| Anaeroplasmataceae | 2.50E-02 | 6.39E-02 | -0.721 |
| Prevotellaceae | 2.52E-02 | 6.39E-02 | -1.668 |
| Rhizobiaceae | 2.60E-02 | 6.39E-02 | -0.619 |
| Caldicoprobacteraceae | 2.90E-02 | 6.39E-02 | -0.581 |
| Dictyoglomaceae | 3.16E-02 | 6.39E-02 | -0.619 |

| | | | |
|---|---|---|---|
| Alcanivoracaceae | 3.55E-02 | 6.39E-02 | -0.575 |
| Alicyclobacillaceae | 3.55E-02 | 6.39E-02 | -0.575 |
| Bartonellaceae | 3.55E-02 | 6.39E-02 | -0.575 |
| Bdellovibrionaceae | 3.55E-02 | 6.39E-02 | -0.575 |
| Bogoriellaceae | 3.55E-02 | 6.39E-02 | -0.575 |
| Brevibacteriaceae | 3.55E-02 | 6.39E-02 | -0.575 |
| Brucellaceae | 3.55E-02 | 6.39E-02 | -0.575 |
| BVA18 | 3.55E-02 | 6.39E-02 | -0.575 |
| Cytophagaceae | 3.55E-02 | 6.39E-02 | -0.575 |
| Family III | 3.55E-02 | 6.39E-02 | -0.575 |
| Gemmatimonadaceae | 3.55E-02 | 6.39E-02 | -0.575 |
| Geodermatophilaceae | 3.55E-02 | 6.39E-02 | -0.575 |
| Halieaceae | 3.55E-02 | 6.39E-02 | -0.575 |
| Hydrogenophilaceae | 3.55E-02 | 6.39E-02 | -0.575 |
| Hyphomicrobiaceae | 3.55E-02 | 6.39E-02 | -0.575 |
| Hyphomonadaceae | 3.55E-02 | 6.39E-02 | -0.575 |
| Iamiaceae | 3.55E-02 | 6.39E-02 | -0.575 |
| KD3-10 | 3.55E-02 | 6.39E-02 | -0.575 |
| Methylobacteriaceae | 3.55E-02 | 6.39E-02 | -0.575 |
| Methylophilaceae | 3.55E-02 | 6.39E-02 | -0.575 |
| Microbacteriaceae | 3.55E-02 | 6.39E-02 | -0.575 |
| Mycoplasmataceae | 3.55E-02 | 6.39E-02 | -0.575 |
| Nannocystaceae | 3.55E-02 | 6.39E-02 | -0.575 |
| Nitrospiraceae | 3.55E-02 | 6.39E-02 | -0.575 |
| Oceanospirillaceae | 3.55E-02 | 6.39E-02 | -0.575 |
| Oligoflexaceae | 3.55E-02 | 6.39E-02 | -0.575 |
| Parachlamydiaceae | 3.55E-02 | 6.39E-02 | -0.575 |
| Phycisphaeraceae | 3.55E-02 | 6.39E-02 | -0.575 |
| Pseudonocardiaceae | 3.55E-02 | 6.39E-02 | -0.575 |
| Rhodobacteraceae | 3.55E-02 | 6.39E-02 | -0.575 |
| Rhodocyclaceae | 3.55E-02 | 6.39E-02 | -0.575 |
| Rhodospirillales Incertae Sedis | 3.55E-02 | 6.39E-02 | -0.575 |
| SM2D12 | 3.55E-02 | 6.39E-02 | -0.575 |

| | | | |
|---|---|---|---|
| Spirochaetaceae | 3.55E-02 | 6.39E-02 | -0.575 |
| Spongiibacteraceae | 3.55E-02 | 6.39E-02 | -0.575 |
| Succinivibrionaceae | 3.55E-02 | 6.39E-02 | -0.575 |
| TM146 | 3.55E-02 | 6.39E-02 | -0.575 |
| Vulgatibacteraceae | 3.55E-02 | 6.39E-02 | -0.575 |
| Planctomycetaceae | 3.73E-02 | 6.61E-02 | -0.540 |
| Peptococcaceae | 4.39E-02 | 7.66E-02 | -0.802 |
| FamilyI | 4.47E-02 | 7.69E-02 | -0.540 |
| Cardiobacteriaceae | 4.66E-02 | 7.81E-02 | -0.525 |
| CFT112H7 | 4.66E-02 | 7.81E-02 | -0.525 |
| 1174-901-12 | 4.75E-02 | 7.85E-02 | -0.525 |
| Dermacoccaceae | 5.26E-02 | 8.58E-02 | -0.525 |
| Nitrospinaceae | 5.85E-02 | 9.42E-02 | -0.524 |
| Methanobacteriaceae | 6.36E-02 | 1.01E-01 | -0.545 |
| Holophagaceae | 7.05E-02 | 1.07E-01 | -0.496 |
| Nitrosomonadaceae | 7.05E-02 | 1.07E-01 | -0.496 |
| Family XI | 7.15E-02 | 1.07E-01 | 1.086 |
| NS9 marine group | 7.15E-02 | 1.07E-01 | -0.476 |
| Unknown Family | 7.15E-02 | 1.07E-01 | -0.476 |
| Leptospiraceae | 7.34E-02 | 1.08E-01 | -0.496 |
| Micromonosporaceae | 7.38E-02 | 1.08E-01 | -0.476 |
| Methylocystaceae | 7.47E-02 | 1.08E-01 | -0.496 |
| Sphingobacteriaceae | 8.07E-02 | 1.15E-01 | -0.496 |
| Deferribacteraceae | 8.68E-02 | 1.22E-01 | -0.460 |
| Syntrophorhabdaceae | 8.76E-02 | 1.22E-01 | -0.476 |
| Neisseriaceae | 8.92E-02 | 1.23E-01 | 1.003 |
| Verrucomicrobiaceae | 1.21E-01 | 1.65E-01 | -1.392 |
| Peptostreptococcaceae | 1.38E-01 | 1.86E-01 | -1.058 |
| Pseudomonadaceae | 1.47E-01 | 1.96E-01 | 1.206 |
| Caulobacteraceae | 1.61E-01 | 2.10E-01 | -0.368 |
| Sporichthyaceae | 1.61E-01 | 2.10E-01 | -0.377 |
| Lactobacillaceae | 1.87E-01 | 2.41E-01 | 1.376 |
| Aerococcaceae | 1.90E-01 | 2.43E-01 | -0.376 |

| | | | |
|---|---|---|---|
| Fusobacteriaceae | 1.98E-01 | 2.49E-01 | 0.598 |
| Chitinophagaceae | 1.99E-01 | 2.49E-01 | -0.357 |
| Campylobacteraceae | 2.00E-01 | 2.49E-01 | -0.442 |
| Acetobacteraceae | 2.28E-01 | 2.80E-01 | -0.361 |
| Bradyrhizobiaceae | 2.45E-01 | 2.98E-01 | -0.303 |
| Bacillaceae | 2.63E-01 | 3.17E-01 | -0.320 |
| Nocardiaceae | 2.70E-01 | 3.23E-01 | -0.389 |
| Oxalobacteraceae | 3.28E-01 | 3.87E-01 | -0.323 |
| Streptococcaceae | 3.57E-01 | 4.17E-01 | -0.563 |
| Eubacteriaceae | 3.81E-01 | 4.42E-01 | -0.487 |
| Leptotrichiaceae | 4.78E-01 | 5.48E-01 | 0.315 |
| Comamonadaceae | 4.99E-01 | 5.67E-01 | -0.191 |
| Sphingomonadaceae | 5.03E-01 | 5.67E-01 | -0.180 |
| Xanthomonadaceae | 5.18E-01 | 5.79E-01 | 0.376 |
| Micrococcaceae | 5.47E-01 | 6.06E-01 | -0.232 |
| Clostridiaceae 1 | 5.87E-01 | 6.44E-01 | -0.434 |
| Burkholderiaceae | 5.99E-01 | 6.52E-01 | -0.225 |
| Moraxellaceae | 6.10E-01 | 6.55E-01 | 0.207 |
| Carnobacteriaceae | 6.12E-01 | 6.55E-01 | -0.187 |
| Corynebacteriaceae | 6.43E-01 | 6.82E-01 | -0.208 |
| Aeromonadaceae | 6.59E-01 | 6.93E-01 | 0.353 |
| Veillonellaceae | 7.04E-01 | 7.34E-01 | -0.266 |
| Actinomycetaceae | 7.56E-01 | 7.82E-01 | 0.160 |
| Leuconostocaceae | 8.77E-01 | 8.99E-01 | 0.103 |
| Flavobacteriaceae | 8.91E-01 | 9.06E-01 | 0.052 |
| Clostridiaceae 2 | 9.87E-01 | 9.87E-01 | 0.010 |
| Paenibacillaceae | 9.87E-01 | 9.87E-01 | 0.007 |

**Legend:** A positive mean log relative abundance means that the abundance is higher in IF patients, a negative mean log relative abundance means that the abundance is higher in the healthy controls.

**Abbreviations:** IF, intestinal failure.

**Supplementary Table 3.** Concentration of SCFA and lactate for IF patients and healthy controls at first sample collection per g wet feces

| **Per g wet feces** | **n** | **Patients with IF** | **n** | **Healthy controls** | **p-value** |
|---|---|---|---|---|---|
| | | **n = 15** | | **n = 25** | |
| **SCFA** | 14[1] | | 21[1] | | |
| Acetic acid (C2), µmol/g | | 32.1 (20.1 – 66.2) | | 172 (130 – 222) | p < 0.001 |
| Acetic acid (C2), % | | 14.8 (8.94 – 21.8) | | 37.9 (34.2 - 43.6) | p < 0.001 |
| Propionic acid (C3), µmol/g | | 1.23 (0.19 – 6.50) | | 31.4 (28.7 – 51.4) | p < 0.001 |
| Propionic acid (C3), % | | 0.61 (0.11 – 1.85) | | 7.80 (5.70 – 10.7) | p < 0.001 |
| Butyric acid (C4), µmol/g | | 0.31 (0.18 – 2.06) | | 29.9 (22.6 – 36.9) | p < 0.001 |
| Butyric acid (C4), % | | 0.17 (0.06 – 1.23) | | 6.63 (3.39 – 7.98) | p < 0.001 |
| Valeric acid (C5), µmol/g | | 0.05 (0.01 – 0.97) | | 2.35 (1.11 – 4.82) | p < 0.001 |
| Valeric acid (C5), % | | 0.03 (0.00 – 0.13) | | 0.67 (0.19 – 1.08) | p < 0.001 |
| Caproic acid (C6), µmol/g | | 0.10 (0.02 – 0.17) | | 0.36 (0.24 – 2.86) | p < 0.001 |
| Caproic acid (C6), % | | 0.02 (0.01 – 0.12) | | 0.08 (0.04 – 0.75) | p = 0.037 |
| Heptanoic acid (C7), µmol/g | | 0.12 (0.02 – 0.23) | | 0.04 (0.02 – 0.11) | p = 0.273 |
| Heptanoic acid (C7), % | | 0.03 (0.01 – 0.14) | | 0.01 (0.00 – 0.03) | p = 0.040 |
| Octanoic acid (C8), µmol/g | | 0.02 (0.00 – 0.11) | | 0.09 (0.02 – 0.23) | p = 0.089 |
| Octanoic acid (C8), % | | 0.01 (0.00 – 0.11) | | 0.01 (0.00 – 0.06) | p = 0.713 |
| Total, µmol/g | | 34.6 (25.1 – 75.7) | | 255 (199 – 367) | p < 0.001 |
| Iso-butyric acid (iC4), µmol/g | | 0.09 (0.202 – 0.84) | | 4.27 (2.27 – 5.85) | p < 0.001 |
| Iso-butyric acid (iC4), % | | 0.08 (0.01 – 0.18) | | 1.06 (0.51 – 1.24) | p < 0.001 |
| Iso-valeric acid (iC5), µmol/g | | 0.15 (0.03 – 0.78) | | 3.88 (2.10 – 5.92) | p < 0.001 |
| Iso-valeric acid (iC5), % | | 0.08 (0.01 – 0.15) | | 0.96 (0.45 – 1.28) | p < 0.001 |
| Iso-caproic acid (iC6), µmol/g | | 0.07 (0.05 – 0.23) | | 0.22 (0.13 – 0.28) | p = 0.027 |
| Iso-caproic acid (iC6), % | | 0.04 (0.01 – 0.09) | | 0.04 (0.03 – 0.06) | p = 0.553 |
| **D-lactate, µg/g** | 8 | 185 (81 - 1358) | 20[2] | 53 (38 – 84) | p = 0.002 |
| **L-lactate, µg/g** | 8 | 168 (90 - 682) | 20[2] | 93 (41 - 150) | p = 0.055 |
| **Total lactate, µg/g** | 8 | 351 (157 – 2134) | 20[2] | 159 (92 – 207) | p = 0.007 |
| **% D-lactate** | 8 | 48 (42 – 57) | 20[2] | 34 (21 – 56) | p = 0.070 |

**Legend:** Values shown ad median (IQR) or n (%) unless stated otherwise. **[1]** For one patient and for 4 healthy controls, water content was not known at the first sample, [2] the same applies for 5 healthy controls regarding the water content of their lactate samples.

**Abbreviations:** IF, intestinal failure; IQR, SCFA, short-chain fatty acids.

**Supplementary Table 4.** Fecal water content, concentration of SCFA, lactate, and number of 16S rRNA gene copies for IF patients and healthy controls at last sample

| | n | Patients with IF | n | Healthy controls | p-value |
|---|---|---|---|---|---|
| | | **n = 15** | | **n = 25** | |
| **Fecal water content (%)** | 13 | 83.3 (75.0 – 85.7) | 25 | 65 (62-74) | p = 0.062 |
| **SCFA (per g dry feces)** | 14 | | 25 | | |
| Acetic acid (C2), µmol/g | | 265 (156 – 457) | | 323 (266-370) | p = 0.176 |
| Acetic acid (C2), % | | 90.1 (73.8 – 93.6) | | 67.6 (64.7-61.3) | p = 0.016 |
| Propionic acid (C3), µmol/g | | 15.0 (4.45 – 38.7) | | 64.0 (47.5-85.1) | p < 0.001 |
| Propionic acid (C3), % | | 4.79 (2.30 – 10.7) | | 13.7 (10.6-18.8) | p = 0.005 |
| Butyric acid (C4), µmol/g | | 13.5 (0.39 – 52.4) | | 54.3 (36.6-71.0) | p = 0.001 |
| Butyric acid (C4), % | | 3.91 (0.17 – 10.4) | | 11.6 (5.60-14.9) | p = 0.010 |
| Valeric acid (C5), µmol/g | | 1.64 (0.17 – 5.01) | | 4.94 (2.04-9.56) | p = 0.010 |
| Valeric acid (C5), % | | 0.38 (0.08 – 1.39) | | 1.39 (0.36-2.26) | p = 0.118 |
| Caproic acid (C6), µmol/g | | 0.46 (0.35 – 0.55) | | 0.51 (0.26-3.72) | p = 0.071 |
| Caproic acid (C6), % | | 0.14 (0.08 – 0.34) | | 0.12 (0.08-0.78) | p = 0.965 |
| Heptanoic acid (C7), µmol/g | | 0.21 (0.05 – 0.71) | | 0.07 (0.04-0.17) | p = 0.141 |
| Heptanoic acid (C7), % | | 0.08 (0.01 – 0.20) | | 0.01 (0.00-0.05) | p = 0.076 |
| Octanoic acid (C8), µmol/g | | 0.47 (0.06 – 1.60) | | 0.17 (0.04-0.34) | p = 0.166 |
| Octanoic acid (C8), % | | 0.26 (0.01 – 0.56) | | 0.01 (0.01-0.08) | p = 0.020 |
| Total, µmol/g | | 288 (188 – 529) | | 472 (397-592) | p = 0.019 |
| Iso-butyric acid (iC4), µmol/g | | 0.19 (0.41 – 5.66) | | 6.40 (3.73-10.2) | p = 0.006 |
| Iso-butyric acid (iC4), % | | 0.49 (0.13 – 1.97) | | 1.42 (0.92-2.17) | p = 0.125 |
| Iso-valeric acid (iC5), µmol/g | | 1.47 (0.41 – 6.42) | | 6.27 (3.48-10.2) | p = 0.013 |
| Iso-valeric acid (iC5), % | | 0.46 (0.12 – 2.26) | | 1.18 (0.82-14.9) | p = 0.216 |
| Iso-caproic acid (iC6), µmol/g | | 0.27 (0.13 – 1.03) | | 0.35 (0.26-0.48) | p = 0.784 |
| Iso-caproic acid (iC6), % | | 0.08 (0.04 – 0.25) | | 0.07 (0.05-0.09) | p = 0.460 |
| **D-lactate, µg/g dry feces** | 9 | 298 (172 – 1577) | 24 | 79 (58-156) | p < 0.001 |
| **L-lactate, µg/g dry feces** | 9 | 287 (210 – 1985) | 24 | 211 (102-257) | p = 0.018 |
| **Total lactate, µg/g dry feces** | 9 | 518 (383 – 3750) | 24 | 256 (193-376) | p < 0.001 |
| **% D-lactate per g dry feces** | 9 | 42 (34 – 57) | 24 | 33 (19-50) | p = 0.121 |
| **Log of 16S rRNA gene copy number per g dry feces** (IQR, range) | 14 | 10.8 (10.1 – 11.0, 9.41 – 11.10) | 25 | 11.1 (10.9-11.3, 10.7-11.7) | p = 0.001 |

| | | | | | |
|---|---|---|---|---|---|
| **Log of 16S rRNA gene copy number per g wet feces** (IQR, range) | 13 | 9.90 (9.48 – 10.5, 8.42 – 11.06) | 25 | 3.82 (3.33-4.37, 2.6-18.4) | p = 0.001 |

**Legend:** Values shown ad median (IQR) or n (%) unless stated otherwise.

**Abbreviations:** IF, intestinal failure; IQR, interquartile range; SCFA, short-chain fatty acids.

**Supplementary Table 5.** Differences in relative abundance between surgical IF and functional IF patients for all samples at OTU level

| OTU | p-value | adjusted p-value | Differences in mean log relative abundance |
|---|---|---|---|
| OTU_210_Genus_Lactobacillus | 3.06E-05 | 3.06E-03 | -1.506 |
| OTU_22_Genus_Lachnoclostridium | 3.76E-05 | 3.06E-03 | 3.763 |
| OTU_18_Genus_Lactobacillus | 4.59E-05 | 3.06E-03 | -2.409 |
| OTU_126_Genus_Eggerthella | 7.76E-05 | 3.40E-03 | 2.684 |
| OTU_255_Genus_Ruminiclostridium 5 | 8.51E-05 | 3.40E-03 | 1.816 |
| OTU_84_Genus_Lachnoclostridium | 1.06E-04 | 3.53E-03 | 3.108 |
| OTU_164_Genus_Capnocytophaga | 2.35E-04 | 6.51E-03 | -1.000 |
| OTU_6_Genus_Haemophilus | 2.60E-04 | 6.51E-03 | 2.272 |
| OTU_121_Genus_Dorea | 3.68E-04 | 7.62E-03 | 2.161 |
| OTU_51_Genus_Flavonifractor | 3.81E-04 | 7.62E-03 | 2.924 |
| OTU_56_Genus_Alistipes | 5.05E-04 | 8.65E-03 | 2.486 |
| OTU_45_Genus_Lachnoclostridium | 5.19E-04 | 8.65E-03 | 2.944 |
| OTU_166_Genus_Lactobacillus | 6.29E-04 | 9.68E-03 | -1.146 |
| OTU_96_Genus_Streptococcus | 7.12E-04 | 9.77E-03 | 1.694 |
| OTU_46_Genus_Prevotella 7 | 7.50E-04 | 9.77E-03 | -1.503 |
| OTU_21_Genus_Anaeroglobus | 7.81E-04 | 9.77E-03 | -1.849 |
| OTU_30_Genus_Hungatella | 8.75E-04 | 1.03E-02 | 2.913 |
| OTU_54_Genus_Proteus | 9.72E-04 | 1.08E-02 | -1.120 |
| OTU_94_Genus_Clostridium sensu stricto 1 | 1.07E-03 | 1.12E-02 | 2.042 |
| OTU_69_Family_Ruminococcaceae | 1.27E-03 | 1.27E-02 | 2.074 |
| OTU_34_Genus_Veillonella | 1.37E-03 | 1.30E-02 | -1.651 |
| OTU_36_Genus_Megasphaera | 1.50E-03 | 1.36E-02 | -2.076 |
| OTU_103_Genus_Clostridium sensu stricto 1 | 1.72E-03 | 1.50E-02 | 2.346 |
| OTU_12_Genus_Bacteroides | 2.10E-03 | 1.75E-02 | 2.752 |
| OTU_38_Genus_Lactobacillus | 2.41E-03 | 1.85E-02 | -1.511 |
| OTU_115_Genus_Selenomonas 3 | 2.41E-03 | 1.85E-02 | -1.206 |
| OTU_17_Genus_Lactobacillus | 2.71E-03 | 1.95E-02 | -1.708 |
| OTU_80_Family_Lachnospiraceae | 2.73E-03 | 1.95E-02 | 2.312 |
| OTU_3_Genus_Cronobacter | 3.02E-03 | 1.99E-02 | -2.372 |
| OTU_277_Genus_Xanthomonas | 3.06E-03 | 1.99E-02 | -0.916 |

| | | | |
|---|---|---|---|
| OTU_268_Genus_Holdemanella | 3.09E-03 | 1.99E-02 | -0.869 |
| OTU_128_Genus_Peptoclostridium | 3.21E-03 | 2.00E-02 | -1.232 |
| OTU_207_Genus_Allisonella | 3.68E-03 | 2.23E-02 | -0.871 |
| OTU_88_Family_CFT112H7 | 3.81E-03 | 2.24E-02 | -1.263 |
| OTU_226_Genus_Slackia | 3.98E-03 | 2.27E-02 | -0.849 |
| OTU_212_Genus_Hafnia | 4.98E-03 | 2.77E-02 | -0.723 |
| OTU_161_Genus_Neisseria | 5.84E-03 | 3.03E-02 | -0.654 |
| OTU_90_Genus_Thalassospira | 5.94E-03 | 3.03E-02 | -0.738 |
| OTU_58_Genus_Parabacteroides | 5.95E-03 | 3.03E-02 | 2.257 |
| OTU_147_Genus_Bergeyella | 6.06E-03 | 3.03E-02 | -0.649 |
| OTU_175_Genus_Comamonas | 6.79E-03 | 3.14E-02 | -0.635 |
| OTU_133_Genus_Bacteroides | 6.86E-03 | 3.14E-02 | 2.064 |
| OTU_71_Genus_Blautia | 6.88E-03 | 3.14E-02 | 1.909 |
| OTU_26_Genus_Blautia | 7.09E-03 | 3.14E-02 | 1.779 |
| OTU_92_Genus_Bilophila | 7.18E-03 | 3.14E-02 | 1.856 |
| OTU_19_Genus_Tyzzerella 4 | 7.22E-03 | 3.14E-02 | 2.235 |
| OTU_93_Genus_Blautia | 7.72E-03 | 3.29E-02 | 1.446 |
| OTU_138_Genus_Gemella | 8.39E-03 | 3.50E-02 | 1.227 |
| OTU_16_Genus_Lachnoclostridium | 8.56E-03 | 3.50E-02 | 2.370 |
| OTU_97_Genus_Dorea | 9.25E-03 | 3.70E-02 | 1.282 |
| OTU_4_Genus_Lactobacillus | 9.50E-03 | 3.72E-02 | -1.470 |
| OTU_98_Genus_Lachnospiraceae NK4A136 group | 1.16E-02 | 4.37E-02 | 1.391 |
| OTU_695_Genus_Bacteroides | 1.16E-02 | 4.37E-02 | 1.552 |
| OTU_113_Genus_Lachnoclostridium | 1.45E-02 | 5.22E-02 | 1.651 |
| OTU_171_Genus_Staphylococcus | 1.46E-02 | 5.22E-02 | 1.022 |
| OTU_204_Genus_Ruminiclostridium | 1.49E-02 | 5.22E-02 | 1.298 |
| OTU_215_Genus_Erysipelatoclostridium | 1.50E-02 | 5.22E-02 | 1.362 |
| OTU_182_Genus_Incertae Sedis | 1.51E-02 | 5.22E-02 | 1.195 |
| OTU_199_Genus_Anaerostipes | 1.56E-02 | 5.29E-02 | 1.319 |
| OTU_165_Genus_Campylobacter | 1.60E-02 | 5.34E-02 | -0.985 |
| OTU_101_Genus_Thalassospira | 1.64E-02 | 5.39E-02 | -0.674 |
| OTU_67_Genus_Collinsella | 1.67E-02 | 5.39E-02 | 1.280 |
| OTU_130_Genus_Acidaminococcus | 1.73E-02 | 5.48E-02 | -0.682 |

| | | | |
|---|---|---|---|
| OTU_86_Genus_Parasutterella | 1.75E-02 | 5.48E-02 | -0.993 |
| OTU_152_Genus_Christensenellaceae R-7 group | 1.78E-02 | 5.49E-02 | 1.268 |
| OTU_191_Genus_Lachnospiraceae UCG-008 | 1.85E-02 | 5.61E-02 | 0.924 |
| OTU_85_Genus_Lachnospiraceae NK4A136 group | 2.00E-02 | 5.96E-02 | 1.505 |
| OTU_49_Genus_Roseburia | 2.03E-02 | 5.97E-02 | 1.533 |
| OTU_52_Genus_Peptoclostridium | 2.13E-02 | 6.18E-02 | 1.643 |
| OTU_75_Family_Ruminococcaceae | 2.52E-02 | 7.19E-02 | 1.249 |
| OTU_74_Genus_Ruminococcaceae UCG-002 | 2.58E-02 | 7.28E-02 | 1.327 |
| OTU_23_Genus_Alistipes | 3.00E-02 | 8.34E-02 | 1.717 |
| OTU_62_Genus_Parabacteroides | 3.08E-02 | 8.43E-02 | 1.487 |
| OTU_125_Genus_Ruminococcaceae UCG-003 | 3.50E-02 | 9.45E-02 | 1.194 |
| OTU_20_Genus_Aeromonas | 3.75E-02 | 1.00E-01 | -0.672 |
| OTU_5_Genus_Akkermansia | 4.09E-02 | 1.07E-01 | 1.411 |
| OTU_14_Genus_Staphylococcus | 4.10E-02 | 1.07E-01 | 1.637 |
| OTU_82_Genus_Bacteroides | 4.20E-02 | 1.08E-01 | 1.119 |
| OTU_33_Genus_Pseudobutyrivibrio | 4.54E-02 | 1.14E-01 | 1.016 |
| OTU_157_Genus_Veillonella | 4.55E-02 | 1.14E-01 | 1.038 |
| OTU_55_Genus_Blautia | 4.84E-02 | 1.20E-01 | 0.951 |
| OTU_40_Genus_Phascolarctobacterium | 5.74E-02 | 1.40E-01 | -0.621 |
| OTU_15_Genus_Tyzzerella | 5.88E-02 | 1.42E-01 | 1.188 |
| OTU_31_Genus_Faecalibacterium | 6.75E-02 | 1.61E-01 | 0.876 |
| OTU_145_Genus_Bacteroides | 6.97E-02 | 1.64E-01 | -0.641 |
| OTU_230_Genus_Coprobacter | 7.37E-02 | 1.71E-01 | 0.815 |
| OTU_201_Family_Lachnospiraceae | 7.43E-02 | 1.71E-01 | 0.872 |
| OTU_205_Genus_Alistipes | 7.71E-02 | 1.75E-01 | 0.837 |
| OTU_83_Genus_Ruminococcus 1 | 7.81E-02 | 1.75E-01 | 0.900 |
| OTU_220_Family_Lachnospiraceae | 7.89E-02 | 1.75E-01 | 1.001 |
| OTU_2_Genus_Pseudomonas | 8.03E-02 | 1.76E-01 | -1.258 |
| OTU_114_Genus_Faecalibacterium | 9.13E-02 | 1.99E-01 | 0.733 |
| OTU_106_Genus_[Eubacterium] hallii group | 9.33E-02 | 2.01E-01 | 0.754 |
| OTU_65_Genus_Ruminococcaceae UCG-002 | 1.01E-01 | 2.15E-01 | 0.827 |
| OTU_29_Family_Lachnospiraceae | 1.07E-01 | 2.26E-01 | 1.251 |
| OTU_240_Genus_Tyzzerella | 1.12E-01 | 2.34E-01 | 0.723 |

| | | | |
|---|---|---|---|
| OTU_13_Genus_Bacteroides | 1.21E-01 | 2.50E-01 | 1.046 |
| OTU_64_Genus_Ruminococcus 1 | 1.23E-01 | 2.51E-01 | 0.692 |
| OTU_193_Genus_Erysipelatoclostridium | 1.27E-01 | 2.57E-01 | 0.801 |
| OTU_116_Genus_Ruminococcaceae UCG-005 | 1.36E-01 | 2.71E-01 | 0.757 |
| OTU_68_Genus_Ruminococcaceae UCG-005 | 1.37E-01 | 2.71E-01 | 0.897 |
| OTU_235_Genus_Lachnospiraceae NK4A136 group | 1.45E-01 | 2.84E-01 | 0.668 |
| OTU_261_Genus_Desulfovibrio | 1.48E-01 | 2.86E-01 | -0.474 |
| OTU_42_Genus_Ruminococcus 2 | 1.49E-01 | 2.86E-01 | 0.799 |
| OTU_148_Family_Ruminococcaceae | 1.58E-01 | 3.00E-01 | 0.596 |
| OTU_194_Genus_Butyricimonas | 1.60E-01 | 3.01E-01 | 0.618 |
| OTU_135_Genus_Lachnoclostridium | 1.65E-01 | 3.06E-01 | 0.717 |
| OTU_112_Genus_Ruminiclostridium 5 | 1.65E-01 | 3.06E-01 | 0.690 |
| OTU_39_Genus_Alistipes | 1.68E-01 | 3.06E-01 | 0.928 |
| OTU_108_Genus_Fusicatenibacter | 1.68E-01 | 3.06E-01 | 0.687 |
| OTU_141_Genus_Neisseria | 1.72E-01 | 3.09E-01 | -0.480 |
| OTU_76_Genus_Anaeroglobus | 1.84E-01 | 3.29E-01 | -0.483 |
| OTU_225_Genus_Actinomyces | 1.97E-01 | 3.49E-01 | 0.527 |
| OTU_946_Genus_Veillonella | 2.01E-01 | 3.52E-01 | -0.436 |
| OTU_37_Genus_Lachnoclostridium | 2.03E-01 | 3.52E-01 | 0.645 |
| OTU_144_Genus_Ruminococcaceae UCG-004 | 2.05E-01 | 3.54E-01 | 0.654 |
| OTU_8_Genus_Morganella | 2.11E-01 | 3.59E-01 | 1.105 |
| OTU_28_Genus_Bacteroides | 2.12E-01 | 3.59E-01 | 0.925 |
| OTU_99_Genus_Bacteroides | 2.35E-01 | 3.93E-01 | -0.475 |
| OTU_119_Genus_Ruminococcus 1 | 2.36E-01 | 3.93E-01 | 0.468 |
| OTU_89_Genus_Lachnospira | 2.39E-01 | 3.95E-01 | 0.764 |
| OTU_63_Genus_Lachnoclostridium | 2.41E-01 | 3.95E-01 | 0.740 |
| OTU_208_Genus_Ruminiclostridium | 2.78E-01 | 4.48E-01 | 0.473 |
| OTU_143_Genus_Thalassospira | 2.81E-01 | 4.48E-01 | 0.499 |
| OTU_24_Genus_Bifidobacterium | 2.82E-01 | 4.48E-01 | -0.565 |
| OTU_276_Genus_Finegoldia | 2.84E-01 | 4.48E-01 | -0.398 |
| OTU_91_Genus_Sutterella | 2.85E-01 | 4.48E-01 | 0.755 |
| OTU_110_Genus_Alistipes | 3.08E-01 | 4.81E-01 | 0.464 |
| OTU_195_Genus_Campylobacter | 3.10E-01 | 4.81E-01 | -0.307 |

| | | | |
|---|---|---|---|
| OTU_104_Genus_Ruminiclostridium 5 | 3.15E-01 | 4.85E-01 | 0.452 |
| OTU_73_Genus_Coprococcus 3 | 3.31E-01 | 5.06E-01 | 0.431 |
| OTU_48_Genus_Blautia | 3.36E-01 | 5.08E-01 | 0.352 |
| OTU_763_Genus_Veillonella | 3.38E-01 | 5.08E-01 | -0.427 |
| OTU_305_Genus_Lachnoanaerobaculum | 3.44E-01 | 5.13E-01 | -0.323 |
| OTU_102_Genus_Odoribacter | 3.46E-01 | 5.13E-01 | 0.552 |
| OTU_7_Genus_Prevotella 9 | 3.50E-01 | 5.14E-01 | 0.453 |
| OTU_59_Genus_Barnesiella | 3.57E-01 | 5.21E-01 | 0.501 |
| OTU_943_Class_Bacteroidetes VC2.1 Bac22 | 3.69E-01 | 5.35E-01 | -0.323 |
| OTU_302_Genus_Peptostreptococcus | 3.75E-01 | 5.39E-01 | 0.336 |
| OTU_181_Genus_Dielma | 3.80E-01 | 5.43E-01 | 0.398 |
| OTU_41_Genus_Subdoligranulum | 3.85E-01 | 5.46E-01 | 0.488 |
| OTU_248_Genus_Johnsonella | 3.89E-01 | 5.47E-01 | 0.315 |
| OTU_25_Genus_Streptococcus | 3.96E-01 | 5.54E-01 | 0.606 |
| OTU_44_Genus_[Eubacterium] coprostanoligenes group | 3.99E-01 | 5.54E-01 | 0.348 |
| OTU_244_Genus_Blautia | 4.01E-01 | 5.54E-01 | 0.427 |
| OTU_57_Genus_Subdoligranulum | 4.08E-01 | 5.59E-01 | 0.374 |
| OTU_231_Genus_Leuconostoc | 4.21E-01 | 5.68E-01 | 0.452 |
| OTU_11_Genus_Rikenella | 4.22E-01 | 5.68E-01 | 0.555 |
| OTU_142_Genus_[Eubacterium] coprostanoligenes group | 4.23E-01 | 5.68E-01 | 0.369 |
| OTU_140_Genus_Ruminococcaceae NK4A214 group | 4.48E-01 | 5.94E-01 | 0.371 |
| OTU_154_Genus_Incertae Sedis | 4.48E-01 | 5.94E-01 | 0.309 |
| OTU_43_Family_Lachnospiraceae | 4.72E-01 | 6.20E-01 | 0.393 |
| OTU_150_Family_Veillonellaceae | 4.75E-01 | 6.20E-01 | 0.344 |
| OTU_27_Genus_Neisseria | 4.79E-01 | 6.22E-01 | -0.326 |
| OTU_9_Genus_Veillonella | 4.95E-01 | 6.39E-01 | -0.499 |
| OTU_159_Class_Bacteroidetes VC2.1 Bac22 | 5.24E-01 | 6.72E-01 | 0.306 |
| OTU_160_Genus_Paraprevotella | 5.34E-01 | 6.80E-01 | 0.254 |
| OTU_87_Genus_[Eubacterium] coprostanoligenes group | 5.51E-01 | 6.97E-01 | -0.256 |
| OTU_72_Genus_Phascolarctobacterium | 5.60E-01 | 7.05E-01 | 0.274 |
| OTU_137_Genus_Lachnospiraceae UCG-008 | 5.67E-01 | 7.05E-01 | 0.219 |
| OTU_100_Genus_Coprococcus 2 | 5.67E-01 | 7.05E-01 | 0.255 |
| OTU_131_Genus_Blautia | 5.73E-01 | 7.08E-01 | 0.221 |

| | | | |
|---|---|---|---|
| OTU_247_Family_Coriobacteriaceae | 5.91E-01 | 7.25E-01 | 0.219 |
| OTU_78_Genus_Blautia | 5.94E-01 | 7.25E-01 | 0.261 |
| OTU_149_Genus_Lactococcus | 6.21E-01 | 7.53E-01 | -0.217 |
| OTU_60_Genus_Anaerostipes | 6.37E-01 | 7.64E-01 | 0.235 |
| OTU_196_Genus_Ruminococcaceae NK4A214 group | 6.38E-01 | 7.64E-01 | -0.160 |
| OTU_206_Genus_Varibaculum | 6.45E-01 | 7.66E-01 | -0.151 |
| OTU_32_Genus_Bacteroides | 6.48E-01 | 7.66E-01 | 0.297 |
| OTU_10_Family_Bacteroidales S24-7 group | 6.90E-01 | 8.09E-01 | 0.235 |
| OTU_155_Genus_Enterorhabdus | 6.92E-01 | 8.09E-01 | 0.188 |
| OTU_184_Genus_Anaerosporobacter | 7.20E-01 | 8.37E-01 | -0.119 |
| OTU_1_Genus_Escherichia-Shigella | 7.26E-01 | 8.40E-01 | 0.320 |
| OTU_35_Genus_Aggregatibacter | 7.33E-01 | 8.43E-01 | 0.166 |
| OTU_158_Genus_Peptoniphilus | 7.43E-01 | 8.47E-01 | -0.132 |
| OTU_398_Genus_Ruminococcaceae UCG-014 | 7.50E-01 | 8.47E-01 | -0.144 |
| OTU_47_Genus_Dialister | 7.51E-01 | 8.47E-01 | -0.197 |
| OTU_347_Genus_Lachnoclostridium | 7.54E-01 | 8.47E-01 | -0.124 |
| OTU_107_Genus_Lachnoclostridium | 7.67E-01 | 8.57E-01 | 0.126 |
| OTU_172_Family_Ruminococcaceae | 7.75E-01 | 8.59E-01 | -0.108 |
| OTU_61_Genus_Clostridium sensu stricto 1 | 7.77E-01 | 8.59E-01 | 0.218 |
| OTU_117_Genus_Epulopiscium | 8.06E-01 | 8.86E-01 | -0.094 |
| OTU_53_Genus_Fusobacterium | 8.20E-01 | 8.92E-01 | -0.162 |
| OTU_162_Genus_[Eubacterium] hallii group | 8.21E-01 | 8.92E-01 | -0.087 |
| OTU_136_Genus_Ruminiclostridium 5 | 8.26E-01 | 8.93E-01 | -0.085 |
| OTU_209_Genus_Victivallis | 8.41E-01 | 9.04E-01 | 0.086 |
| OTU_216_Genus_Alistipes | 8.46E-01 | 9.04E-01 | 0.078 |
| OTU_227_Genus_Lachnospira | 8.59E-01 | 9.14E-01 | 0.071 |
| OTU_79_Genus_Alkaliphilus | 8.69E-01 | 9.20E-01 | -0.068 |
| OTU_1064_Genus_Sutterella | 8.79E-01 | 9.23E-01 | 0.068 |
| OTU_606_Genus_Akkermansia | 8.82E-01 | 9.23E-01 | 0.068 |
| OTU_81_Genus_Paraprevotella | 8.86E-01 | 9.23E-01 | 0.068 |
| OTU_167_Genus_Ruminococcus 1 | 8.96E-01 | 9.28E-01 | 0.049 |
| OTU_156_Genus_Ruminiclostridium 9 | 9.04E-01 | 9.31E-01 | 0.055 |
| OTU_139_Genus_Stenotrophomonas | 9.10E-01 | 9.31E-01 | -0.042 |

| | | | |
|---|---|---|---|
| OTU_218_Family_Lachnospiraceae | 9.13E-01 | 9.31E-01 | 0.046 |
| OTU_50_Genus_Christensenellaceae R-7 group | 9.17E-01 | 9.31E-01 | 0.039 |
| OTU_224_Order_Gastranaerophilales | 9.25E-01 | 9.34E-01 | -0.032 |
| OTU_213_Order_Gastranaerophilales | 9.84E-01 | 9.87E-01 | 0.008 |
| OTU_95_Genus_[Eubacterium] oxidoreducens group | 9.87E-01 | 9.87E-01 | 0.008 |

**Legend:** A positive mean log relative abundance means that the abundance is higher in functional IF patients, a negative mean log relative abundance means that the abundance is higher in surgical IF patients.

**Abbreviations:** IF, intestinal failure.

**Supplementary Table 6.** Differences in relative abundance between functional and surgical IF patients for all samples at family level

| Family | p-value | adjusted p-value | Differences in mean log relative abundance |
|---|---|---|---|
| Erysipelotrichaceae | 7,85E-06 | 6,94E-04 | 2.462 |
| Lachnospiraceae | 1.12E-05 | 6.94E-04 | 2.511 |
| Ruminococcaceae | 3.89E-05 | 1.28E-03 | 2.200 |
| Coriobacteriaceae | 5.07E-05 | 1.28E-03 | 1.998 |
| Lactobacillaceae | 5.16E-05 | 1.28E-03 | -2.942 |
| Mitochondria | 6.41E-04 | 1.33E-02 | -0.636 |
| Aerococcaceae | 7.70E-04 | 1.36E-02 | -0.566 |
| Pasteurellaceae | 1.01E-03 | 1.57E-02 | 2.067 |
| Clostridiaceae 1 | 1.50E-03 | 1.93E-02 | 2.402 |
| Desulfovibrionaceae | 1.88E-03 | 1.93E-02 | 2.005 |
| NS9 marine group | 2.27E-03 | 1.93E-02 | -0.496 |
| Flavobacteriaceae | 2.40E-03 | 1.93E-02 | -0.887 |
| Family XIII | 2.83E-03 | 1.93E-02 | 1.124 |
| Methylocystaceae | 2.94E-03 | 1.93E-02 | -0.489 |
| Rikenellaceae | 3.07E-03 | 1.93E-02 | 2.417 |
| Sphingobacteriaceae | 3.08E-03 | 1.93E-02 | -0.489 |
| KD3-10 | 3.16E-03 | 1.93E-02 | -0.489 |
| Spirochaetaceae | 3.19E-03 | 1.93E-02 | -0.532 |
| Acetobacteraceae | 3.37E-03 | 1.93E-02 | -0.533 |
| Nitrospiraceae | 3.39E-03 | 1.93E-02 | -0.489 |
| Alcanivoracaceae | 3.43E-03 | 1.93E-02 | -0.489 |
| Bogoriellaceae | 3.43E-03 | 1.93E-02 | -0.489 |
| CFT112H7 | 3.71E-03 | 1.94E-02 | -1.267 |
| Methylobacteriaceae | 3.75E-03 | 1.94E-02 | -0.489 |
| Planctomycetaceae | 4.50E-03 | 2.07E-02 | -0.436 |
| Dictyoglomaceae | 4.68E-03 | 2.07E-02 | -0.462 |
| Iamiaceae | 4.68E-03 | 2.07E-02 | -0.462 |
| Rhizobiaceae | 4.68E-03 | 2.07E-02 | -0.462 |

| | | | |
|---|---|---|---|
| Paenibacillaceae | 5.18E-03 | 2.21E-02 | -0.561 |
| Porphyromonadaceae | 5.80E-03 | 2.33E-02 | 1.899 |
| Pseudonocardiaceae | 5.96E-03 | 2.33E-02 | -0.429 |
| Cardiobacteriaceae | 6.01E-03 | 2.33E-02 | -0.479 |
| Caldicoprobacteraceae | 6.64E-03 | 2.37E-02 | -0.429 |
| Microbacteriaceae | 6.65E-03 | 2.37E-02 | -0.426 |
| Streptococcaceae | 6.68E-03 | 2.37E-02 | 1.495 |
| Bacteroidaceae | 7.44E-03 | 2.39E-02 | 2.060 |
| BVA18 | 8.29E-03 | 2.39E-02 | -0.409 |
| Gemmatimonadaceae | 8.29E-03 | 2.39E-02 | -0.409 |
| Hyphomicrobiaceae | 8.29E-03 | 2.39E-02 | -0.409 |
| Oceanospirillaceae | 8.29E-03 | 2.39E-02 | -0.409 |
| Oligoflexaceae | 8.29E-03 | 2.39E-02 | -0.409 |
| SM2D12 | 8.29E-03 | 2.39E-02 | -0.409 |
| TM146 | 8.29E-03 | 2.39E-02 | -0.409 |
| env.OPS 17 | 8.98E-03 | 2.53E-02 | -0.453 |
| Synergistaceae | 9.98E-03 | 2.70E-02 | -0.499 |
| Caulobacteraceae | 1.00E-02 | 2.70E-02 | -0.369 |
| Halieaceae | 1.12E-02 | 2.78E-02 | -0.396 |
| Hyphomonadaceae | 1.12E-02 | 2.78E-02 | -0.396 |
| Nannocystaceae | 1.12E-02 | 2.78E-02 | -0.396 |
| Peptostreptococcaceae | 1.14E-02 | 2.78E-02 | 1.736 |
| Staphylococcaceae | 1.15E-02 | 2.78E-02 | 1.831 |
| Anaeroplasmataceae | 1.25E-02 | 2.93E-02 | -0.426 |
| Micromonosporaceae | 1.35E-02 | 2.93E-02 | -0.396 |
| Holophagaceae | 1.35E-02 | 2.93E-02 | -0.409 |
| Bradyrhizobiaceae | 1.42E-02 | 2.93E-02 | -0.409 |
| Unknown Family | 1.46E-02 | 2.93E-02 | -0.397 |
| Cytophagaceae | 1.47E-02 | 2.93E-02 | -0.385 |
| Phycisphaeraceae | 1.47E-02 | 2.93E-02 | -0.385 |
| Vulgatibacteraceae | 1.47E-02 | 2.93E-02 | -0.385 |
| Leptospiraceae | 1.48E-02 | 2.93E-02 | -0.409 |
| Nitrospinaceae | 1.48E-02 | 2.93E-02 | -0.409 |

| | | | |
|---|---|---|---|
| Brevibacteriaceae | 1.49E-02 | 2.93E-02 | -0.409 |
| Geodermatophilaceae | 1.49E-02 | 2.93E-02 | -0.409 |
| Actinomycetaceae | 1.59E-02 | 3.06E-02 | 1.008 |
| Parachlamydiaceae | 1.60E-02 | 3.06E-02 | -0.409 |
| Thermoanaerobacteraceae | 1.63E-02 | 3.06E-02 | -0.409 |
| Family III | 1.84E-02 | 3.32E-02 | -0.376 |
| Spongiibacteraceae | 1.84E-02 | 3.32E-02 | -0.376 |
| Family XI | 1.85E-02 | 3.32E-02 | 1.158 |
| Succinivibrionaceae | 1.94E-02 | 3.40E-02 | -0.416 |
| Syntrophorhabdaceae | 1.95E-02 | 3.40E-02 | -0.396 |
| Dermacoccaceae | 1.98E-02 | 3.41E-02 | -0.393 |
| Deferribacteraceae | 2.18E-02 | 3.70E-02 | -0.352 |
| Bartonellaceae | 2.34E-02 | 3.92E-02 | -0.396 |
| Rhodocyclaceae | 2.67E-02 | 4.41E-02 | -0.363 |
| 1174-901-12 | 2.95E-02 | 4.82E-02 | -0.363 |
| Rhodospirillales Incertae Sedis | 3.59E-02 | 5.78E-02 | -0.343 |
| Aeromonadaceae | 3.66E-02 | 5.81E-02 | -0.676 |
| Clostridium sp. CAG:306 | 3.79E-02 | 5.94E-02 | -0.432 |
| Verrucomicrobiaceae | 3.87E-02 | 5.99E-02 | 1.413 |
| FamilyI | 3.91E-02 | 5.99E-02 | -0.407 |
| Christensenellaceae | 4.16E-02 | 6.20E-02 | 0.937 |
| Bdellovibrionaceae | 4.20E-02 | 6.20E-02 | -0.333 |
| Hydrogenophilaceae | 4.20E-02 | 6.20E-02 | -0.333 |
| Nitrosomonadaceae | 4.80E-02 | 7.00E-02 | -0.343 |
| Campylobacteraceae | 5.39E-02 | 7.77E-02 | -0.868 |
| Methylophilaceae | 5.75E-02 | 8.19E-02 | -0.327 |
| Moraxellaceae | 5.96E-02 | 8.40E-02 | -0.494 |
| Pseudomonadaceae | 6.28E-02 | 8.74E-02 | -1.333 |
| Brucellaceae | 6.41E-02 | 8.82E-02 | -0.357 |
| Methanobacteriaceae | 6.61E-02 | 9.00E-02 | -0.300 |
| Mycoplasmataceae | 7.15E-02 | 9.64E-02 | -0.301 |
| Chitinophagaceae | 9.07E-02 | 1.21E-01 | -0.297 |
| Rhodobacteraceae | 1.00E-01 | 1.32E-01 | -0.333 |

| | | | |
|---|---|---|---|
| Nocardiaceae | 1.14E-01 | 1.48E-01 | -0.347 |
| Micrococcaceae | 1.90E-01 | 2.45E-01 | 0.420 |
| Oxalobacteraceae | 2.58E-01 | 3.29E-01 | -0.264 |
| Bacillaceae | 2.71E-01 | 3.43E-01 | -0.208 |
| Bifidobacteriaceae | 2.83E-01 | 3.54E-01 | -0.555 |
| Xanthomonadaceae | 2.94E-01 | 3.65E-01 | -0.451 |
| Alicyclobacillaceae | 3.04E-01 | 3.73E-01 | -0.181 |
| Burkholderiaceae | 3.17E-01 | 3.83E-01 | 0.313 |
| Sporichthyaceae | 3.18E-01 | 3.83E-01 | -0.178 |
| Peptococcaceae | 3.49E-01 | 4.16E-01 | 0.328 |
| Clostridiales vadinBB60 group | 3.65E-01 | 4.31E-01 | 0.394 |
| Neisseriaceae | 3.79E-01 | 4.43E-01 | -0.418 |
| Corynebacteriaceae | 3.94E-01 | 4.57E-01 | 0.301 |
| Veillonellaceae | 4.24E-01 | 4.84E-01 | -0.496 |
| Leuconostocaceae | 4.25E-01 | 4.84E-01 | 0.447 |
| Sphingomonadaceae | 4.62E-01 | 5.21E-01 | -0.127 |
| Defluviitaleaceae | 5.14E-01 | 5.74E-01 | -0.158 |
| Comamonadaceae | 5.25E-01 | 5.81E-01 | -0.199 |
| Bacteroidales S24-7 group | 5.88E-01 | 6.45E-01 | 0.315 |
| Carnobacteriaceae | 7.02E-01 | 7.63E-01 | -0.125 |
| Eubacteriaceae | 7.09E-01 | 7.64E-01 | -0.132 |
| Prevotellaceae | 7.24E-01 | 7.68E-01 | -0.173 |
| Victivallaceae | 7.28E-01 | 7.68E-01 | 0.149 |
| Fusobacteriaceae | 7.31E-01 | 7.68E-01 | -0.242 |
| Enterobacteriaceae | 7.80E-01 | 8.13E-01 | -0.196 |
| Clostridiaceae 2 | 8.62E-01 | 8.91E-01 | -0.072 |
| Leptotrichiaceae | 9.27E-01 | 9.50E-01 | 0.039 |
| Acidaminococcaceae | 9.56E-01 | 9.68E-01 | 0.027 |
| Rhodospirillaceae | 9.61E-01 | 9.68E-01 | 0.025 |
| Alcaligenaceae | 9.77E-01 | 9.77E-01 | 0.021 |

**Legend:** A positive mean log relative abundance means that the abundance is higher in functional IF patients, a negative mean log relative abundance means that the abundance is higher in the surgical IF patients.
**Abbreviations:** IF, intestinal failure.

**Supplementary Table 7.** Concentration of SCFA and lactate for surgical IF and functional IF patients at first sample collection per g dry feces

| **Per g dry feces** | **n** | **Surgical IF (n = 8)** | **n** | **Functional IF (n = 7)** | **p-value** |
|---|---|---|---|---|---|
| **SCFA** | 8 | | 7 | | |
| Acetic acid (C2), µmol/g | | 226 (153-340) | | 86.8 (78.1-616) | 0.613 |
| Acetic acid (C2), % | | 94.0 (90.2-96.8) | | 83.4 (76.0-91.8) | **0.014** |
| Propionic acid (C3), µmol/g | | 8.68 (0.84-17.6) | | 5.47 (3.24-155) | 0.536 |
| Propionic acid (C3), % | | 2.66 (0.47-4.49) | | 5.79 (3.64-15.0) | **0.040** |
| Butyric acid (C4), µmol/g | | 1.57 (1.16-2.69) | | 5.18 (0.92-78.5) | 0.281 |
| Butyric acid (C4), % | | 0.75 (0.58-0.91) | | 4.10 (1.85-6.12) | 0.072 |
| Valeric acid (C5), µmol/g | | 0.19 (0.09-0.29) | | 5.66 (0.17-8.01) | 0.189 |
| Valeric acid (C5), % | | 0.08 (0.04-0.13) | | 0.65 (0.18-0.92) | **0.002** |
| Caproic acid (C6), µmol/g | | 0.44 (0.33-0.48) | | 0.52 (0.33-0.80) | 0.536 |
| Caproic acid (C6), % | | 0.18 (0.08-0.25) | | 0.35 (0.06-0.61) | 0.536 |
| Heptanoic acid (C7), µmol/g | | 0.68 (0.28-0.90) | | 0.71 (0.44-0.87) | 0.955 |
| Heptanoic acid (C7), % | | 0.27 (0.08-0.47) | | 0.50 (0.14-0.76) | 0.281 |
| Octanoic acid (C8), µmol/g | | 0.37 (0.06-0.98) | | 0.00 (0.00-0.18) | 0.054 |
| Octanoic acid (C8), % | | 0.20 (0.02-0.42) | | 0.00 (0.00-0.20) | 0.094 |
| Total, µmol/g | | 245 (173-362) | | 103 (89.1-917) | 0.613 |
| Iso-butyric acid (iC4), µmol/g | | 0.40 (0.21-1.78) | | 2.32 (0.09-9.37) | 0.336 |
| Iso-butyric acid (iC4), % | | 0.19 (0.12-0.47) | | 1.00 (0.15-1.78) | 0.072 |
| Iso-valeric acid (iC5), µmol/g | | 0.86 (0.28-1.19) | | 2.26 (0.11-6.05) | 0.336 |
| Iso-valeric acid (iC5), % | | 0.34 (0.14-0.48) | | 0.73 (0.14-1.94) | 0.189 |
| Iso-caproic acid (iC6), µmol/g | | 0.40 (0.21-0.63) | | 0.89 (0.10-1.42) | 0.397 |
| Iso-caproic acid (iC6), % | | 0.17 (0.10-0.29) | | 0.21 (0.05-0.86) | 0.694 |
| **D-lactate, µg/g** | 5 | 2052 (1427-9042) | 3 | 298 (189-*) | 0.143 |
| **L-lactate, µg/g** | 5 | 2525 (1276-8152) | 3 | 287 (220-*) | 0.143 |
| **Total lactate, µg/g** | 5 | 4578 (2702-17193) | 3 | 518 (476-*) | 0.143 |
| **% D-lactate** | 5 | 0.51 (0.43-0.62) | 3 | 0.44 (0.40-*) | 0.571 |

**Legend:** Values shown ad median (IQR) or n (%) unless stated otherwise. * Due to the number of patients it was not possible to calculate an IQR.

**Abbreviations:** IF, intestinal failure; IQR, SCFA, short-chain fatty acids.

**Supplementary Table 8.** General linear mixed model for clinical variables and % of short-chain fatty acids

| % | c2 | | c3 | | ic4 | | c4 | | ic5 | | c5 | |
|---|---|---|---|---|---|---|---|---|---|---|---|---|
| | **β** | **p-value unadjusted/ adjusted** | **β** | **p-value unadjusted/ adjusted** | **β** | **p-value unadjusted/ adjusted** | **β** | **p-value unadjusted/ adjusted** | **β** | **p-value unadjusted/ adjusted** | **β** | **p-value unadjusted/ adjusted** |
| **Nutrition** | | | | | | | | | | | | |
| Duration of PN (y) | -0.55 | 0.669/0.807 | 0.16 | 0.817/0.844 | 0.07 | 0.417/0.807 | 0.06 | 0.911/0.911 | 0.11 | 0.295/0.729 | -0.26 | 0.094/0.618 |
| Type of nutrition | | | | | | | | | | | | |
| *PN only* | NA | 0.069/0.143 | NA | 0.369/0.440 | NA | 0.065/0.143 | NA | 0.054/0.143 | NA | 0.065/0.143 | NA | 0.002/**0.016** |
| *PN+tube feeding* | -3.48 | | 1.29 | | 0.36 | | 0.18 | | 0.50 | | 0.07 | |
| *PN+oral nutrition±tubefeeding* | -0.38 | | 0.355 | | 0.08 | | 0.70 | | -0.06 | | 0.37 | |
| *Tubefeeding/oral nutrition* | -18.4 | | 7.70 | | 1.46 | | 7.88 | | 1.58 | | 1.24 | |
| PN dependency (% of total energy intake) | 0.17 | 0.017/0.106 | -0.066 | 0.133/0.242 | -0.01 | 0.024/0.119 | -0.06 | 0.059/0.174 | -0.01 | 0.031/0.119 | -0.01 | 0.003/**0.049** |
| Calories of PN divided by REE (%) | 8.87 | 0.057/0.227 | -2.86 | 0.317/0.393 | -0.56 | 0.115/0.265 | -3.33 | 0.085/0.241 | -0.57 | 0.171/0.298 | -0.38 | 0.068/0.227 |
| Oral nutrition (no/yes) | -1.78 | 0.699/0.774 | 1.21 | 0.670/0.769 | 0.28 | 0.440/0.593 | 2.03 | 0.294/0.492 | 0.13 | 0.746/0.797 | 0.35 | 0.083/0.427 |
| Oral/enteral fiber intake per kg (g/kg) | -24.7 | 0.100/0.259 | 18.7 | 0.031/0.158 | 1.92 | 0.085/0.251 | 5.78 | 0.381/0.651 | 2.71 | 0.036/0.161 | 0.58 | 0.379/0.651 |
| Tube feeding (no/yes) | -3.88 | 0.480/0.640 | 2.06 | 0.516/0.640 | 0.45 | 0.262/0.527 | 0.55 | 0.804/0.859 | 0.54 | 0.260/0.527 | 0.10 | 0.707/0.811 |
| Tube feeding type | | | | | | | | | | | | |
| *Polymeric* | NA | 0.949/0.990 | NA | 0.857/0.990 | NA | 0.838/0.990 | NA | 0.751/0.990 | NA | 0.848/0.990 | NA | 0.571/0.931 |
| *Semi-elemental* | 2.68 | | -3.27 | | -0.35 | | -2.48 | | -0.49 | | -0.39 | |
| *Elemental* | -2.33 | | -3.81 | | 0.25 | | 2.12 | | 0.17 | | 1.47 | |
| Mode of tube feeding | | | | | | | | | | | | |
| *Continuous* | NA | 0.530/0.876 | NA | 0.795/0.957 | NA | 0.899/0.957 | NA | 0.267/0.786 | NA | 0.926/0.957 | NA | 0.357/0.786 |
| *Bolus* | -5.27 | | 1.64 | | 0.28 | | 3.17 | | 0.29 | | 0.55 | |
| *Both* | -12.5 | | 3.96 | | 0.14 | | 6.80 | | 0.22 | | -0.01 | |

| | | | | | | | | | | | | |
|---|---|---|---|---|---|---|---|---|---|---|---|---|
| **Gastro-intestinal characteristics** | | | | | | | | | | | | |
| Whole bowel in situ (no/yes) | -1.31 | 0.862/0.950 | 0.36 | 0.926/0.957 | 0.09 | 0.861/0.950 | 2.51 | 0.385/0.615 | -0.24 | 0.694/0.906 | 0.02 | 0.985/0.985 |
| Remaining small bowel length (cm) | 0.10 | 0.481/0.842 | -0.05 | 0.496/0.842 | -0.10 | 0.292/0.796 | -0.01 | 0.880/0.989 | -0.01 | 0.563/0.872 | 0.00 | 0.989/0.989 |
| Ileocecal valve in situ (no/yes) | -0.71 | 0.923/0.950 | -0.31 | 0.937/0.950 | 0.18 | 0.710/0.852 | 2.03 | 0.476/0.771 | -0.28 | 0.638/0.824 | 1.56 | 0.058/0.180 |
| Partial or total colectomy (no/yes) | 7.20 | 0.288/0.461 | -1.88 | 0.593/0.716 | -0.69 | 0.097/0.376 | -2.75 | 0.293/0.461 | -0.67 | 0.204/0.460 | -0.96 | 0.276/0.461 |
| **Growth** | | | | | | | | | | | | |
| BMI SDS | -2.00 | 0.460/0.852 | 0.88 | 0.615/0.852 | -0.10 | 0.666/0.852 | 0.02 | 0.983/0.983 | -0.04 | 0.876/0.970 | 0.01 | 0.962/0.983 |
| Height-for-age SDS < -2 (no/yes) | -13.2 | 0.322/0.704 | 3.65 | 0.600/0.750 | 1.23 | 0.154/0.478 | 6.68 | 0.194/0.502 | 1.20 | 0.260/0.619 | 1.26 | 0.454/0.704 |
| Growing outside target height range (no/yes) | -6.91 | 0.608/0.797 | 0.89 | 0.899/0.929 | 0.60 | 0.458/0.797 | 4.37 | 0.394/0.797 | 0.55 | 0.593/0.797 | 1.01 | 0.566/0.797 |
| **Medication use (no/yes)** | | | | | | | | | | | | |
| Proton pump inhibitor | 16.5 | 0.008/**0.035** | -8.98 | 0.004/**0.025** | -1.20 | 0.002/**0.016** | -2.47 | 0.340/0.499 | -1.67 | <0.001/**0.007** | -0.34 | 0.226/0.427 |
| Motility agents | 15.0 | 0.019/**0.063** | -7.72 | 0.024/**0.068** | -0.89 | 0.035/**0.081** | -5.34 | 0.037/**0.081** | -1.36 | 0.007/**0.039** | -0.18 | 0.668/0.828 |
| Cholestyramine | -6.99 | 0.206/0.426 | 1.59 | 0.604/0.693 | 0.31 | 0.480/0.620 | 0.81 | 0.728/0.806 | 0.64 | 0.199/0.426 | 1.33 | <0.001/**<0.001** |
| Ursochol | 15.6 | 0.053/0.150 | -7.04 | 0.130/0.288 | -1.37 | 0.024/0.083 | -2.73 | 0.414/0.755 | -1.65 | 0.020/**0.083** | -2.12 | <0.001/**<0.001** |
| Treatment of bacterial overgrowth | -3.40 | 0.723/0.833 | 3.65 | 0.461/0.696 | -0.25 | 0.693/0.833 | -4.50 | 0.217/0.597 | 0.10 | 0.901/0.931 | -0.90 | 0.458/0.696 |
| Antibiotics at sample* | 13.0 | 0.003/**0.019** | -7.37 | 0.004/**0.020** | -0.75 | 0.024/**0.069** | -4.39 | 0.018/**0.056** | -1.10 | 0.004/**<0.001** | -0.32 | 0.123/0.238 |
| Antibiotics between samples | 14.0 | <0.001/**0.001** | -7.25 | 0.003/**0.013** | -1.19 | <0.001/**0.001** | -4.60 | 0.005/**0.018** | -1.43 | <0.001/**<0.001** | -0.41 | 0.010/**0.028** |
| **Line sepsis** (no/yes)** | 14.8 | 0.007/**0.071** | -5.07 | 0.143/0.371 | -1.15 | 0.010/0.078 | -4.08 | 0.084/0.344 | -1.39 | 0.005/**0.071** | -0.72 | 0.002/**0.067** |

**Supplementary Table 8 continued.** General linear mixed model for clinical variables and % of short-chain fatty acids and branched-chain fatty acids

| **%** | **ic6** | | **c6** | | **c7** | | **c8** | |
|---|---|---|---|---|---|---|---|---|
| | **β** | **p-value unadjusted/ adjusted** | **β** | **p-value unadjusted/ adjusted** | **β** | **p-value unadjusted/ adjusted** | **β** | **p-value unadjusted/ adjusted** |
| **Nutrition** | | | | | | | | |
| Duration of PN (y) | -0.01 | 0.717/0.807 | -0.01 | 0.651/0.807 | 0.09 | 0.120/0.618 | 0.26 | 0.385/0.676 |
| Type of nutrition | | | | | | | | |
| *PN only* | NA | 0.757/0.757 | NA | 0.689/0.712 | NA | 0.262/0.338 | NA | 0.399/0.283 |
| *PN+tube feeding* | -0.04 | | 0.07 | | 0.45 | | 2.44 | |
| *PN+oral nutrition±tubefeeding* | 0.03 | | -0.01 | | -0.18 | | 0.11 | |
| *Tubefeeding/oral nutrition* | -0.08 | | 0.08 | | -0.12 | | 0.08 | |
| PN dependency (% of total energy intake) | 0.00 | 0.842/0.877 | -0.01 | 0.075/0.175 | 0.00 | 0.905/0.905 | -0.02 | 0.234/0.242 |
| Calories of PN divided by REE (%) | 0.02 | 0.714/0.738 | -0.11 | 0.072/0.227 | 0.11 | 0.674/0.720 | -1.64 | 0.213/0.287 |
| Oral nutrition (no/yes) | 0.00 | 0.998/0.998 | -0.03 | 0.649/0.769 | -0.41 | 0.141/0.463 | -1.11 | 0.404/0.574 |
| Oral/enteral fiber intake per kg (g/kg) | -0.02 | 0.894/0.920 | -0.12 | 0.519/0.699 | -0.61 | 0.474/0.668 | -2.21 | 0.609/0.764 |
| Tube feeding (no/yes) | -0.05 | 0.440/0.640 | 0.07 | 0.321/0.586 | 0.24 | 0.404/0.640 | 1.61 | 0.258/0.527 |
| Tube feeding type | | | | | | | | |
| *Polymeric* | NA | 0.829/0.990 | NA | 0.251/0.698 | NA | 0.916/0.990 | NA | 0.379/0.781 |
| *Semi-elemental* | -0.07 | | -0.01 | | 0.13 | | 4.09 | |
| *Elemental* | -0.03 | | 0.33 | | 0.30 | | -0.22 | |
| Mode of tube feeding | | | | | | | | |
| *Continuous* | NA | 0.701/0.945 | NA | 0.034/0.527 | NA | 0.584/0.876 | NA | 0.366/0.786 |
| *Bolus* | -0.03 | | -0.31 | | 0.25 | | -3.17 | |
| *Both* | 0.05 | | -0.23 | | -0.28 | | -3.27 | |
| **Gastro-intestinal characteristics** | | | | | | | | |
| Whole bowel in situ (no/yes) | 0.02 | 0.741/0.919 | -0.04 | 0.701/0.906 | -0.26 | 0.410/0.615 | -1.92 | 0.255/0.514 |

| | | | | | | | | |
|---|---|---|---|---|---|---|---|---|
| Remaining small bowel length (cm) | -0.01 | 0.212/0.795 | -0.01 | 0.117/0.605 | 0.01 | 0.334/0.796 | -0.05 | 0.085/0.605 |
| Ileocecal valve in situ (no/yes) | 0.14 | 0.055/0.180 | 0.06 | 0.569/0.802 | -0.28 | 0.424/0.771 | -3.61 | 0.032/0.140 |
| Partial or total colectomy (no/yes) | -0.12 | 0.026/0.272 | -0.11 | 0.274/0.461 | 0.40 | 0.160/0.450 | -0.62 | 0.686/0.733 |
| **Growth** | | | | | | | | |
| BMI SDS | -0.02 | 0.590/0.852 | 0.02 | 0.629/0.852 | 0.11 | 0.505/0.852 | -0.60 | 0.443/0.852 |
| Height-for-age SDS < -2 (no/yes) | -0.06 | 0.653/0.750 | 0.29 | 0.096/0.331 | -0.15 | 0.806/0.843 | -1.32 | 0.671/0.750 |
| Growing outside target height range (no/yes) | -0.06 | 0.623/0.797 | 0.34 | 0.056/0.351 | 0.24 | 0.665/0.797 | -1.58 | 0.609/0.797 |
| **Medication use (no/yes)** | | | | | | | | |
| Proton pump inhibitor | 0.03 | 0.648/0.744 | -0.07 | 0.372/0.501 | 0.18 | 0.526/0.627 | -2.14 | 0.145/0.326 |
| Motility agents | -0.02 | 0.716/0.854 | 0.01 | 0.952/1.000 | 0.72 | 0.008/0.620 | -0.48 | 0.768/0.882 |
| Cholestyramine | -0.13 | 0.023/0.101 | 0.13 | 0.078/0.241 | 0.21 | 0.464/0.620 | 1.98 | 0.153/0.365 |
| Ursochol | 0.01 | 0.922/0.974 | -0.25 | 0.037/0.116 | -0.14 | 0.755/0.923 | -0.54 | 0.804/0.923 |
| Treatment of bacterial overgrowth | -0.12 | 0.167/0.597 | 0.11 | 0.417/0.696 | -0.12 | 0.772/0.850 | 6.86 | <0.001/**0.004** |
| Antibiotics at sample[1] | -0.01 | 0.818/0.967 | 0.02 | 0.723/0.934 | 0.52 | 0.045/**0.095** | 1.17 | 0.361/0.567 |
| Antibiotics between samples | -0.02 | 0.762/0.844 | 0.02 | 0.661/0.844 | 0.41 | 0.162/0.314 | 0.80 | 0.527/0.779 |
| **Line sepsis** (no/yes)[2] | -0.05 | 0.514/0.613 | -0.11 | 0.106/0.364 | 0.15 | 0.686/0.759 | -1.31 | 0.430/0.522 |

**Legend**: [1] Due to bacterial overgrowth, line sepsis or another cause. [2] with a range of two months before and 2 months after sample collection.
A positive beta coefficient means that the two variables are positively associated, a negative coefficient means that they are negatively associated. For categorical variables the beta coefficients of all categories relative to the first category are mentioned; positive coefficients means that it is more positively associated with higher values of the response variable than the first category.
**Abbreviations:** BMI, body mass index; PN, parenteral nutrition; REE, resting energy expenditure; SDS, standard deviation score.

**Supplementary Table 9.** General linear mixed model for clinical variables and absolute values of short-chain fatty acids

| **Absolute values** | **c2** | | **c3** | | **ic4** | | **c4** | | **ic5** | | **c5** | |
|---|---|---|---|---|---|---|---|---|---|---|---|---|
| | **β** | **p-value unadjusted/ adjusted** | **β** | **p-value unadjusted/ adjusted** | **β** | **p-value unadjusted/ adjusted** | **β** | **p-value unadjusted/ adjusted** | **β** | **p-value unadjusted/ adjusted** | **β** | **p-value unadjusted/ adjusted** |
| **Nutrition** | | | | | | | | | | | | |
| Duration of PN (y) | -20.2 | 0.230/0.688 | -4.63 | 0.146/0.646 | -0.19 | 0.483/0.807 | -3.72 | 0.067/0.618 | -0.08 | 0.791/0.844 | -0.49 | 0.048/0.618 |
| Type of nutrition | | | | | | | | | | | | |
| *PN only* | NA | 0.191/0.283 | NA | 0.085/0.155 | NA | 0.006/**0.031** | NA | 0.005/**0.031** | NA | 0.015/**0.065** | NA | 0.006/**0.031** |
| *PN+tube feeding* | -15.1 | | -23.5 | | -1.24 | | -14.9 | | -0.84 | | -0.96 | |
| *PN+oral nutrition±tubefeeding* | -80.7 | | -5.63 | | -0.06 | | 3.95 | | 6.05 | | 0.50 | |
| *Tubefeeding/oral nutrition* | -36.4 | | 36.2 | | 5.44 | | 30.9 | | -0.85 | | 3.53 | |
| PN dependency (% of total energy intake) | 0.94 | 0.292/0.411 | -0.20 | 0.361/0.465 | -0.03 | 0.062/0.174 | -0.11 | 0.375/0.465 | -0.04 | 0.037/0.128 | -0.02 | 0.079/0.175 |
| Calories of PN divided by REE (%) | 77.2 | 0.179/0.298 | -15.5 | 0.270/0.363 | -2.25 | 0.059/0.227 | -8.57 | 0.309/0.393 | -2.62 | 0.052/0.227 | -1.25 | 0.128/0.265 |
| Oral nutrition (no/yes) | 15.14 | 0.792/0.818 | 18.6 | 0.272/0.492 | 2.27 | 0.080/0.427 | 16.5 | 0.075/0.427 | 2.07 | 0.182/0.463 | 1.08 | 0.186/0.463 |
| Oral/enteral fiber intake per kg (g/kg) | -126 | 0.399/0.651 | 98.7 | <0.001/**0.004** | 8.25 | 0.003/**0.030** | 32.3 | 0.089/0.251 | 11.1 | 0.002/**0.026** | 1.82 | 0.474/0.668 |
| Tube feeding (no/yes) | -121 | 0.072/0.386 | -12.2 | 0.426/0.640 | -0.42 | 0.754/0.835 | -10.5 | 0.249/0.527 | 0.22 | 0.885/0.887 | -0.95 | 0.342/0.589 |
| Tube feeding type | | | | | | | | | | | | |
| *Polymeric* | NA | 0.765/0.990 | NA | 0.121/0.552 | NA | 0.270/0.698 | NA | 0.191/0.591 | NA | 0.367/0.781 | NA | 0.012/0.103 |
| *Semi-elemental* | -32.5 | | -38.5 | | -3.21 | | -18.7 | | -3.90 | | -1.966 | |
| *Elemental* | 50.9 | | -30.0 | | -0.16 | | 8.12 | | -1.38 | | 2.20 | |
| Mode of tube feeding | | | | | | | | | | | | |
| *Continuous* | NA | 0.887/0.957 | NA | 0.161/0.786 | NA | 0.380/0.786 | NA | 0.235/0.786 | NA | 0.412/0.799 | NA | 0.593/0.876 |
| *Bolus* | 29.5 | | 14.0 | | 1.53 | | 8.10 | | 1.87 | | -0.58 | |

| | | | | | | | | | | | | |
|---|---|---|---|---|---|---|---|---|---|---|---|---|
| *Both* | 31.4 | | 31.3 | | 2.24 | | 20.0 | | 2.76 | | -1.37 | |
| **Gastro-intestinal characteristics** | | | | | | | | | | | | |
| Whole bowel in situ (no/yes) | 225 | 0.010/**0.044** | 35.6 | 0.035/0.109 | 3.27 | 0.023/**0.078** | 29.5 | 0.006/**0.031** | 1.91 | 0.251/0.514 | 4.39 | <0.001/**0.002** |
| Remaining small bowel length (cm) | 2.17 | 0.045/0.605 | -0.09 | 0.722/0.973 | -0.10 | 0.684/0.964 | 0.04 | 0.768/0.989 | -0.01 | 0.941/0.989 | 0.01 | 0.231/0.795 |
| Ileocecal valve in situ (no/yes) | 47.9 | 0.615/0.824 | 32.9 | 0.068/0.192 | 2.65 | 0.090/0.232 | 25.6 | 0.022/0.113 | 0.66 | 0.714/0.852 | 4.04 | 0.001/**0.021** |
| Partial or total colectomy (no/yes) | -47.0 | 0.602/0.716 | -8.07 | 0.624/0.716 | -1.60 | 0.248/0.461 | -11.6 | 0.297/0.461 | -0.95 | 0.530/0.716 | -2.48 | 0.048/0.372 |
| **Growth** | | | | | | | | | | | | |
| BMI SDS | 32.7 | 0.310/0.852 | 12.2 | 0.134/0.852 | 0.57 | 0.417/0.852 | 5.37 | 0.222/0.852 | 0.37 | 0.660/0.852 | 0.36 | 0.409/0.852 |
| Height-for-age SDS < -2 (no/yes) | 73.3 | 0.677/0.750 | 18.7 | 0.576/0.750 | 4.83 | 0.090/0.331 | 37.3 | 0.091/0.331 | 4.12 | 0.192/0.502 | 4.31 | 0.096/0.331 |
| Growing outside target height range (no/yes) | -36.3 | 0.832/0.929 | 0.91 | 0.977/0.977 | 2.04 | 0.406/0.797 | 25.8 | 0.235/0.797 | 1.16 | 0.668/0.797 | 2.76 | 0.280/0.797 |
| **Medication use (no/yes)** | | | | | | | | | | | | |
| Proton pump inhibitor | 99.7 | 0.147/0.326 | -16.9 | 0.280/0.434 | -2.54 | 0.053/0.151 | -3.59 | 0.707/0.782 | -3.29 | 0.021/**0.078** | -0.95 | 0.354/0.499 |
| Motility agents | -108 | 0.196/0.289 | -38.3 | 0.012/**0.048** | -3.64 | 0.004/**0.034** | -22.6 | 0.032/**0.081** | -4.49 | 0.001/**0.012** | -1.92 | 0.130/0.211 |
| Cholestyramine | -72.3 | 0.266/0.485 | -2.75 | 0.856/0.856 | 1.03 | 0.474/0.620 | -5.88 | 0.528/0.647 | 1.47 | 0.338/0.551 | 3.00 | 0.015/**0.095** |
| Ursochol | 95.9 | 0.343/0.664 | -15.2 | 0.516/0.836 | -3.59 | 0.086/0.223 | 1.45 | 0.917/0.974 | -3.19 | 0.175/0.362 | -5.57 | <0.001/**0.003** |
| Treatment of bacterial overgrowth | -141 | 0.246/0.597 | -16.0 | 0.494/0.696 | -2.42 | 0.222/0.597 | -23.9 | 0.114/0.597 | -1.91 | 0.382/0.696 | -2.41 | 0.182/0.597 |
| Antibiotics at sample[1] | -115 | 0.031/**0.081** | -46.5 | <0.001/**0.002** | -3.46 | 0.002/**0.016** | -18.73 | 0.017/**0.056** | -4.61 | <0.001/**0.002** | -2.23 | 0.004/**0.019** |
| Antibiotics between samples | -48.2 | 0.310/0.534 | -48.3 | <0.001/**0.001** | -4.66 | <0.001/**<0.001** | -18.8 | 0.006/**0.018** | -6.33 | <0.001/**<0.001** | -2.33 | <0.001/**0.002** |
| **Line sepsis** (no/yes)[2] | 112 | 0.089/0.344 | 4.17 | 0.818/0.846 | -1.60 | 0.313/0.555 | 5.51 | 0.588/0.675 | -1.95 | 0.295/0.555 | -1.54 | 0.125/0.371 |

**Supplementary Table 9 continued.** General linear mixed model for clinical variables and absolute values of short-chain fatty acids and branched-chain fatty acids

| **Absolute values** | **ic6** | | **c6** | | **c7** | | **c8** | | **total** | |
|---|---|---|---|---|---|---|---|---|---|---|
| | **β** | **p-value unadjusted/ adjusted** | **β** | **p-value unadjusted/ adjusted** | **β** | **p-value unadjusted/ adjusted** | **β** | **p-value unadjusted/ adjusted** | **β** | **p-value unadjusted/ adjusted** |
| **Nutrition** | | | | | | | | | | |
| Duration of PN (y) | -0.02 | 0.723/0.807 | -0.03 | 0.343/0.759 | 0.37 | 0.095/0.618 | 0.46 | 0.244/0.688 | -25.5 | 0.175/0.676 |
| Type of nutrition | | | | | | | | | | |
| *PN only* | NA | 0.110/0.190 | NA | 0.029/0.095 | NA | 0.616/0.658 | NA | 0.186/0.283 | NA | 0.080/0.154 |
| *PN+tube feeding* | -0.40 | | -0.11 | | 1.25 | | 4.00 | | -173 | |
| *PN+oral nutrition±tubefeeding* | 0.22 | | -0.10 | | -0.68 | | -0.70 | | -52.1 | |
| *Tubefeeding/oral nutrition* | -0.23 | | 0.65 | | 0.35 | | 0.85 | | 68.0 | |
| PN dependency (% of total energy intake) | 0.01 | 0.415/0.476 | -0.01 | 0.016/0.106 | -0.01 | 0.849/0.877 | -0.04 | 0.140/0.242 | 0.29 | 0.776/0.859 |
| Calories of PN divided by REE (%) | 0.13 | 0.535/0.624 | -0.38 | 0.021/0.213 | 0.08 | 0.943/0.943 | -2.69 | 0.148/0.287 | 37.1 | 0.573/0.634 |
| Oral nutrition (no/yes) | 0.27 | 0.194/0.463 | 0.22 | 0.183/0.463 | -0.97 | 0.407/0.574 | -2.39 | 0.216/0.478 | 70.7 | 0.302/0.492 |
| Oral/enteral fiber intake per kg (g/kg) | -0.15 | 0.676/0.788 | -0.46 | 0.385/0.651 | -1.49 | 0.686/0.788 | -1.79 | 0.765/0.847 | 16.85 | 0.920/0.920 |
| Tube feeding (no/yes) | -0.54 | 0.016/0.349 | 0.11 | 0.512/0.640 | 0.85 | 0.474/0.640 | 3.03 | 0.138/0.474 | -134 | 0.075/0.386 |
| Tube feeding type | | | | | | | | | | |
| *Polymeric* | NA | 0.531/0.915 | NA | 0.007/0.103 | NA | 0.990/0.990 | NA | 0.368/0.781 | NA | 0.403/0.781 |
| *Semi-elemental* | -0.22 | | -0.07 | | -0.22 | | 5.12 | | -135 | |
| *Elemental* | -0.10 | | 1.04 | | 0.23 | | -1.08 | | 13.9 | |
| Mode of tube feeding | | | | | | | | | | |

| | | | | | | | | | | |
|---|---|---|---|---|---|---|---|---|---|---|
| *Continuous* | NA | 0.120/0.786 | NA | 0.099/0.786 | NA | 0.540/0.876 | NA | 0.373/0.786 | NA | 0.518/0.876 |
| *Bolus* | 0.12 | | -0.50 | | 1.63 | | -3.35 | | 62.4 | |
| *Both* | 0.33 | | -0.34 | | -0.42 | | -4.40 | | 98.2 | |
| **Gastro-intestinal characteristics** | | | | | | | | | | |
| Whole bowel in situ (no/yes) | 0.62 | 0.006/**0.031** | 0.39 | 0.018/**0.069** | -0.77 | 0.553/0.780 | -3.82 | 0.079/0.204 | 273 | 0.004/**0.030** |
| Remaining small bowel length (cm) | 0.01 | 0.655/0.964 | -0.01 | 0.157/0.694 | 0.02 | 0.316/0.796 | -0.04 | 0.268/0.796 | 2.16 | 0.075/0.605 |
| Ileocecal valve in situ (no/yes) | 0.53 | 0.051/0.180 | 0.170 | 0.410/0.771 | -1.05 | 0.462/0.771 | -6.73 | 0.002/**0.037** | 110 | 0.300/0.715 |
| Partial or total colectomy (no/yes) | -0.39 | 0.068/0.376 | -0.27 | 0.085/0.376 | 1.78 | 0.119/0.410 | 1.31 | 0.522/0.716 | -59.8 | 0.560/0.716 |
| **Growth** | | | | | | | | | | |
| BMI SDS | 0.03 | 0.859/0.970 | 0.10 | 0.336/0.852 | 0.37 | 0.581/0.852 | -0.73 | 0.535/0.852 | 34.5 | 0.324/0.852 |
| Height-for-age SDS < -2 (no/yes) | -0.11 | 0.816/0.843 | 1.44 | <0.001/**<0.001** | 0.20 | 0.935/0.935 | -2.13 | 0.621/0.750 | 168 | 0.406/0.704 |
| Growing outside target height range (no/yes) | -0.27 | 0.535/0.797 | 1.02 | <0.001/**0.011** | 0.33 | 0.890/0.929 | -2.37 | 0.572/0.797 | 31.8 | 0.869/0.929 |
| **Medication use (no/yes)** | | | | | | | | | | |
| Proton pump inhibitor | 0.25 | 0.255/0.427 | -0.29 | 0.080/0.207 | 0.96 | 0.417/0.538 | -2.52 | 0.213/0.427 | 56.9 | 0.468/0.581 |
| Motility agents | -0.288 | 0.215/0.303 | -0.31 | 0.062/0.112 | 2.61 | 0.020/**0.063** | 0.42 | 0.848/0.939 | -167 | 0.074/0.128 |
| Cholestyramine | -0.38 | 0.071/0.241 | 0.31 | 0.093/0.241 | 1.20 | 0.313/0.540 | 4.60 | 0.012/**0.095** | -58.7 | 0.426/0.620 |
| Ursochol | -0.11 | 0.743/0.923 | -0.93 | 0.121/0.288 | -1.32 | 0.475/0.817 | -0.23 | 0.942/0.974 | 65.9 | 0.566/0.836 |
| Treatment of bacterial overgrowth | -0.47 | 0.135/0.597 | 0.07 | 0.795/0.850 | -0.91 | 0.593/0.800 | 8.79 | 0.001/**0.008** | -178 | 0.194/0.597 |
| Antibiotics at sample[1] | -0.02 | 0.936/0.967 | -0.03 | 0.874/0.967 | 1.63 | 0.134/0.244 | 2.31 | 0.204/0.351 | -152 | 0.013/**0.051** |
| Antibiotics between samples | 0.16 | 0.476/0.777 | -0.03 | 0.847/0.906 | 1.42 | 0.245/0.447 | 1.28 | 0.511/0.778 | -78.2 | 0.158/0.314 |
| **Line sepsis** (no/yes)[2] | 0.10 | 0.710/0.759 | -0.28 | 0.246/0.537 | 0.00 | 1.000/1.000 | -2.95 | 0.240/0.537 | 111 | 0.143/0.371 |

**Legend**: [1] Due to bacterial overgrowth, line sepsis or another cause. [2] with a range of two months before and 2 months after sample collection. A positive beta coefficient means that the two variables are positively associated, a negative coefficient means that they are negatively associated. For categorical variables the beta coefficients of all categories relative to the first category are mentioned; positive coefficients means that it is more positively associated with higher values of the response variable than the first category.

**Abbreviations:** BMI, body mass index; PN, parenteral nutrition; REE, resting energy expenditure; SDS, standard deviation score.

**Supplementary Table 10**. General linear mixed model for clinical variables and lactate and number of 16S rRNA gene copies

| | **L lactate** | | **D lactate** | | **Total lactate** | | **% D lactate** | | **Log of 16S rRNA gene copy number per g dry feces** | | **Log of 16S rRNA gene copy number per g wet feces** | |
|---|---|---|---|---|---|---|---|---|---|---|---|---|
| | **β** | **p-value unadjusted/ adjusted** | **β** | **p-value unadjusted/ adjusted** | **β** | **p-value unadjusted/ adjusted** | **β** | **p-value unadjusted/ adjusted** | **β** | **p-value unadjusted/ adjusted** | **β** | **p-value unadjusted/ adjusted** |
| **Nutrition** | | | | | | | | | | | | |
| Duration of PN (y) | 17.5 | 0.546/0.807 | 17.0 | 0.531/0.807 | -12.7 | 0.700/0.807 | -0.01 | 0.469/0.807 | 0.03 | 0.517/0.807 | 4.12 | 0.710/0.807 |
| Type of nutrition | | | | | | | | | | | | |
| *PN only* | NA | 0.238/0.320 | NA | 0.140/0.229 | NA | 0.067/0.143 | NA | 0.353/0.437 | NA | 0.031/**0.095** | NA | 0.542/0.600 |
| *PN+tube feeding* | -125 | | -284 | | 0.00 | | 0.03 | | 0.53 | | -62.0 | |
| *PN+oral nutrition±tubefeeding* | -148 | | -102 | | 329 | | 0.05 | | 0.56 | | -97.3 | |
| *Tubefeeding/oral nutrition* | -456 | | -333 | | -85.9 | | 0.08 | | 0.55 | | -115 | |
| PN dependency (% of total energy intake) | 5.31 | 0.008/**0.086** | 3.59 | 0.027/0.119 | 4.27 | 0.188/0.291 | -0.01 | 0.141/0.242 | -0.01 | 0.074/0.175 | 0.71 | 0.399/0.476 |
| Calories of PN divided by REE (%) | 308 | 0.016/0.213 | 204 | 0.051/0.227 | 220 | 0.186/0.298 | -0.04 | 0.116/0.265 | -0.21 | 0.250/0.352 | 34.0 | 0.544/0.624 |
| Oral nutrition (no/yes) | -185 | 0.191/0.463 | -62.8 | 0.622/0.769 | 146 | 0.399/0.574 | 0.04 | 0.139/0.463 | 0.20 | 0.300/0.492 | -72.2 | 0.232/0.480 |
| Oral/enteral fiber intake per kg (g/kg) | -488 | 0.189/0.451 | -371 | 0.212/0.469 | -1045 | 0.085/0.251 | 0.01 | 0.862/0.920 | 0.34 | 0.446/0.668 | -167 | 0.300/0.621 |
| Tube feeding (no/yes) | 25.8 | 0.887/0.887 | -274 | 0.023/0.349 | -365 | 0.113/0.474 | 0.03 | 0.239/0.527 | 0.37 | 0.041/0.349 | -64.3 | 0.272/0.527 |
| Tube feeding type | | | | | | | | | | | | |
| *Polymeric* | NA | 0.155/0.552 | NA | 0.980/0.990 | NA | 0.137/0.552 | NA | 0.979/0.990 | NA | 0.676/0.990 | NA | 0.476/0.869 |
| *Semi-elemental* | 551 | | 56.3 | | -312 | | 0.01 | | -0.26 | | 100 | |
| *Elemental* | 96.0 | | 46.2 | | -460 | | 0.01 | | -0.02 | | 6.78 | |
| Mode of tube feeding | | | | | | | | | | | | |
| *Continuous* | NA | 0.140/0.786 | NA | 0.775/0.957 | NA | 0.020/0.527 | NA | 0.679/0.945 | NA | 0.960/0.960 | NA | 0.369/0.786 |
| *Bolus* | 298 | | 106 | | 295 | | 0.03 | | -0.04 | | -73.2 | |
| *Both* | 301 | | 68.9 | | 554 | | 0.01 | | -0.07 | | -71.3 | |
| **Gastro-intestinal characteristics** | | | | | | | | | | | | |

| | | | | | | | | | | | | |
|---|---|---|---|---|---|---|---|---|---|---|---|---|
| Whole bowel in situ (no/yes) | -139 | 0.394/0.615 | 133 | 0.266/0.514 | -31.5 | 0.864/0.950 | -0.01 | 0.889/0.950 | 0.18 | 0.417/0.615 | 98.6 | 0.108/0.259 |
| Remaining small bowel length (cm) | -0.25 | 0.962/0.989 | 3.24 | 0.452/0.842 | 5.94 | 0.433/0.842 | 0.01 | 0.443/0.842 | -0.01 | 0.845/0.989 | -1.16 | 0.099/0.605 |
| Ileocecal valve in situ (no/yes) | -115 | 0.497/0.771 | 111 | 0.433/0.771 | 155 | 0.371/0.771 | -0.01 | 0.813/0.901 | -0.06 | 0.810/0.901 | 4.45 | 0.950/0.950 |
| Partial or total colectomy (no/yes) | 78.3 | 0.659/0.729 | -5.50 | 0.974/0.974 | 345 | 0.093/0.376 | 0.03 | 0.208/0.460 | -0.05 | 0.811/0.838 | -80.7 | 0.136/0.420 |
| **Growth** | | | | | | | | | | | | |
| BMI SDS | 66.3 | 0.502/0.852 | 123 | 0.116/0.852 | -91.7 | 0.423/0.852 | -0.01 | 0.687/0.852 | 0.01 | 0.960/0.983 | 42.0 | 0.212/0.852 |
| Height-for-age SDS < -2 (no/yes) | -222 | 0.349/0.704 | -77.6 | 0.665/0.750 | -379 | 0.057/0.331 | 0.04 | 0.447/0.704 | 0.32 | 0.423/0.704 | -69.6 | 0.587/0.750 |
| Growing outside target height range (no/yes) | -222 | 0.349/0.797 | -77.6 | 0.665/0.797 | -379 | 0.057/0.351 | 0.04 | 0.447/0.797 | 0.26 | 0.448/0.797 | -89.6 | 0.444/0.797 |
| **Medication use (no/yes)** | | | | | | | | | | | | |
| Proton pump inhibitor | 286 | 0.023/**0.078** | 294 | 0.007/**0.035** | 409 | 0.001/**0.015** | -0.01 | 0.732/0.782 | -0.22 | 0.244/0.427 | -1.75 | 0.976/0.991 |
| Motility agents | 461 | 0.003/**0.034** | 316 | 0.052/0.100 | 0.00 | 1.000/1.000 | -0.01 | 0.983/1.000 | -0.46 | 0.012/**0.048** | -69.3 | 0.232/0.312 |
| Cholestyramine | -216 | 0.086/0.241 | -261 | 0.023/0.101 | -428 | 0.005/**0.047** | 0.03 | 0.236/0.457 | 0.04 | 0.818/0.845 | 51.3 | 0.363/0.563 |
| Ursochol | 483 | 0.015/**0.080** | 666 | 0.024/**0.083** | 0.00 | 1.000/1.000 | 0.01 | 0.779/0.923 | -0.12 | 0.669/0.902 | -43.4 | 0.639/0.900 |
| Treatment of bacterial overgrowth | -239 | 0.278/0.615 | -221 | 0.189/0.597 | -348 | 0.084/0.597 | -0.01 | 0.723/0.833 | -0.01 | 0.988/0.988 | 91.9 | 0.250/0.597 |
| Antibiotics at sample[1] | 112 | 0.471/0.696 | -3.54 | 0.977/0.977 | -286 | 0.041/**0.095** | -0.01 | 0.977/0.934 | -0.16 | 0.366/0.567 | 14.7 | 0.787/0.967 |
| Antibiotics between samples | 41.5 | 0.752/0.844 | 14.0 | 0.911/0.941 | 354 | 0.028/**0.073** | 0.01 | 0.989/0.989 | -0.11 | 0.553/0.780 | -33.0 | 0.608/0.819 |
| **Line sepsis** (no/yes)[2] | 267 | 0.335/0.555 | 496 | 0.060/0.309 | 348 | 0.402/0.562 | 0.05 | 0.340/0.555 | 0.21 | 0.368/0.562 | -56.4 | 0.506/0.613 |

**Legend**: [1]Due to bacterial overgrowth, line sepsis or another cause. [2] with a range of two months before and 2 months after sample collection.
A positive beta coefficient means that the two variables are positively associated, a negative coefficient means that they are negatively associated. For categorical variables the beta coefficients of all categories relative to the first category are mentioned; positive coefficients means that it is more positively associated with higher values of the response variable than the first category.
**Abbreviations:** BMI, body mass index; PN, parenteral nutrition; REE, resting energy expenditure; SDS, standard deviation score.

**Supplementary Table 11.** Clinical variables associated with OTUs

| Variable | OTUs | Adjusted p-value | β coefficient*** | | |
|---|---|---|---|---|---|
| **Nutrition** | | | | | |
| Duration of PN (y) | No significant associations with OTUs | | | | |
| Type of nutrition (PN only (NA), PN+tube feeding, PN+oral nutrition±tubefeeding, tubefeeding/oral nutrition) | OTU_86_Genus_Parasutterella | <0.001 | -0.039 | -0.822 | 4.037 |
| | OTU_13_Genus_Bacteroides | <0.001 | -1.323 | 0.232 | 4.360 |
| | OTU_32_Genus_Bacteroides | <0.001 | -0.689 | -0.543 | 4.114 |
| | OTU_59_Genus_Barnesiella | <0.001 | -0.660 | 0.056 | 3.759 |
| | OTU_347_Genus_Lachnoclostridium | <0.001 | 0.287 | 0.275 | 3.069 |
| | OTU_88_Family_CFT112H7 | <0.001 | -0.370 | -0.943 | 3.720 |
| | OTU_128_Genus_Peptoclostridium | <0.001 | -0.029 | -0.819 | 3.599 |
| | OTU_102_Genus_Odoribacter | <0.001 | -0.601 | 0.341 | 3.787 |
| | OTU_49_Genus_Roseburia | 0.001 | -2.051 | -0.558 | 2.650 |
| | OTU_57_Genus_Subdoligranulum | 0.001 | -0.475 | -0.399 | 2.618 |
| | OTU_55_Genus_Blautia | 0.001 | -0.752 | -0.145 | 2.443 |
| | OTU_205_Genus_Alistipes | 0.001 | -0.554 | 0.073 | 2.574 |
| | OTU_63_Genus_Lachnoclostridium | 0.001 | -0.953 | -0.338 | 3.084 |
| | OTU_67_Genus_Collinsella | 0.001 | -1.356 | -0.973 | 1.981 |
| | OTU_268_Genus_Holdemanella | 0.002 | 0.575 | 0.052 | 2.509 |
| | OTU_23_Genus_Alistipes | 0.002 | -1.687 | 0.475 | 3.181 |
| | OTU_220_Family_Lachnospiraceae | 0.002 | -0.395 | 0.483 | 2.974 |
| | OTU_33_Genus_Pseudobutyrivibrio | 0.003 | -0.889 | -0.536 | 2.790 |
| | OTU_60_Genus_Anaerostipes | 0.004 | -0.569 | 0.273 | 2.853 |
| | OTU_108_Genus_Fusicatenibacter | 0.006 | -0.955 | 0.147 | 2.356 |
| | OTU_943_Class_Bacteroidetes VC2.1 Bac22 | 0.011 | 0.713 | 1.465 | 2.245 |
| | OTU_10_Family_Bacteroidales S24-7 group | 0.012 | 0.041 | 0.217 | 2.827 |
| | OTU_12_Genus_Bacteroides | 0.018 | -2.147 | 0.095 | 2.735 |
| | OTU_133_Genus_Bacteroides | 0.019 | -1.825 | 0.063 | 2.624 |
| | OTU_207_Genus_Allisonella | 0.020 | 0.135 | -0.219 | 2.207 |
| | OTU_230_Genus_Coprobacter | 0.021 | -0.629 | 0.689 | 1.610 |
| | OTU_226_Genus_Slackia | 0.021 | 0.174 | -0.178 | 2.172 |
| | OTU_28_Genus_Bacteroides | 0.021 | 0.472 | 2.489 | 4.141 |
| | OTU_155_Genus_Enterorhabdus | 0.023 | 0.133 | 0.322 | 2.297 |
| | OTU_82_Genus_Bacteroides | 0.023 | -0.770 | 1.046 | 1.829 |
| | OTU_116_Genus_Ruminococcaceae UCG-005 | 0.024 | -0.683 | -0.139 | 1.870 |
| | OTU_106_Genus_[Eubacterium] hallii group | 0.025 | -0.400 | 0.455 | 1.901 |
| | OTU_62_Genus_Parabacteroides | 0.028 | -0.966 | 1.488 | 1.518 |
| | OTU_209_Genus_Victivallis | 0.029 | 0.191 | 0.311 | 2.100 |

| | | | | | |
|---|---|---|---|---|---|
| | OTU_159_Class_Bacteroidetes VC2.1 Bac22 | 0.030 | -0.221 | 0.066 | 2.081 |
| | OTU_85_Genus_Lachnospiraceae NK4A136 group | 0.030 | -1.318 | -0.311 | 2.033 |
| | OTU_39_Genus_Alistipes | 0.030 | -1.002 | 1.391 | 1.989 |
| | OTU_213_Order_Gastranaerophilales | 0.030 | 0.225 | 0.322 | 1.859 |
| | OTU_99_Genus_Bacteroides | 0.030 | -0.586 | -0.472 | 2.204 |
| | OTU_43_Family_Lachnospiraceae | 0.030 | 0.669 | -0.622 | -2.032 |
| | OTU_191_Genus_Lachnospiraceae UCG-008 | 0.030 | -1.000 | -0.226 | 0.858 |
| | OTU_75_Family_Ruminococcaceae | 0.039 | -0.685 | 0.399 | 1.976 |
| | OTU_74_Genus_Ruminococcaceae UCG-002 | 0.039 | -0.406 | 0.137 | 2.699 |
| | OTU_44_Genus_[Eubacterium] coprostanoligenes group | 0.055 | -0.213 | 0.167 | 1.751 |
| | OTU_130_Genus_Acidaminococcus | 0.055 | 0.260 | 0.224 | 2.077 |
| | OTU_26_Genus_Blautia | 0.055 | -1.156 | -0.067 | 2.564 |
| | OTU_93_Genus_Blautia | 0.059 | -0.815 | 0.379 | 1.920 |
| | OTU_184_Genus_Anaerosporobacter | 0.059 | 0.432 | 1.246 | 0.431 |
| | OTU_19_Genus_Tyzzerella 4 | 0.061 | -3.313 | -1.131 | -1.395 |
| | OTU_1_Genus_Escherichia-Shigella | 0.065 | -1.521 | -0.886 | -4.733 |
| | OTU_196_Genus_Ruminococcaceae NK4A214 group | 0.065 | 0.229 | 0.330 | 1.324 |
| | OTU_96_Genus_Streptococcus | 0.068 | -1.460 | 0.335 | -1.359 |
| | OTU_41_Genus_Subdoligranulum | 0.070 | 0.062 | 0.158 | 2.403 |
| | OTU_89_Genus_Lachnospira | 0.070 | -1.673 | -1.202 | 0.999 |
| | OTU_42_Genus_Ruminococcus 2 | 0.071 | 0.248 | -0.185 | 2.284 |
| | OTU_48_Genus_Blautia | 0.071 | -0.542 | -0.204 | 1.374 |
| | OTU_78_Genus_Blautia | 0.072 | 0.391 | 1.314 | 1.330 |
| | OTU_14_Genus_Staphylococcus | 0.079 | -0.550 | -0.307 | -3.509 |
| | OTU_113_Genus_Lachnoclostridium | 0.083 | -1.459 | -0.317 | 1.293 |
| | OTU_154_Genus_Incertae Sedis | 0.085 | -0.072 | 0.241 | 1.436 |
| | OTU_29_Family_Lachnospiraceae | 0.088 | -2.486 | -1.142 | 0.479 |
| | OTU_114_Genus_Faecalibacterium | 0.098 | -0.547 | 0.113 | 1.334 |
| | OTU_194_Genus_Butyricimonas | 0.098 | -0.553 | -0.062 | 1.169 |
| | OTU_695_Genus_Bacteroides | 0.098 | -1.039 | 0.417 | 1.723 |
| | OTU_261_Genus_Desulfovibrio | 0.098 | -0.210 | 0.402 | 1.721 |
| | OTU_204_Genus_Ruminiclostridium | 0.098 | -0.743 | 0.038 | 1.508 |
| | OTU_94_Genus_Clostridium sensu stricto 1 | 0.098 | -1.738 | -1.118 | -1.070 |
| | OTU_61_Genus_Clostridium sensu stricto 1 | 0.099 | -1.716 | 0.015 | -2.036 |
| Calories of PN divided by REE (%) | OTU_86_Genus_Parasutterella | 0.001 | -2.627 | | |
| | OTU_88_Family_CFT112H7 | 0.001 | -2.768 | | |
| | OTU_128_Genus_Peptoclostridium | 0.001 | -2.539 | | |
| | OTU_347_Genus_Lachnoclostridium | 0.004 | -1.402 | | |
| | OTU_268_Genus_Holdemanella | 0.004 | -1.487 | | |

| | | | | | |
|---|---|---|---|---|---|
| | OTU_32_Genus_Bacteroides | 0.004 | -1.969 | | |
| | OTU_59_Genus_Barnesiella | 0.015 | -1.764 | | |
| | OTU_13_Genus_Bacteroides | 0.020 | -2.180 | | |
| | OTU_207_Genus_Allisonella | 0.022 | -1.344 | | |
| | OTU_226_Genus_Slackia | 0.022 | -1.310 | | |
| | OTU_7_Genus_Prevotella 9 | 0.022 | 1.361 | | |
| | OTU_44_Genus_[Eubacterium] coprostanoligenes group | 0.024 | -1.172 | | |
| | OTU_57_Genus_Subdoligranulum | 0.026 | -1.208 | | |
| | OTU_55_Genus_Blautia | 0.034 | -1.336 | | |
| | OTU_1_Genus_Escherichia-Shigella | 0.034 | 2.777 | | |
| | OTU_213_Order_Gastranaerophilales | 0.034 | -1.024 | | |
| | OTU_196_Genus_Ruminococcaceae NK4A214 group | 0.038 | -0.700 | | |
| | OTU_102_Genus_Odoribacter | 0.042 | -1.735 | | |
| | OTU_130_Genus_Acidaminococcus | 0.044 | -1.103 | | |
| | OTU_231_Genus_Leuconostoc | 0.044 | -1.504 | | |
| | OTU_10_Family_Bacteroidales S24-7 group | 0.047 | -1.426 | | |
| | OTU_60_Genus_Anaerostipes | 0.047 | -1.354 | | |
| | OTU_220_Family_Lachnospiraceae | 0.054 | -1.289 | | |
| | OTU_99_Genus_Bacteroides | 0.054 | -1.452 | | |
| | OTU_943_Class_Bacteroidetes VC2.1 Bac22 | 0.054 | -1.131 | | |
| | OTU_28_Genus_Bacteroides | 0.054 | -2.040 | | |
| | OTU_63_Genus_Lachnoclostridium | 0.059 | -1.385 | | |
| | OTU_209_Genus_Victivallis | 0.065 | -1.129 | | |
| | OTU_33_Genus_Pseudobutyrivibrio | 0.081 | -1.355 | | |
| | OTU_61_Genus_Clostridium sensu stricto 1 | 0.082 | 1.611 | | |
| | OTU_205_Genus_Alistipes | 0.084 | -1.129 | | |
| | OTU_155_Genus_Enterorhabdus | 0.087 | -1.237 | | |
| | OTU_90_Genus_Thalassospira | 0.092 | -0.900 | | |
| | OTU_72_Genus_Phascolarctobacterium | 0.099 | 0.946 | | |
| PN dependency (% of total energy intake) | OTU_347_Genus_Lachnoclostridium | 0.009 | -0.022 | | |
| | OTU_86_Genus_Parasutterella | 0.012 | -0.033 | | |
| | OTU_88_Family_CFT112H7 | 0.051 | -0.029 | | |
| | OTU_128_Genus_Peptoclostridium | 0.051 | -0.028 | | |
| | OTU_32_Genus_Bacteroides | 0.051 | -0.026 | | |
| | OTU_59_Genus_Barnesiella | 0.051 | -0.024 | | |
| | OTU_943_Class_Bacteroidetes VC2.1 Bac22 | 0.051 | -0.019 | | |
| | OTU_7_Genus_Prevotella 9 | 0.052 | 0.019 | | |
| | OTU_213_Order_Gastranaerophilales | 0.052 | -0.015 | | |
| | OTU_196_Genus_Ruminococcaceae NK4A214 group | 0.057 | -0.011 | | |

| | | | | | |
|---|---|---|---|---|---|
| | OTU_268_Genus_Holdemanella | 0.058 | -0.017 | | |
| | OTU_57_Genus_Subdoligranulum | 0.058 | -0.017 | | |
| | OTU_130_Genus_Acidaminococcus | 0.058 | -0.018 | | |
| | OTU_10_Family_Bacteroidales S24-7 group | 0.058 | -0.020 | | |
| | OTU_209_Genus_Victivallis | 0.058 | -0.016 | | |
| | OTU_155_Genus_Enterorhabdus | 0.058 | -0.018 | | |
| | OTU_13_Genus_Bacteroides | 0.070 | -0.026 | | |
| | OTU_207_Genus_Allisonella | 0.070 | -0.018 | | |
| | OTU_226_Genus_Slackia | 0.070 | -0.017 | | |
| | OTU_61_Genus_Clostridium sensu stricto 1 | 0.070 | 0.027 | | |
| | OTU_18_Genus_Lactobacillus | 0.070 | -0.029 | | |
| | OTU_220_Family_Lachnospiraceae | 0.074 | -0.019 | | |
| | OTU_44_Genus_[Eubacterium] coprostanoligenes group | 0.075 | -0.015 | | |
| | OTU_55_Genus_Blautia | 0.075 | -0.018 | | |
| | OTU_1_Genus_Escherichia-Shigella | 0.075 | 0.037 | | |
| | OTU_102_Genus_Odoribacter | 0.075 | -0.023 | | |
| | OTU_42_Genus_Ruminococcus 2 | 0.075 | -0.019 | | |
| | OTU_22_Genus_Lachnoclostridium | 0.091 | 0.024 | | |
| | OTU_159_Class_Bacteroidetes VC2.1 Bac22 | 0.095 | -0.016 | | |
| | OTU_90_Genus_Thalassospira | 0.096 | -0.015 | | |
| | OTU_14_Genus_Staphylococcus | 0.096 | 0.026 | | |
| Oral nutrition (no/yes) | OTU_62_Genus_Parabacteroides | 0.003 | 2.591 | | |
| | OTU_13_Genus_Bacteroides | 0.015 | 2.465 | | |
| | OTU_220_Family_Lachnospiraceae | 0.032 | 1.699 | | |
| | OTU_28_Genus_Bacteroides | 0.032 | 2.861 | | |
| | OTU_59_Genus_Barnesiella | 0.047 | 1.777 | | |
| | OTU_55_Genus_Blautia | 0.048 | 1.354 | | |
| | OTU_102_Genus_Odoribacter | 0.048 | 1.913 | | |
| | OTU_23_Genus_Alistipes | 0.048 | 2.431 | | |
| | OTU_75_Family_Ruminococcaceae | 0.048 | 1.519 | | |
| | OTU_133_Genus_Bacteroides | 0.048 | 2.304 | | |
| | OTU_78_Genus_Blautia | 0.058 | 1.058 | | |
| | OTU_49_Genus_Roseburia | 0.058 | 2.054 | | |
| | OTU_82_Genus_Bacteroides | 0.058 | 1.459 | | |
| | OTU_12_Genus_Bacteroides | 0.061 | 2.382 | | |
| | OTU_347_Genus_Lachnoclostridium | 0.068 | 1.090 | | |
| | OTU_943_Class_Bacteroidetes VC2.1 Bac22 | 0.068 | 1.146 | | |
| | OTU_60_Genus_Anaerostipes | 0.068 | 1.426 | | |
| | OTU_106_Genus_[Eubacterium] hallii group | 0.068 | 1.135 | | |

| | | | | | |
|---|---|---|---|---|---|
| | OTU_108_Genus_Fusicatenibacter | 0.068 | 1.479 | | |
| | OTU_39_Genus_Alistipes | 0.068 | 1.887 | | |
| | OTU_230_Genus_Coprobacter | 0.068 | 1.120 | | |
| | OTU_191_Genus_Lachnospiraceae UCG-008 | 0.068 | 0.938 | | |
| | OTU_181_Genus_Dielma | 0.068 | 0.954 | | |
| | OTU_695_Genus_Bacteroides | 0.073 | 1.576 | | |
| | OTU_113_Genus_Lachnoclostridium | 0.073 | 1.545 | | |
| | OTU_32_Genus_Bacteroides | 0.076 | 1.474 | | |
| | OTU_63_Genus_Lachnoclostridium | 0.086 | 1.384 | | |
| | OTU_80_Family_Lachnospiraceae | 0.086 | 1.556 | | |
| | OTU_43_Family_Lachnospiraceae | 0.089 | -1.162 | | |
| | OTU_171_Genus_Staphylococcus | 0.094 | 1.077 | | |
| Oral/enteral fiber intake per kg | OTU_99_Genus_Bacteroides | <0.001 | 8.300 | | |
| | OTU_207_Genus_Allisonella | <0.001 | 6.414 | | |
| | OTU_226_Genus_Slackia | <0.001 | 6.303 | | |
| | OTU_128_Genus_Peptoclostridium | <0.001 | 8.984 | | |
| | OTU_86_Genus_Parasutterella | <0.001 | 8.507 | | |
| | OTU_130_Genus_Acidaminococcus | <0.001 | 5.682 | | |
| | OTU_88_Family_CFT112H7 | <0.001 | 9.088 | | |
| | OTU_34_Genus_Veillonella | <0.001 | 10.627 | | |
| | OTU_90_Genus_Thalassospira | <0.001 | 4.698 | | |
| | OTU_268_Genus_Holdemanella | <0.001 | 5.162 | | |
| | OTU_261_Genus_Desulfovibrio | <0.001 | 4.263 | | |
| | OTU_145_Genus_Bacteroides | 0.001 | 4.980 | | |
| | OTU_32_Genus_Bacteroides | 0.001 | 6.469 | | |
| | OTU_943_Class_Bacteroidetes VC2.1 Bac22 | 0.001 | 3.751 | | |
| | OTU_59_Genus_Barnesiella | 0.002 | 5.800 | | |
| | OTU_347_Genus_Lachnoclostridium | 0.004 | 4.316 | | |
| | OTU_101_Genus_Thalassospira | 0.005 | 3.647 | | |
| | OTU_102_Genus_Odoribacter | 0.014 | 4.483 | | |
| | OTU_13_Genus_Bacteroides | 0.018 | 5.070 | | |
| | OTU_57_Genus_Subdoligranulum | 0.018 | 3.786 | | |
| | OTU_156_Genus_Ruminiclostridium 9 | 0.046 | 3.251 | | |
| | OTU_220_Family_Lachnospiraceae | 0.061 | 4.044 | | |
| Tube feeding (no/yes) | No significant associations with OTUs | | | | |
| Tube feeding type (polymeric, semi-elemental, elemental) | OTU_34_Genus_Veillonella | 0.006 | -0.013 | 3.921 | |
| | OTU_56_Genus_Alistipes | 0.006 | -0.564 | 2.826 | |
| | OTU_159_Class_Bacteroidetes VC2.1 Bac22 | 0.006 | -0.422 | 2.771 | |
| | OTU_80_Family_Lachnospiraceae | 0.006 | -0.490 | 2.573 | |

| | | | | | |
|---|---|---|---|---|---|
| | OTU_155_Genus_Enterorhabdus | 0.006 | 0.087 | 3.358 | |
| | OTU_230_Genus_Coprobacter | 0.006 | -4.501 | -6.141 | |
| | OTU_209_Genus_Victivallis | 0.012 | -0.482 | 2.471 | |
| | OTU_10_Family_Bacteroidales S24-7 group | 0.016 | -0.611 | 3.237 | |
| | OTU_213_Order_Gastranaerophilales | 0.017 | -0.425 | 2.029 | |
| | OTU_116_Genus_Ruminococcaceae UCG-005 | 0.021 | -0.806 | 2.434 | |
| | OTU_88_Family_CFT112H7 | 0.023 | 0.148 | 1.981 | |
| | OTU_99_Genus_Bacteroides | 0.023 | 0.712 | 3.450 | |
| | OTU_128_Genus_Peptoclostridium | 0.023 | -0.027 | 2.805 | |
| | OTU_152_Genus_Christensenellaceae R-7 group | 0.023 | -0.221 | 1.982 | |
| | OTU_41_Genus_Subdoligranulum | 0.023 | 0.265 | 2.541 | |
| | OTU_114_Genus_Faecalibacterium | 0.023 | -3.254 | -3.878 | |
| | OTU_82_Genus_Bacteroides | 0.023 | -3.702 | -4.499 | |
| | OTU_194_Genus_Butyricimonas | 0.023 | 0.013 | 2.707 | |
| | OTU_65_Genus_Ruminococcaceae UCG-002 | 0.023 | -0.205 | 2.222 | |
| | OTU_154_Genus_Incertae Sedis | 0.023 | -3.786 | -4.561 | |
| | OTU_140_Genus_Ruminococcaceae NK4A214 group | 0.023 | -1.457 | 1.320 | |
| | OTU_205_Genus_Alistipes | 0.023 | 0.123 | 2.794 | |
| | OTU_268_Genus_Holdemanella | 0.027 | -2.103 | -2.938 | |
| | OTU_31_Genus_Faecalibacterium | 0.030 | 0.180 | 3.159 | |
| | OTU_695_Genus_Bacteroides | 0.031 | -1.588 | 1.343 | |
| | OTU_207_Genus_Allisonella | 0.037 | -2.332 | -2.782 | |
| | OTU_226_Genus_Slackia | 0.038 | -1.399 | 2.612 | |
| | OTU_231_Genus_Leuconostoc | 0.038 | -2.282 | -2.713 | |
| | OTU_86_Genus_Parasutterella | 0.040 | -3.292 | -3.854 | |
| | OTU_78_Genus_Blautia | 0.040 | -0.192 | 1.619 | |
| | OTU_44_Genus_[Eubacterium] coprostanoligenes group | 0.040 | -0.427 | 1.774 | |
| | OTU_305_Genus_Lachnoanaerobaculum | 0.081 | 2.069 | -0.624 | |
| | OTU_131_Genus_Blautia | 0.086 | -0.457 | 1.625 | |
| | OTU_28_Genus_Bacteroides | 0.086 | -3.392 | 1.233 | |
| | OTU_261_Genus_Desulfovibrio | 0.089 | -2.144 | 0.441 | |
| | OTU_49_Genus_Roseburia | 0.089 | -1.745 | -2.045 | |
| | OTU_83_Genus_Ruminococcus 1 | 0.093 | -0.108 | 2.232 | |
| | OTU_145_Genus_Bacteroides | 0.093 | -1.688 | -2.763 | |
| | OTU_156_Genus_Ruminiclostridium 9 | 0.094 | -1.691 | -2.165 | |
| | OTU_85_Genus_Lachnospiraceae NK4A136 group | 0.095 | 0.459 | 2.486 | |
| | OTU_130_Genus_Acidaminococcus | 0.098 | -0.554 | 2.102 | |
| | OTU_62_Genus_Parabacteroides | 0.098 | -1.913 | -2.148 | |
| | OTU_73_Genus_Coprococcus 3 | 0.099 | 1.306 | 0.197 | |

| | | | | | |
|---|---|---|---|---|---|
| | OTU_23_Genus_Alistipes | 0.099 | -2.307 | 0.361 | |
| | OTU_133_Genus_Bacteroides | 0.099 | -2.628 | 0.936 | |
| | OTU_72_Genus_Phascolarctobacterium | 0.099 | 0.046 | 2.304 | |
| Mode of tube feeding (continuous, bolus, both) | OTU_261_Genus_Desulfovibrio | <0.001 | 5.055 | -0.236 | |
| | OTU_145_Genus_Bacteroides | <0.001 | 6.643 | 0.466 | |
| | OTU_156_Genus_Ruminiclostridium 9 | <0.001 | 4.449 | -0.888 | |
| | OTU_87_Genus_[Eubacterium] coprostanoligenes group | 0.005 | 2.579 | -0.422 | |
| | OTU_244_Genus_Blautia | 0.011 | 3.717 | -0.350 | |
| | OTU_34_Genus_Veillonella | 0.057 | 2.630 | 4.133 | |
| | OTU_90_Genus_Thalassospira | 0.068 | 0.295 | 2.167 | |
| | OTU_88_Family_CFT112H7 | 0.083 | 6.236 | 3.487 | |
| | OTU_99_Genus_Bacteroides | 0.095 | 5.544 | 1.837 | |
| **Gastro-intestinal characteristics** | | | | | |
| Whole bowel in situ (no/yes) | OTU_56_Genus_Alistipes | <0.001 | 3.128 | | |
| | OTU_80_Family_Lachnospiraceae | <0.001 | 3.102 | | |
| | OTU_58_Genus_Parabacteroides | <0.001 | 3.013 | | |
| | OTU_84_Genus_Lachnoclostridium | <0.001 | 4.302 | | |
| | OTU_12_Genus_Bacteroides | 0.002 | 3.470 | | |
| | OTU_152_Genus_Christensenellaceae R-7 group | 0.002 | 1.752 | | |
| | OTU_121_Genus_Dorea | 0.003 | 2.351 | | |
| | OTU_98_Genus_Lachnospiraceae NK4A136 group | 0.003 | 1.811 | | |
| | OTU_695_Genus_Bacteroides | 0.004 | 2.192 | | |
| | OTU_199_Genus_Anaerostipes | 0.004 | 1.895 | | |
| | OTU_75_Family_Ruminococcaceae | 0.004 | 1.833 | | |
| | OTU_93_Genus_Blautia | 0.004 | 2.117 | | |
| | OTU_133_Genus_Bacteroides | 0.004 | 2.740 | | |
| | OTU_113_Genus_Lachnoclostridium | 0.005 | 2.099 | | |
| | OTU_204_Genus_Ruminiclostridium | 0.005 | 1.788 | | |
| | OTU_62_Genus_Parabacteroides | 0.007 | 2.075 | | |
| | OTU_49_Genus_Roseburia | 0.007 | 2.687 | | |
| | OTU_30_Genus_Hungatella | 0.008 | 3.897 | | |
| | OTU_45_Genus_Lachnoclostridium | 0.015 | 3.275 | | |
| | OTU_97_Genus_Dorea | 0.017 | 1.381 | | |
| | OTU_55_Genus_Blautia | 0.019 | 1.487 | | |
| | OTU_23_Genus_Alistipes | 0.019 | 2.584 | | |
| | OTU_85_Genus_Lachnospiraceae NK4A136 group | 0.020 | 1.893 | | |
| | OTU_92_Genus_Bilophila | 0.022 | 1.993 | | |
| | OTU_171_Genus_Staphylococcus | 0.024 | 1.375 | | |
| | OTU_205_Genus_Alistipes | 0.027 | 1.572 | | |

| | | | | | |
|---|---|---|---|---|---|
| | OTU_191_Genus_Lachnospiraceae UCG-008 | 0.034 | 0.996 | | |
| | OTU_15_Genus_Tyzzerella | 0.038 | 1.559 | | |
| | OTU_5_Genus_Akkermansia | 0.038 | 1.633 | | |
| | OTU_126_Genus_Eggerthella | 0.038 | 2.785 | | |
| | OTU_6_Genus_Haemophilus | 0.048 | 1.813 | | |
| | OTU_230_Genus_Coprobacter | 0.048 | 1.544 | | |
| | OTU_19_Genus_Tyzzerella 4 | 0.049 | 2.933 | | |
| | OTU_215_Genus_Erysipelatoclostridium | 0.049 | 1.641 | | |
| | OTU_82_Genus_Bacteroides | 0.058 | 1.966 | | |
| | OTU_83_Genus_Ruminococcus 1 | 0.058 | 1.193 | | |
| | OTU_16_Genus_Lachnoclostridium | 0.069 | 3.204 | | |
| | OTU_22_Genus_Lachnoclostridium | 0.074 | 3.698 | | |
| | OTU_71_Genus_Blautia | 0.074 | 1.947 | | |
| | OTU_201_Family_Lachnospiraceae | 0.081 | 1.168 | | |
| | OTU_52_Genus_Peptoclostridium | 0.091 | 1.994 | | |
| | OTU_94_Genus_Clostridium sensu stricto 1 | 0.098 | 1.810 | | |
| Ileocecal valve in situ (no/yes) | OTU_135_Genus_Lachnoclostridium | 0.023 | 1.903 | | |
| | OTU_152_Genus_Christensenellaceae R-7 group | 0.030 | 1.726 | | |
| | OTU_93_Genus_Blautia | 0.030 | 2.151 | | |
| | OTU_92_Genus_Bilophila | 0.030 | 2.467 | | |
| | OTU_126_Genus_Eggerthella | 0.030 | 3.025 | | |
| | OTU_69_Family_Ruminococcaceae | 0.030 | 2.490 | | |
| | OTU_144_Genus_Ruminococcaceae UCG-004 | 0.030 | 1.743 | | |
| | OTU_45_Genus_Lachnoclostridium | 0.030 | 3.647 | | |
| | OTU_49_Genus_Roseburia | 0.030 | 2.738 | | |
| | OTU_82_Genus_Bacteroides | 0.031 | 2.185 | | |
| | OTU_85_Genus_Lachnospiraceae NK4A136 group | 0.031 | 2.198 | | |
| | OTU_12_Genus_Bacteroides | 0.031 | 3.168 | | |
| | OTU_84_Genus_Lachnoclostridium | 0.031 | 3.470 | | |
| | OTU_15_Genus_Tyzzerella | 0.031 | 1.941 | | |
| | OTU_208_Genus_Ruminiclostridium | 0.031 | 1.856 | | |
| | OTU_23_Genus_Alistipes | 0.031 | 2.753 | | |
| | OTU_133_Genus_Bacteroides | 0.041 | 2.683 | | |
| | OTU_62_Genus_Parabacteroides | 0.046 | 2.078 | | |
| | OTU_199_Genus_Anaerostipes | 0.049 | 1.624 | | |
| | OTU_244_Genus_Blautia | 0.049 | 1.845 | | |
| | OTU_33_Genus_Pseudobutyrivibrio | 0.051 | 1.887 | | |
| | OTU_56_Genus_Alistipes | 0.052 | 2.348 | | |
| | OTU_695_Genus_Bacteroides | 0.058 | 1.951 | | |

| | | | | | |
|---|---|---|---|---|---|
| | OTU_58_Genus_Parabacteroides | 0.059 | 2.364 | | |
| | OTU_74_Genus_Ruminococcaceae UCG-002 | 0.065 | 1.910 | | |
| | OTU_26_Genus_Blautia | 0.065 | 2.203 | | |
| | OTU_191_Genus_Lachnospiraceae UCG-008 | 0.068 | 1.061 | | |
| | OTU_98_Genus_Lachnospiraceae NK4A136 group | 0.072 | 1.551 | | |
| | OTU_13_Genus_Bacteroides | 0.073 | 2.189 | | |
| | OTU_71_Genus_Blautia | 0.091 | 2.081 | | |
| Partial or total colectomy (no/yes) | No significant associations with OTUs | | | | |
| Remaining small bowel length (cm) | No significant associations with OTUs | | | | |
| **Growth** | | | | | |
| BMI (SDS) | OTU_212_Genus_Hafnia | 0.078 | -0.654 | | |
| Height-for-age SDS < -2 (no/yes) | OTU_155_Genus_Enterorhabdus | <0.001 | 4.046 | | |
| | OTU_10_Family_Bacteroidales S24-7 group | <0.001 | 4.717 | | |
| | OTU_159_Class_Bacteroidetes VC2.1 Bac22 | <0.001 | 4.359 | | |
| | OTU_209_Genus_Victivallis | <0.001 | 3.551 | | |
| | OTU_213_Order_Gastranaerophilales | <0.001 | 2.947 | | |
| | OTU_41_Genus_Subdoligranulum | 0.001 | 4.128 | | |
| | OTU_116_Genus_Ruminococcaceae UCG-005 | 0.001 | 3.641 | | |
| | OTU_114_Genus_Faecalibacterium | 0.001 | 3.107 | | |
| | OTU_31_Genus_Faecalibacterium | 0.001 | 3.502 | | |
| | OTU_140_Genus_Ruminococcaceae NK4A214 group | 0.001 | 3.032 | | |
| | OTU_230_Genus_Coprobacter | 0.001 | 3.393 | | |
| | OTU_154_Genus_Incertae Sedis | 0.002 | 2.554 | | |
| | OTU_65_Genus_Ruminococcaceae UCG-002 | 0.002 | 3.303 | | |
| | OTU_194_Genus_Butyricimonas | 0.003 | 2.669 | | |
| | OTU_131_Genus_Blautia | 0.005 | 2.287 | | |
| | OTU_44_Genus_[Eubacterium] coprostanoligenes group | 0.005 | 2.808 | | |
| | OTU_42_Genus_Ruminococcus 2 | 0.008 | 3.666 | | |
| | OTU_83_Genus_Ruminococcus 1 | 0.010 | 3.101 | | |
| | OTU_55_Genus_Blautia | 0.011 | 3.268 | | |
| | OTU_247_Family_Coriobacteriaceae | 0.014 | 2.539 | | |
| | OTU_74_Genus_Ruminococcaceae UCG-002 | 0.023 | 3.946 | | |
| | OTU_205_Genus_Alistipes | 0.027 | 3.046 | | |
| | OTU_196_Genus_Ruminococcaceae NK4A214 group | 0.027 | 1.603 | | |
| | OTU_82_Genus_Bacteroides | 0.027 | 4.087 | | |
| | OTU_1_Genus_Escherichia-Shigella | 0.038 | -6.610 | | |
| | OTU_73_Genus_Coprococcus 3 | 0.040 | 2.301 | | |
| | OTU_85_Genus_Lachnospiraceae NK4A136 group | 0.057 | 3.773 | | |
| | OTU_78_Genus_Blautia | 0.067 | 2.014 | | |

| | | | | | |
|---|---|---|---|---|---|
| | OTU_125_Genus_Ruminococcaceae UCG-003 | 0.088 | 2.769 | | |
| | OTU_231_Genus_Leuconostoc | 0.088 | 2.978 | | |
| | OTU_106_Genus_[Eubacterium] hallii group | 0.092 | 2.128 | | |
| Growing outside target height range (no/yes) | OTU_155_Genus_Enterorhabdus | <0.001 | -0.547 | | |
| | OTU_10_Family_Bacteroidales S24-7 group | <0.001 | -0.862 | | |
| | OTU_159_Class_Bacteroidetes VC2.1 Bac22 | <0.001 | -1.086 | | |
| | OTU_209_Genus_Victivallis | <0.001 | -0.561 | | |
| | OTU_41_Genus_Subdoligranulum | <0.001 | -2.196 | | |
| | OTU_213_Order_Gastranaerophilales | <0.001 | -0.581 | | |
| | OTU_116_Genus_Ruminococcaceae UCG-005 | 0.001 | -1.475 | | |
| | OTU_8_Genus_Morganella | 0.002 | 7.298 | | |
| | OTU_114_Genus_Faecalibacterium | 0.004 | 0.455 | | |
| | OTU_157_Genus_Veillonella | 0.004 | 3.589 | | |
| | OTU_140_Genus_Ruminococcaceae NK4A214 group | 0.004 | -0.693 | | |
| | OTU_31_Genus_Faecalibacterium | 0.004 | -0.092 | | |
| | OTU_230_Genus_Coprobacter | 0.005 | 0.342 | | |
| | OTU_154_Genus_Incertae Sedis | 0.006 | -0.487 | | |
| | OTU_65_Genus_Ruminococcaceae UCG-002 | 0.008 | -0.450 | | |
| | OTU_194_Genus_Butyricimonas | 0.011 | -0.189 | | |
| | OTU_55_Genus_Blautia | 0.014 | -1.376 | | |
| | OTU_205_Genus_Alistipes | 0.017 | -1.823 | | |
| | OTU_131_Genus_Blautia | 0.017 | -0.279 | | |
| | OTU_44_Genus_[Eubacterium] coprostanoligenes group | 0.021 | 0.089 | | |
| | OTU_42_Genus_Ruminococcus 2 | 0.022 | -0.671 | | |
| | OTU_83_Genus_Ruminococcus 1 | 0.024 | -0.812 | | |
| | OTU_247_Family_Coriobacteriaceae | 0.038 | -0.485 | | |
| | OTU_196_Genus_Ruminococcaceae NK4A214 group | 0.057 | -0.554 | | |
| | OTU_398_Genus_Ruminococcaceae UCG-014 | 0.057 | -2.758 | | |
| | OTU_74_Genus_Ruminococcaceae UCG-002 | 0.062 | -0.739 | | |
| | OTU_100_Genus_Coprococcus 2 | 0.078 | -1.911 | | |
| | OTU_1_Genus_Escherichia-Shigella | 0.085 | 2.001 | | |
| | OTU_82_Genus_Bacteroides | 0.085 | 0.430 | | |
| **Medication** | | | | | |
| Proton pump inhibitor (no/yes) | OTU_226_Genus_Slackia | 0.002 | -2.362 | | |
| | OTU_207_Genus_Allisonella | 0.002 | -2.476 | | |
| | OTU_99_Genus_Bacteroides | 0.008 | -3.260 | | |
| | OTU_86_Genus_Parasutterella | 0.015 | -2.752 | | |
| | OTU_347_Genus_Lachnoclostridium | 0.024 | -1.504 | | |
| | OTU_18_Genus_Lactobacillus | 0.024 | -2.783 | | |

| | | | | | |
|---|---|---|---|---|---|
| | OTU_128_Genus_Peptoclostridium | 0.024 | -2.429 | | |
| | OTU_88_Family_CFT112H7 | 0.030 | -2.495 | | |
| | OTU_59_Genus_Barnesiella | 0.041 | -1.995 | | |
| | OTU_57_Genus_Subdoligranulum | 0.049 | -1.198 | | |
| | OTU_130_Genus_Acidaminococcus | 0.052 | -1.404 | | |
| | OTU_162_Genus_[Eubacterium] hallii group | 0.058 | -1.022 | | |
| | OTU_1_Genus_Escherichia-Shigella | 0.083 | 3.128 | | |
| Motility agents (no/yes) | No significant associations with OTUs | | | | |
| Cholestyramine (no/yes) | OTU_155_Genus_Enterorhabdus | 0.004 | 1.898 | | |
| | OTU_149_Genus_Lactococcus | 0.004 | 2.065 | | |
| | OTU_128_Genus_Peptoclostridium | 0.004 | 2.763 | | |
| | OTU_86_Genus_Parasutterella | 0.004 | 2.871 | | |
| | OTU_88_Family_CFT112H7 | 0.004 | 3.018 | | |
| | OTU_209_Genus_Victivallis | 0.005 | 1.645 | | |
| | OTU_213_Order_Gastranaerophilales | 0.005 | 1.394 | | |
| | OTU_205_Genus_Alistipes | 0.009 | 1.957 | | |
| | OTU_32_Genus_Bacteroides | 0.010 | 4.475 | | |
| | OTU_268_Genus_Holdemanella | 0.011 | 1.541 | | |
| | OTU_10_Family_Bacteroidales S24-7 group | 0.016 | 2.074 | | |
| | OTU_102_Genus_Odoribacter | 0.036 | 3.071 | | |
| | OTU_277_Genus_Xanthomonas | 0.050 | 1.214 | | |
| | OTU_207_Genus_Allisonella | 0.050 | 1.343 | | |
| | OTU_226_Genus_Slackia | 0.050 | 1.308 | | |
| | OTU_55_Genus_Blautia | 0.065 | 1.866 | | |
| | OTU_139_Genus_Stenotrophomonas | 0.071 | 1.058 | | |
| | OTU_49_Genus_Roseburia | 0.076 | 2.605 | | |
| | OTU_220_Family_Lachnospiraceae | 0.076 | 1.890 | | |
| | OTU_164_Genus_Capnocytophaga | 0.076 | 0.915 | | |
| | OTU_99_Genus_Bacteroides | 0.076 | 1.496 | | |
| | OTU_44_Genus_[Eubacterium] coprostanoligenes group | 0.079 | 1.262 | | |
| | OTU_159_Class_Bacteroidetes VC2.1 Bac22 | 0.097 | 1.346 | | |
| | OTU_196_Genus_Ruminococcaceae NK4A214 group | 0.097 | 0.641 | | |
| | OTU_130_Genus_Acidaminococcus | 0.097 | 1.032 | | |
| | OTU_67_Genus_Collinsella | 0.097 | 1.068 | | |
| Ursochol (no/yes) | OTU_155_Genus_Enterorhabdus | <0.001 | -5.720 | | |
| | OTU_209_Genus_Victivallis | <0.001 | -4.915 | | |
| | OTU_149_Genus_Lactococcus | <0.001 | -4.514 | | |
| | OTU_213_Order_Gastranaerophilales | <0.001 | -3.974 | | |
| | OTU_10_Family_Bacteroidales S24-7 group | <0.001 | -6.477 | | |

| | | | | | |
|---|---|---|---|---|---|
| | OTU_44_Genus_[Eubacterium] coprostanoligenes group | <0.001 | -4.108 | | |
| | OTU_68_Genus_Ruminococcaceae UCG-005 | <0.001 | -4.866 | | |
| | OTU_82_Genus_Bacteroides | 0.011 | -3.635 | | |
| | OTU_606_Genus_Akkermansia | 0.011 | 2.075 | | |
| | OTU_125_Genus_Ruminococcaceae UCG-003 | 0.018 | -3.921 | | |
| | OTU_159_Class_Bacteroidetes VC2.1 Bac22 | 0.021 | -3.218 | | |
| | OTU_25_Genus_Streptococcus | 0.063 | 3.819 | | |
| | OTU_126_Genus_Eggerthella | 0.063 | -3.246 | | |
| | OTU_102_Genus_Odoribacter | 0.073 | -3.850 | | |
| | OTU_42_Genus_Ruminococcus 2 | 0.097 | -3.344 | | |
| Treatment of bacterial overgrowth (no/yes) | OTU_210_Genus_Lactobacillus | 0.010 | 3.482 | | |
| | OTU_277_Genus_Xanthomonas | 0.072 | 1.922 | | |
| | OTU_4_Genus_Lactobacillus | 0.072 | 5.051 | | |
| | OTU_40_Genus_Phascolarctobacterium | 0.092 | 2.072 | | |
| Antibiotics at sample (no/yes)[1] | OTU_946_Genus_Veillonella | 0.024 | -1.515 | | |
| | OTU_244_Genus_Blautia | 0.043 | -1.640 | | |
| | OTU_240_Genus_Tyzzerella | 0.043 | -1.192 | | |
| Antibiotics between samples (no/yes) | OTU_29_Family_Lachnospiraceae | 0.015 | -2.551 | | |
| | OTU_71_Genus_Blautia | 0.015 | -2.301 | | |
| | OTU_92_Genus_Bilophila | 0.015 | -2.327 | | |
| | OTU_220_Family_Lachnospiraceae | 0.054 | -1.703 | | |
| | OTU_96_Genus_Streptococcus | 0.054 | 1.381 | | |
| | OTU_244_Genus_Blautia | 0.055 | -1.505 | | |
| | OTU_207_Genus_Allisonella | 0.055 | -1.164 | | |
| | OTU_226_Genus_Slackia | 0.055 | -1.133 | | |
| | OTU_99_Genus_Bacteroides | 0.073 | -1.416 | | |
| | OTU_137_Genus_Lachnospiraceae UCG-008 | 0.079 | -0.891 | | |
| | OTU_204_Genus_Ruminiclostridium | 0.094 | -1.238 | | |
| | OTU_148_Family_Ruminococcaceae | 0.094 | -0.944 | | |
| | OTU_23_Genus_Alistipes | 0.094 | -1.927 | | |
| **Line sepsis[2]** | No significant associations with OTUs | | | | |

**Legend:** [1] Due to bacterial overgrowth, line sepsis or another cause. [2] with a range of two months before and 2 months after sample collection.

For categorical variables, the first group mentioned in the variable column is assigned as baseline. The beta coefficient mentioned is the coefficient of the second group, the second beta coefficient is the coefficient of the third group and the third coefficient is the coefficient of the fourth group.

**Abbreviations:** BMI, body mass index; PN, parenteral nutrition; REE, resting energy expenditure; SDS, standard deviation score.

**Supplementary Table 12.** Permutation ANOVA for the relation between disease characteristics and microbiota community structure (β diversity) taking into account multiple samples per patient

| **Variable** | **$R^2$** | **p-value** |
|---|---|---|
| **Nutrition** | | |
| Duration of PN (y) | 0.06 | 0.005 |
| Type of nutrition (PN, PN±tubefeeding, PN+oral nutrition±tube feeding, tube feeding/oral nutrition) | 0.15 | 0.039 |
| PN dependency (% of total energy intake) | 0.06 | 0.005 |
| Calories of PN divided by REE (%) | 0.05 | 0.004 |
| Oral nutrition (no/yes) | 0.04 | 0.522 |
| Fiber intake per kg (g/kg) | 0.05 | 0.011 |
| Tube feeding (no/yes) | 0.07 | 0.221 |
| Tube feeding type (polymeric, semi-elemental, elemental) | 0.11 | 0.339 |

| | | |
|---|---|---|
| Mode of tube feeding (continuous, bolus, both) | 0.09 | 0.474 |
| **Growth** | | |
| BMI (SDS) | 0.03 | 0.965 |
| Height-for-age SDS < -2 | 0.03 | 1.000 |
| Growing outside target height range (no/yes) | 0.04 | 1.000 |
| **Gastro-intestinal characteristics** | | |
| Whole small bowel in situ (no/yes) | 0.06 | 1.000 |
| Remaining small bowel length (cm) | 0.06 | 1.000 |
| Ileocecal valve in situ (no/yes) | 0.08 | 1.000 |
| Partial or total colectomy (no/yes) | 0.07 | 0.308 |
| **Medication** | | |

| | | |
|---|---|---|
| Proton pump inhibitor (no/yes) | 0.07 | 0.209 |
| Motility agents (no/yes) | 0.08 | 0.098 |
| Cholestyramine (no/yes) | 0.04 | 0.869 |
| Ursochol (no/yes) | 0.02 | 0.673 |
| Antibiotics at sample (no/yes)* | 0.05 | 0.005 |
| Antibiotics between samples (no/yes) | 0.04 | 0.020 |
| **Line sepsis**** | 0.02 | 0.134 |

**Legend:** *Due to bacterial overgrowth, line sepsis or another cause. **with a range of two mo before and 2 mo after sample collection

**Abbreviations:** BMI, body mass index; PN, parenteral nutrition; REE, resting energy expenditure; SDS, standard deviation score.

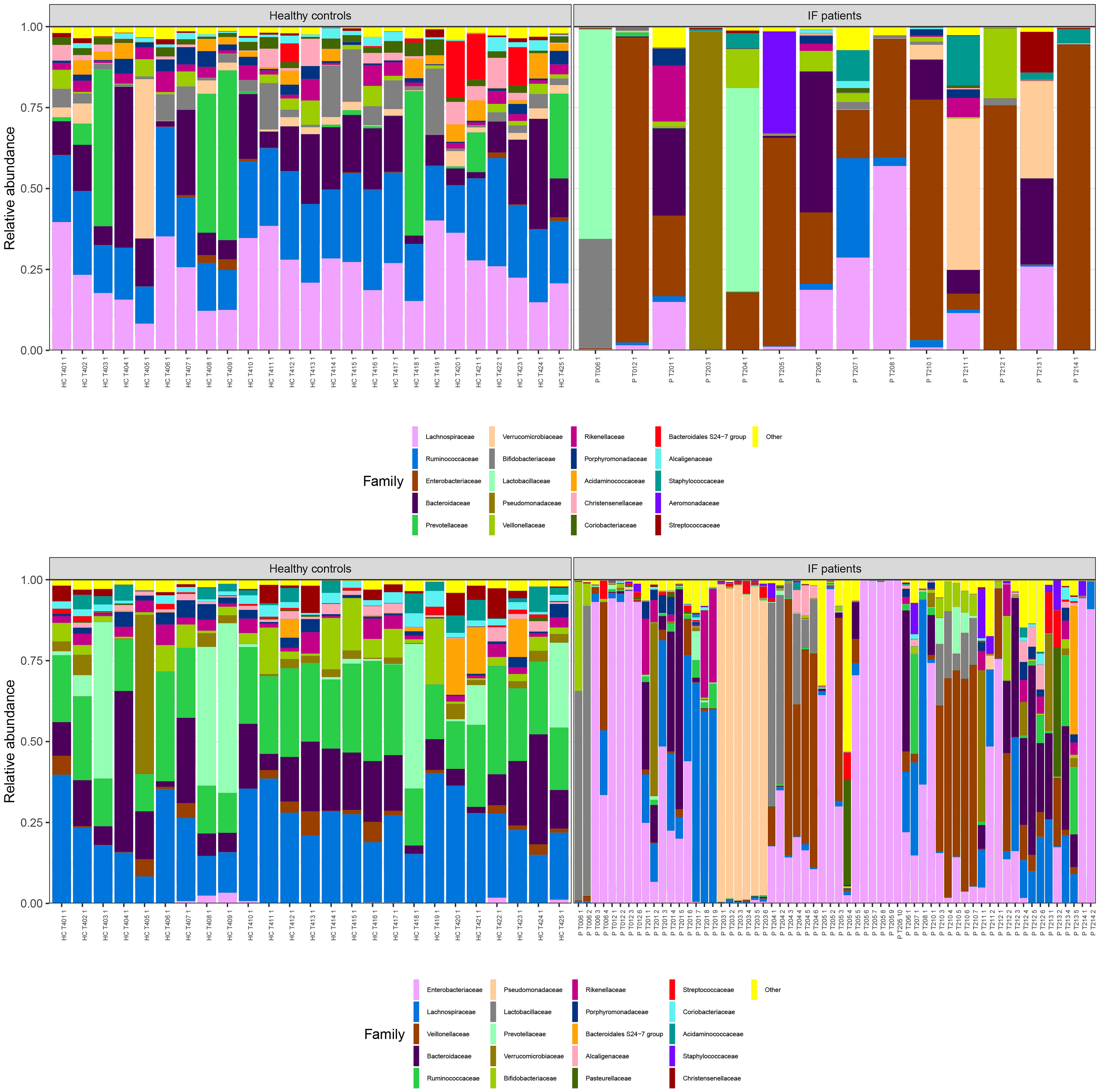


**Supplementary Figure 1**. Taxonomic composition of microbiota of healthy controls (left) and pediatric IF patients (right) at family level at first sample (above) and for all samples (below).

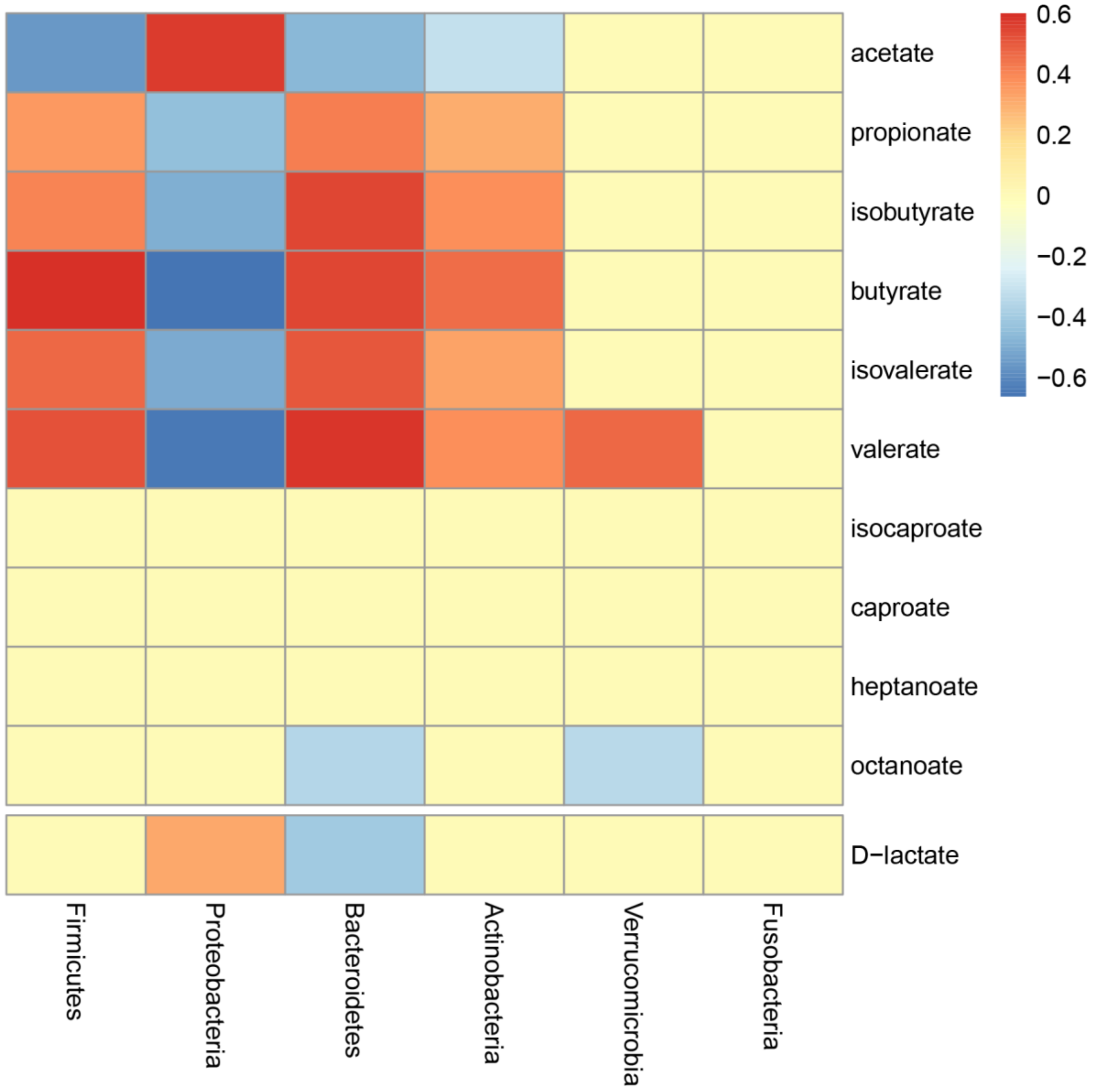


**Supplementary Figure 2.** Heatmap of correlations between the main 6 phyla of the gut microbiota and short-chain fatty acids and D-lactate (both per gram dry feces).

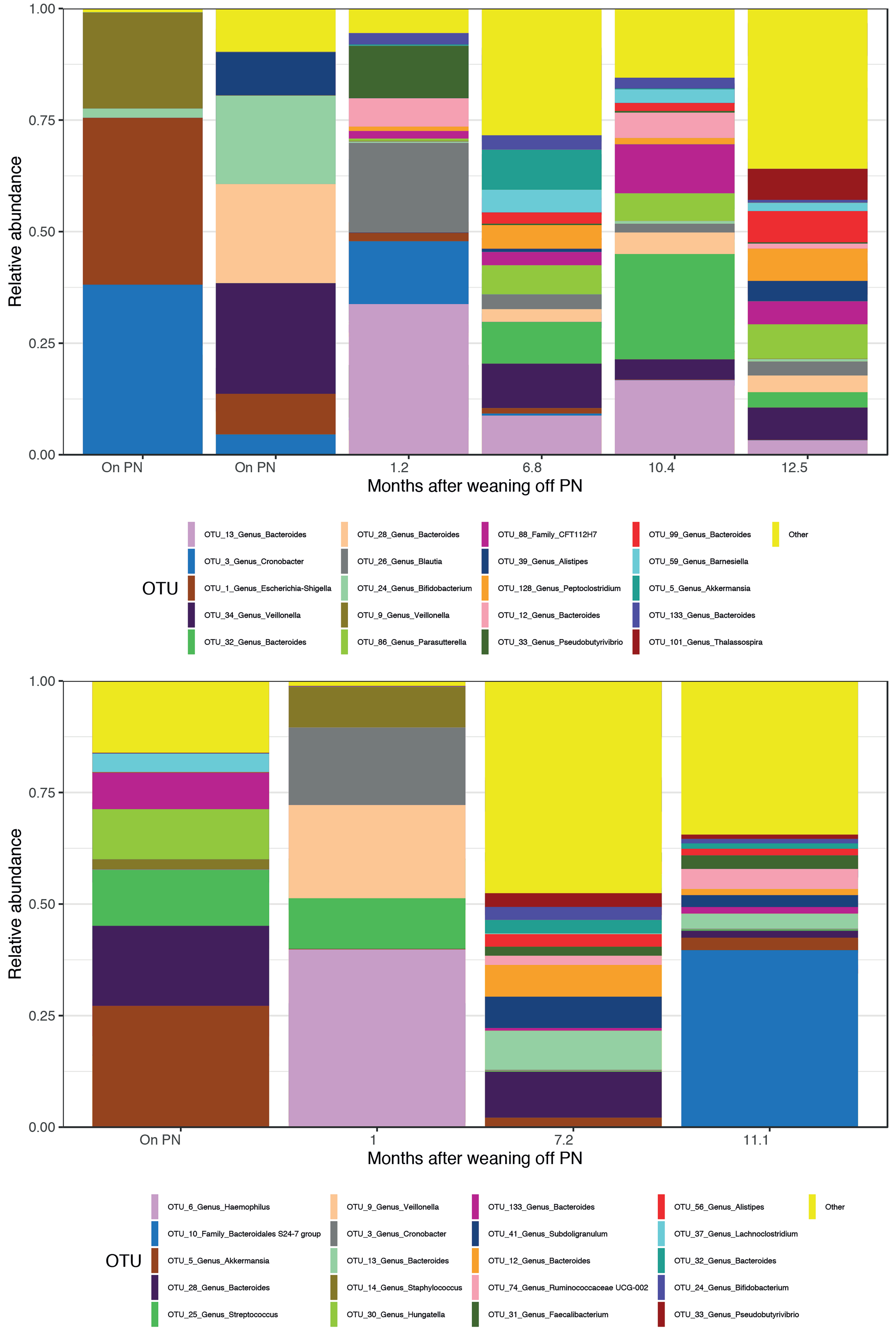


**Supplementary Figure 3**. Example of longitudinal changes at operational taxonomic unit (OTU) level for two patients during the study period. Both patients were stable on parenteral nutrition during this time. The patient on the upper part had surgical IF, whereas the patient below had functional IF.
